# Fermentation-induced variation in heat and oxidative stress phenotypes of *Lactococcus lactis* MG1363 reveals transcriptome signatures for robustness

**DOI:** 10.1186/s12934-014-0148-6

**Published:** 2014-11-04

**Authors:** Annereinou R Dijkstra, Wynand Alkema, Marjo JC Starrenburg, Jeroen Hugenholtz, Sacha AFT van Hijum, Peter A Bron

**Affiliations:** Kluyver Centre for Genomics of Industrial Fermentation, P.O. Box 5057, 2600 Delft, GA The Netherlands; NIZO food research, P.O. Box 20, 6710 Ede, BA The Netherlands; Universiteit van Amsterdam, Swammerdam Institute for Life Sciences, Science Park 904, 1098 Amsterdam, XH The Netherlands; Centre for Molecular and Biomolecular Informatics, Radboud University Medical Center, P.O. Box 9101, 6500 Nijmegen, HB The Netherlands; TI Food & Nutrition, Nieuwe Kanaal 9A, 6709 Wageningen, PA The Netherlands

**Keywords:** Lactic acid bacteria, Stress survival, Transcriptome-phenotype matching, Cysteine, Spray drying

## Abstract

**Background:**

*Lactococcus lactis* is industrially employed to manufacture various fermented dairy products. The most cost-effective method for the preservation of *L. lactis* starter cultures is spray drying, but during this process cultures encounter heat and oxidative stress, typically resulting in low survival rates. However, viability of starter cultures is essential for their adequate contribution to milk fermentation, supporting the ambition to better understand and improve their robustness phenotypes.

**Results:**

This study describes a transcriptome-phenotype matching approach in which the starter *L. lactis* MG1363 was fermented under a variety of conditions that differed in the levels of oxygen and/or salt, as well as the fermentation pH and temperature. Samples derived from these fermentations in the exponential phase of bacterial growth were analyzed by full-genome transcriptomics and the assessment of heat and oxidative stress phenotypes. Variations in the fermentation conditions resulted in up to 1000-fold differences in survival during heat and oxidative stress. More specifically, aeration during fermentation induced protection against heat stress, whereas a relatively high fermentation temperature resulted in enhanced robustness towards oxidative stress. Concomitantly, oxygen levels and fermentation temperature induced differential expression of markedly more genes when compared with the other fermentation parameters. Correlation analysis of robustness phenotypes and gene expression levels revealed transcriptome signatures for oxidative and/or heat stress survival, including the *metC-cysK* operon involved in methionine and cysteine metabolism. To validate this transcriptome-phenotype association we grew *L. lactis* MG1363 in the absence of cysteine which led to enhanced robustness towards oxidative stress.

**Conclusions:**

Overall, we demonstrated the importance of careful selection of fermentation parameters prior to industrial processing of starter cultures. Furthermore, established stress genes as well as novel genes were associated with robustness towards heat and/or oxidative stress. Assessment of the expression levels of this group of genes could function as an indicator for enhanced selection of fermentation parameters resulting in improved robustness during spray drying. The increased robustness after growth without cysteine appeared to confirm the role of expression of the *metC-cysK* operon as an indicator of robustness and suggests that sulfur amino acid metabolism plays a pivotal role in oxidative stress survival.

**Electronic supplementary material:**

The online version of this article (doi:10.1186/s12934-014-0148-6) contains supplementary material, which is available to authorized users.

## Background

*Lactococcus lactis* is important during milk fermentations, as this lactic acid bacterium (LAB) positively contributes to the preservation, flavor and texture characteristics of the resulting fermented end-products, which include cheese and butter(milk) [1]. Nowadays, these milk fermentation processes have largely been industrialized and standardized, and are initiated with the addition of starter cultures containing high concentrations of one or multiple *L. lactis* strains. During production and processing, these starter cultures are exposed to severe stresses, e.g., heat and oxidative stress during spray drying, typically resulting in decreased viability [2-4]. However, viability of starter cultures is essential for their adequate contribution to milk fermentation, supporting the ambition to better understand and improve their robustness phenotypes [5].

One of the most extensively studied *L. lactis* strains is *L. lactis* subsp. *cremoris* strain MG1363, a plasmid free derivative of strain NCDO712 [6]. Various genetic engineering tools have been developed for this strain, which have for example been applied for metabolic engineering strategies [7] and heterologous protein production [8]. In 2007, the complete genome sequence of MG1363 became publicly available [9], allowing the design of DNA microarrays and subsequent full-genome transcriptome analyses. So far, transcriptome analyses of strain MG1363 revealed the regulatory networks of nitrogen metabolism [CodY [10]], carbon catabolite repression [CcpA [11]] and fatty acid biosynthesis [FabT [12]], and this technology was also employed to study gene expression during growth in milk [13].

Although specific genes involved in heat stress [14-16] and/or oxidative stress survival [15,17,18] were studied in MG1363, an analysis of the full-genome transcriptome of this strain in relation to stress survival is, to the best of our knowledge, currently lacking. Nevertheless, pre- and cross-adaptation experiments, in which cells were challenged with a mild stress before exposure to lethal doses of the same or a different stress, respectively, have demonstrated that fermentation conditions prior to stress exposure can affect survival [15,19].

In this study, we fermented strain MG1363 under twelve different conditions and subsequently performed transcriptome analysis, paralleled by heat and oxidative stress survival measurements. This approach revealed the effect of fermentation conditions on stress survival and gene expression, and also enabled transcriptome-phenotype matching [20] in which gene expression profiles were correlated to survival characteristics, revealing transcriptome signatures for robustness.

## Results

### Fermentation conditions strongly affect robustness

**Figure 1:**
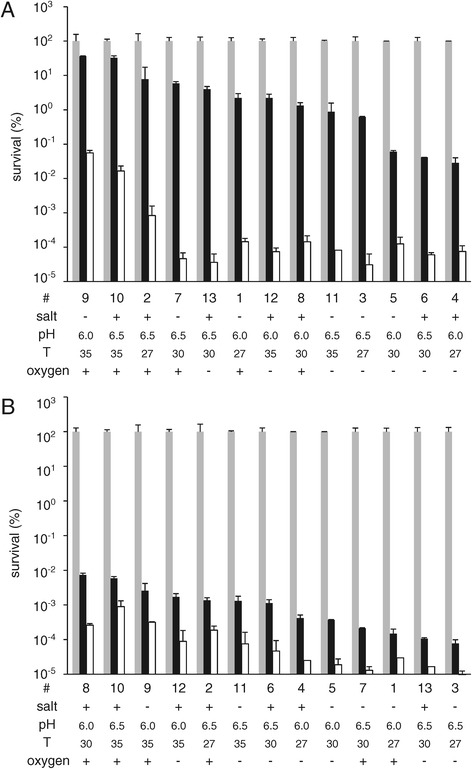
**Robustness of MG1363 at various fermentation conditions.** Survival percentage of strain MG1363 at t = 0 (grey bars), 30 minutes (black bars) and 60 minutes (white bars) of heat stress **(A)** or oxidative stress **(B)**. Fermentation numbers are as indicated in Table 1. Fermentations varied in addition of salt (0 [−] or 100 [+] mM NaCl), starting pH (6.0 or 6.5), fermentation temperature (27, 30 or 35°C) and level of oxygen (shaken [+] or static [−]). Data represent averages of technical duplicates. Error bars indicate standard deviation.

Strain MG1363 was fermented under twelve different conditions, resulting in variation in growth characteristics, including maximum growth rate (μ_max_) and final optical density (OD_final_, [Table 1, Additional file 1]). Fermentation at 35°C and a starting pH of 6.5 without aeration and addition of salt resulted in the highest maximum growth rate (1.21 h^−1^ [Table 1]), whereas fermentations at 27°C in the presence of relatively high levels of oxygen resulted in the lowest maximum growth rate (0.73 h^−1^). Overall, the maximum growth rate was significantly lower in fermentations at 27°C than in fermentations at 30°C or 35°C. The OD at the end of fermentation ranged from 1.76 to 3.28 (Table 1) and was significantly higher in fermentations started at pH 6.5 than in fermentations started at pH 6.0. In exponential phase of growth, cells were harvested and assessed for heat and oxidative stress survival phenotypes. Variations in fermentation conditions resulted in up to 1000-fold differences in survival (Figure 1), which did not correlate with the observed maximum growth rate or optical density at the end of fermentation. The most robust phenotypes towards heat stress were measured in cells from fermentations at 35°C with a relatively high level of oxygen (fermentation numbers 9 and 10, Figure 1A). Cells from these fermentations also displayed a high robustness towards oxidative stress (Figure 1B). Concomitantly, significant correlation between heat stress survival (at 60 minutes) and oxidative stress survival (at both time points) was observed, which corresponds with our previous observation that robustness towards heat and oxidative stress are significantly correlated [4]. Furthermore, growth curves and robustness levels after 60 minutes of the duplicate samples 6 and 13 were highly similar (Figure 1 and Additional file 1). However, robustness levels after 30 minutes of stress of these duplicate samples displayed some variation (approximately one log unit; Figure 1) and, therefore, samples 6 and 13 were treated as individual samples in further analyses. Nevertheless, for both heat and oxidative stress, robustness after 30 minutes of stress correlated significantly with robustness after 60 minutes of stress, indicating both time points appear to appropriately represent the fermentation-induced differences in survival.

**Table 1:** **Fermentation conditions and resulting growth characteristics of MG1363**

**Fermentation number**	**NaCl (mM)**	**Starting pH**	**Temperature (°C)**	**Level of oxygen**	**OD**_**final**_	**μ**_**max**_ **(h** ^**−1**^ **)**
1	0	6.0	27	+	1.95	0.73
2	100	6.5	27	+	2.68	0.73
3	0	6.5	27	-	3.04	0.94
4	100	6.0	27	-	1.76	0.83
5	0	6.0	30	-	2.18	1.06
6	100	6.5	30	-	2.72	1.01
7	0	6.5	30	+	3.28	0.95
8	100	6.0	30	+	2.05	0.82
9	0	6.0	35	+	2.37	1.01
10	100	6.5	35	+	3.17	0.98
11	0	6.5	35	-	3.06	1.21
12	100	6.0	35	-	2.02	1.09
13	100	6.5	30	-	2.78	1.00

**Figure 2:**
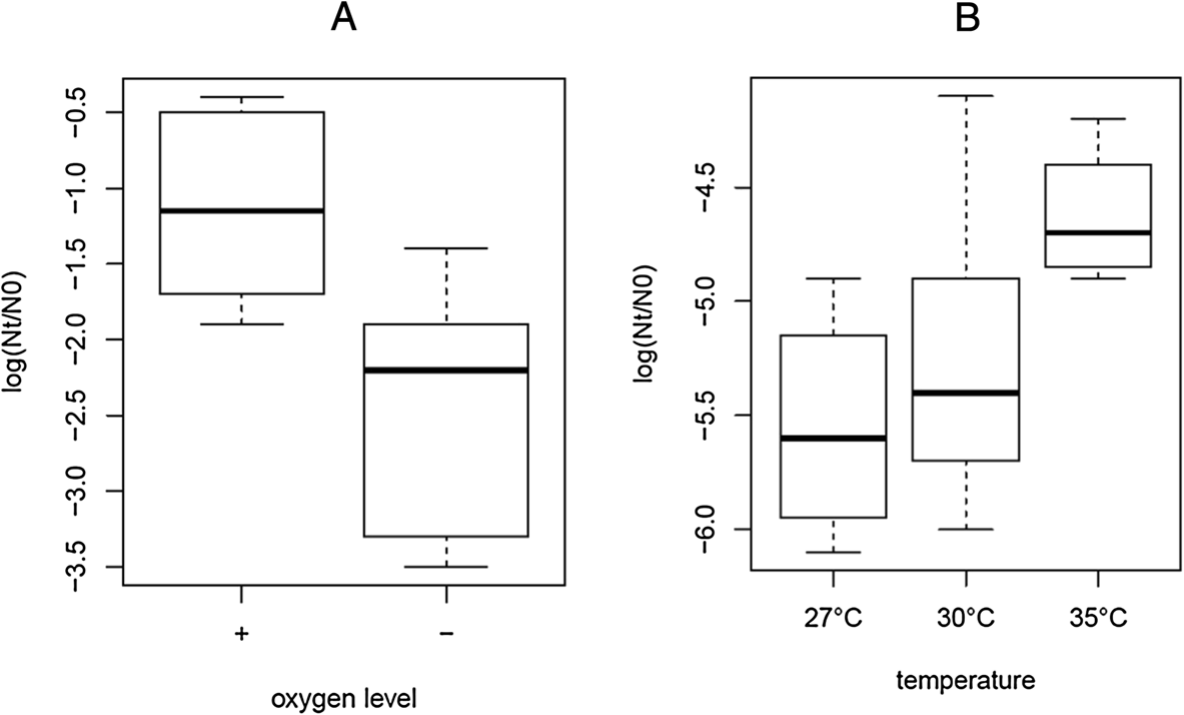
**Effect of oxygen level and temperature on robustness of MG1363.** Boxplots of robustness phenotypes after 30 minutes of heat stress at high (+) and low (−) oxygen levels **(A)** and robustness phenotypes after 30 minutes of oxidative stress at various temperatures **(B)**. Robustness is expressed as the difference of log CFU/ml after stress (Nt) and before stress (N0).

To identify which individual fermentation parameter had the most pronounced effect on robustness, we compared robustness in all fermentations with one variant of an individual parameter (e.g. pH 6.0) with robustness in all fermentations with the other variant of this fermentation parameter (e.g. pH 6.5). Fermentations varying in salt concentration or pH did not significantly differ in robustness towards heat or oxidative stress (Additional file 2). By contrast, the oxygen level and the fermentation temperature appeared to be relevant parameters for robustness. Aeration due to shaking during fermentation resulted in significantly improved robustness towards heat stress (Figure 2A), whereas fermentation at 35°C resulted in improved robustness towards oxidative stress when compared with the fermentations performed at 27°C (Figure 2B). Taken together, our data demonstrate that fermentation conditions have a dramatic impact on stress survival phenotypes and provide clues for optimization of fermentation processes for enhanced robustness.

### Transcriptome-phenotype matching identifies genetic signatures for robustness

**Figure 3:**
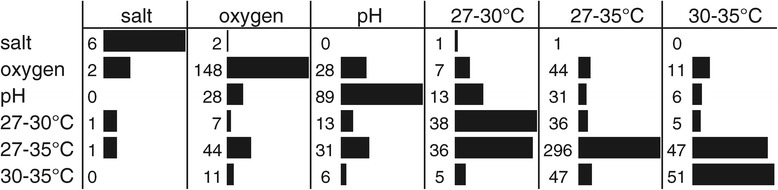
**Differentially expressed genes by fermentation parameters.** Differentially expressed genes (*P* <0.05) by individual fermentation parameters (salt, oxygen, pH and temperature). Numbers indicate the number of genes that are differentially expressed by both the fermentation parameter specified in the top row and in the left column. Bars indicate percentages of overlap of differentially expressed genes by both fermentation parameters (no bar = 0%, full bar = 100%).

To determine which transcriptome changes are associated with the variations in fermentation conditions and corresponding robustness phenotypes, we initially assessed the effect of the individual fermentation parameters on gene expression. Aeration (148 genes) and alteration in temperature (296 genes when comparing the highest and lowest temperature) significantly altered the expression of a high number of genes (Figure 3) as compared with the fermentation parameters salt (6 genes) and pH (89 genes), which appears in line with the observation that alteration of oxygen level and fermentation temperature also has the most pronounced effect on robustness phenotypes. Overlap of differentially expressed genes between the various temperature gradients indicates that expression of most genes is gradually altered during the stepwise increase in temperature.

**Figure 4:**
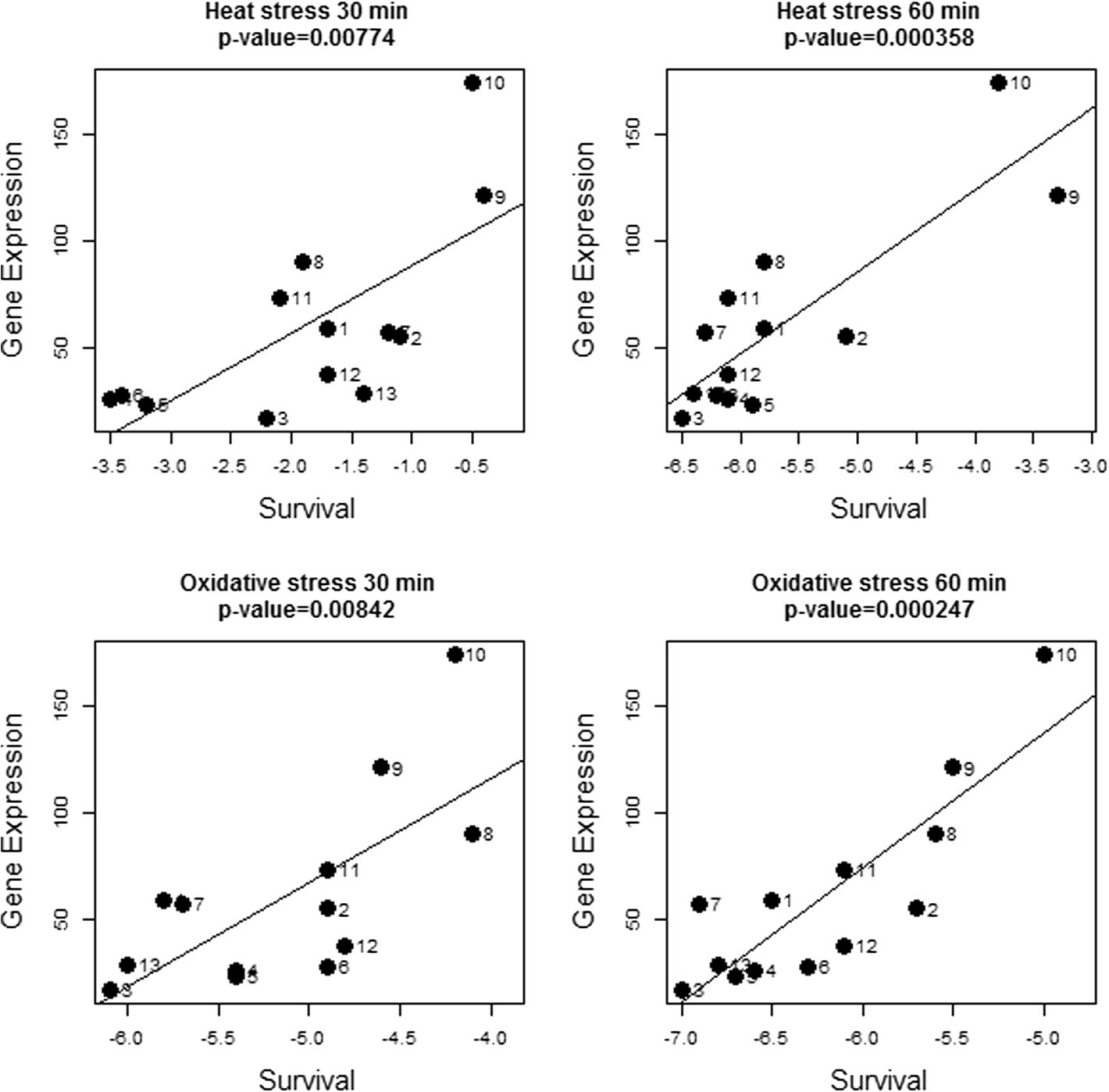
**Correlation of** ***cysK*** **gene expression and robustness levels.** Gene expression of *cysK* plotted against robustness towards 30 minutes and 60 minutes of heat stress and towards 30 minutes and 60 minutes of oxidative stress. Survival is expressed as the difference of log CFU/ml after stress and before stress. Numbers indicate fermentations as presented in Table 1.

To assess which gene expression levels associate with robustness, we calculated the correlation of gene expression in the various fermentations with the corresponding robustness towards heat as well as oxidative stress (Additional file 3A and B). From a total of 2369 genes, expression of 632 and 149 genes displayed a significant correlation (according to a linear model) with survival after 30 and 60 minutes of heat stress, respectively. The number of genes significantly correlating with robustness towards oxidative stress was 346 and 249 for 30 and 60 minutes of stress, respectively. For further analysis, we focused on gene expressions displaying a significant correlation (*P* <0.05) with robustness at both time points of the stress assays. The significance of correlation at both time points was combined by calculating the product of both *P*-values, which was used to select the genes with the most significant correlation (product of *P*-values <5 × 10^-5^ [Table 2A and B]). For heat stress and oxidative stress, 18 and 54 genes met these criteria, respectively, and most of them displayed a positive correlation with robustness (13 and 37 genes, respectively). Expression of five genes displayed correlation with both heat and oxidative stress survival, including two genes involved in sulfur amino acid metabolism, *metC* and *cysK* (Figure 4). Furthermore, gene *llmg_1094*, encoding a hypothetical protein, and a gene encoding an ABC transporter permease (*llmg_1494*) positively correlated with robustness towards both stresses. Expression of the gene *pepO2*, encoding an endopeptidase, negatively correlated with both heat and oxidative stress survival.

**Table 2:** **Gene expressions correlating with robustness**

**A**			
**Locus tag**	**Gene**	**Function**	**Correlation**
***llmg_1775***	*cysK*	O-acetylserine sulfhydrylase	Positive
***llmg_1776***	*metC*	Cystathionine gamma-synthase/cystathionine beta-lyase	Positive
*llmg_0091*	*cysD*	O-acetylhomoserine sulfhydrylase	Positive
*llmg_1150*		Hypothetical protein	Negative
***llmg_1494***		ABC transporter permease	Positive
*llmg_0488*		Multiple sugar-binding protein precursor	Positive
*llmg_1718*	*uvrC*	Excinuclease ABC subunit C	Positive
***llmg_1094***		Hypothetical protein	Positive
*llmg_0199*	*feoB*	Ferrous iron transport protein B-like protein	Positive
*llmg_1388*		Hypothetical protein	Negative
*llmg_1042*	*trpA*	Tryptophan synthase subunit alpha	Positive
*llmg_0346*	*fhuC*	Ferrichrome ABC transporter fhuC	Positive
*llmg_0883*		Hypothetical protein	Positive
*llmg_1503*		Surface protein	Negative
*llmg_1662*	*uspA*	Universal stress protein A	Positive
*llmg_0428*	*fni*	Isopentenyl pyrophosphate isomerase	Positive
***llmg_1985***	*pepO2*	Putative neutral endopeptidase O2	Negative
*llmg_1275*	*aldB*	AldB protein	Negative
**B**			
**Locus tag**	**Gene**	**Function**	**Correlation**
*llmg_1447*		Hypothetical protein	Positive
*llmg_0085*		ABC transporter ATP-binding protein	Positive
*llmg_1770*	*noxC*	NADH oxidase	Positive
*llmg_1641*	*butA*	Acetoin reductase	Positive
*llmg_0339*	*dar*	Acetoin(diacetyl)reductase	Positive
*llmg_2448*	*pgi*	Glucose-6-phosphate isomerase	Negative
***llmg_1094***		Hypothetical protein	Positive
*llmg_0003*	*rexB*	ATP-dependent nuclease subunit B	Positive
*llmg_0885*	*proB*	Gamma-glutamyl kinase	Positive
*llmg_0557*	*prfA*	Peptide chain release factor 1	Positive
***llmg_1775***	*cysK*	O-acetylserine sulfhydrylase	Positive
*llmg_0519*	*tig*	Trigger factor	Negative
*llmg_0960*		Beta-glucosidase	Positive
*llmg_0402*	*pbp1B*	Penicillin-binding protein 1B	Positive
*llmg_0755*		Hypothetical protein	Positive
*llmg_0959*		Beta-glucosidase	Positive
*llmg_1928*	*kinG*	Sensor histidine kinase	Positive
*llmg_1095*		Hypothetical protein	Positive
***llmg_1494***		ABC transporter permease	Positive
*llmg_1051*		Transcription regulator	Negative
***llmg_1776***	*metC*	Cystathionine gamma-synthase/cystathionine beta-lyase	Positive
*llmg_0485*		Hypothetical protein	Positive
*llmg_0592*		Hypothetical protein	Positive
*llmg_1317*		N-acetylmannosamine-6-phosphate 2-epimerase	Positive
*llmg_0403*	*pepA*	glutamyl-aminopeptidase	Negative
*llmg_1957*		ABC transporter, ATP-binding protein	Negative
*llmg_1093*		Putative secreted protein	Positive
*llmg_2047*		Universal stress protein E	Negative
*llmg_0556*		Hypothetical protein	Positive
*llmg_0909*	*kinA*	Sensor protein kinase kinA	Negative
*llmg_1642*	*butB*	2,3-butanediol dehydrogenase	Positive
*llmg_0870*		Transporter	Negative
*llmg_1181*	*ilvE*	Branched-chain amino acid aminotransferase	Negative
*llmg_1830*	*menX*	Menaquinone biosynthesis related protein	Positive
*llmg_0487*		Putative trehalose/maltose hydrolase	Positive
*llmg_0066*	*araT*	Aromatic amino acid aminotransferase	Negative
*llmg_1961*	*tenA*	Transcriptional activator TenA	Negative
*llmg_1387*		Hypothetical protein	Negative
*llmg_1091*		Putative secreted protein	Positive
*llmg_1582*		Hypothetical protein	Negative
*llmg_0722*	*serS*	Seryl-tRNA synthetase	Negative
***llmg_1985***	*pepO2*	Putative neutral endopeptidase O2	Negative
*llmg_0205*		Hypothetical protein	Negative
*llmg_2035*	*gidA*	tRNA uridine 5-carboxymethylaminomethyl modification enzyme GidA	Positive
*llmg_1098*	*glpD*	GlpD protein	Positive
*llmg_1638*	*mleS*	Malate dehydrogenase	Positive
*llmg_1128*		Hypothetical protein	Positive
*llmg_0908*	*llrA*	Two-component system regulator llrA	Negative
*llmg_0760*		Putative transglycosylase	Positive
*llmg_2039*		Ribonuclease III	Positive
*llmg_0383*	*ppnK*	Inorganic polyphosphate/ATP-NAD kinase	Positive
*llmg_1096*		Hypothetical protein	Positive
*llmg_1090*		Hypothetical protein	Positive
*llmg_1031*	*trpG*	Anthranilate synthase component II	Positive

Correlating gene expressions with robustness towards heat stress (A) or oxidative stress (B). Genes of which expression levels correlate with robustness towards both heat and oxidative stress are bold.

Among the genes of which expression correlated only with heat stress survival was *uspA*, encoding for the universal stress protein A, and an additional gene encoding a function involved in sulfur amino acid metabolism (*cysD*). Furthermore, expression of genes involved in iron (complex) transport (*feoB* and *fhuC*) positively correlated with robustness towards heat stress.

For oxidative stress, expression of three genes involved in acetoin metabolism (*dar*, *butA* and *butB*) displayed a positive correlation with robustness. Furthermore, the genetically linked (i.e. part of a group of genes that are adjacent and colinear and which have an intergenic spacing smaller than 100 bp) genes *llmg_1090* and *llmg_1091*, encoding a hypothetical protein and putative secreted protein respectively, displayed a positive correlation of expression and robustness towards oxidative stress, as well as the nearby genetically linked genes *llmg_1093*, *llmg_1094* and *llmg_1095*, encoding a putative secreted protein and hypothetical proteins. Genes of which expression negatively correlated with robustness towards oxidative stress encoded for a two component signal transduction system (*llrA* and *kinA*) and for the universal stress protein E (*llmg_2047*). Furthermore, two genes encoding aminotransferases (*ilvE* and *araT*) negatively correlated with oxidative stress survival. These data demonstrate that the applied fermentation conditions affected gene expression levels, enabling correlation with the corresponding robustness phenotypes, resulting in transcriptome signatures associated with robustness towards heat and oxidative stress.

### Absence of cysteine during growth increases robustness towards oxidative stress

**Figure 5:**
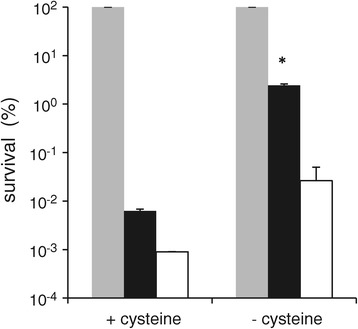
**Oxidative stress survival of MG1363 after growth with or without cysteine.** Survival of strain MG1363 grown in CDM with cysteine (+ cysteine) or without cysteine (− cysteine) after 0 minutes (grey bars), 30 minutes (black bars) and 60 minutes (white bars) of oxidative stress. Data represent averages of two biological replicates. Error bars indicate standard deviations. *P-value <0.05 (*t*-test, compared with 0 minutes of stress).

The genes *metC* and *cysK*, encoding a cystathionine γ-synthase/cystathionine β-lyase and an *O*-acetylserine sulfhydrylase, respectively, were both associated with heat as well as oxidative stress survival (Table 2). These genes are co-transcribed [21], and this operon was described to be induced by sulfur starvation, particularly during low cysteine conditions, resulting in an increase in cystathionine β-lyase activity [22]. To assess if sulfur starvation could also induce an increase in robustness, MG1363 was fermented in chemically defined medium (CDM) without cysteine. Although an increased cystathionine β-lyase activity was observed in cells grown in CDM without cysteine, no difference in robustness towards heat stress due to absence of cysteine was observed (data not shown). However, cells grown in CDM lacking cysteine displayed an over 100-fold increased robustness towards oxidative stress as compared with cells grown in CDM with cysteine (Figure 5). These experiments demonstrate that association of transcriptome data with robustness phenotypes can pinpoint beneficial modifications in the fermentation process resulting in enhanced survival and, concomitantly, increased activity of starter cultures.

## Discussion

Fermentation conditions were demonstrated to highly impact (up to 1000-fold) on subsequent survival of *L. lactis* MG1363 during heat and oxidative stress, indicating that a careful selection of fermentation conditions prior to industrial processing is important for optimal survival of starter cultures prior to their application in (milk) fermentations. The use of specific fermentation conditions resulting in more robust phenotypes are not necessarily more time-consuming, since increased robustness did not correlate with decreasing growth rates. Thus, these robustness-enhancing measures appear straight-forward to be implemented in an industrial setting. Of all individual fermentation parameters that were exploited, temperature and oxygen level had the most pronounced effect on both gene expression and robustness. Instead of pre-adaptation, as has previously been demonstrated in MG1363 [15], cross-adaptation was observed. Aeration resulted in protection against heat stress, whereas a high temperature during fermentation resulted in increased robustness towards oxidative stress. This supports previous studies, which demonstrated that survival mechanisms towards heat and oxidative stress are (partially) overlapping in MG1363 [19,23]. Exploiting this overlap might be useful for improving robustness during spray drying, as both robustness towards heat stress and robustness towards oxidative stress contribute to survival during spray drying [4]. As we applied a combinatorial design, in which the impact of individual fermentation parameters is assessed in a background of multiple other parameters that are varied, it is more likely that the information obtained here is also relevant for the optimization of other (industrial) fermentation processes.

Gene expression levels and heat and/or oxidative stress survival were correlated, resulting in a set of genes of which expression is associated with robustness. This set included novel genes as well as genes that were previously demonstrated to be involved in stress. For example, the two-component system *kinA*/*llrA*, of which expression correlated with oxidative stress survival, is also involved in acid stress [18]. Expression of the genes *cysK* and *metC*, which both correlated with heat as well as oxidative stress survival was previously demonstrated to be altered during heat stress in *L. lactis* strain IL1403 [24]. In the same study, the oxidative stress survival associated genes *araT* and *mleS* were demonstrated to be differentially expressed during heat stress and both heat and salt stress, respectively [24]. Furthermore, induction of the gene *uspA*, encoding universal stress protein A, which was correlating with heat stress survival in our study, was also shown to correlate with heat tolerance in *Escherichia coli* [25]. Multiple genes (*dar*, *butA* and *butB*) involved in acetoin metabolism displayed correlation of expression and robustness towards oxidative stress. This is in line with previous studies in which production of diacetyl and acetoin in *L. lactis* was demonstrated to be influenced by oxygen concentrations and to be connected to NADH oxidase activity [26,27].

The transcript levels of *cysK* and *metC* positively correlated with robustness towards both heat and oxidative stress. We initially assessed the role of the *metC*-*cysK* operon in robustness by a gene deletion approach, which revealed altered robustness phenotypes, but the lack of an anticipated cysteine auxotrophy in the *cysK* gene deletion mutant [22] and presence of a gene (*llmg_0508*) with a similar function as *cysK* in the genome of MG1363 [28] complicates interpretation of these results (data not shown). Nevertheless, the observation of increased robustness towards oxidative stress after growth in absence of cysteine appears to confirm the role of the *metC*-*cysK* operon as an indicator of robustness. Although absence of cysteine probably results in differential expression of multiple genes, it is expected that the number of genes is limited and that the most pronounced effect will be on genes involved in cysteine and methionine metabolism, as was previously demonstrated in *L. lactis* strain IL1403 [29]. Regardless, absence of cysteine in the medium resulted in an increase in cystathionine β-lyase activity, indicating that expression of the *metC-cysK* operon was indeed increased, in line with earlier experiments [22]. Absence of cysteine did not affect robustness towards heat stress, indicating that the survival mechanism(s) induced by absence of cysteine are specific for oxidative stress. The observed increased robustness towards oxidative stress due to absence of cysteine in the growth medium seems to contrast with the general application of cysteine as a reducing agent in anaerobic fermentation media. However, the absence of a reducing agent in the medium might actually trigger the cells to induce protective mechanisms resulting in an increased robustness towards oxidative stress. The exact mechanism resulting in increased robustness remains unclear. As sulfur-containing amino acids are readily oxidized, it is tempting to speculate that changes in their metabolism influences oxidation/reduction status of the cell. Another possible explanation might be found in the fact that cysteine-derived proteins such as thioredoxin and glutathione were previously demonstrated to be involved in protection against oxidative stress [30,31].

The increased robustness towards oxidative stress after growth in medium lacking cysteine was not only observed in exponential phase, but also in stationary phase (data not shown), indicating that our observations in exponential phase are also relevant for cells harvested from the stationary phase of growth, which is typically the case in industry.

## Conclusions

In this study, we demonstrated the importance of careful selection of fermentation parameters prior to industrial processing of starter cultures. Furthermore, we have shown that correlation of heat and oxidative stress survival phenotypes and gene expression levels can reveal transcriptome signatures for robustness. Established stress genes as well as novel genes were associated with robustness towards heat and/or oxidative stress, which is relevant for survival during spray drying [4]. Assessment of the expression levels of this group of genes could function as an indicator to enable selection of optimized fermentation parameters resulting in enhanced robustness during spray drying. Moreover, we demonstrated that the removal of one single component from the growth medium can have an enormous effect on survival. The increased robustness of MG1363 after growth without cysteine, appears to confirm the role of the *metC*-*cysK* operon as an indicator for robustness and suggests that sulfur amino acid metabolism plays an important role in oxidative stress survival.

## Methods

### Fermentations

*L. lactis* strain MG1363 [6] was pre-cultured overnight at 30°C in 10 ml CDM [32,33]. The composition of the CDM was as described by Wegkamp *et al*. [34], with the following adjustments: 0.35 g/l asparagine instead of 0.42 g/l aspartic acid and 0.0025 instead of 0.001 g/l 6,8-thioctic acid, and the addition of 19 g/l β-glycerophosphate and 0.01 g/l *p*-aminobenzoic acid. Subsequently, 50 ml CDM was inoculated with 1% (v/v) of pre-culture and fermented under twelve different conditions varying in addition of salt (0 or 100 mM sodium chloride [Merck, Darmstadt, Germany]), starting pH (6.0 or 6.5), temperature (27, 30 or 35°C) and level of oxygen (static in 50 ml Falcon tube or shaken at 100 rpm in 500 ml shake flask with cotton plug). A combinatorial design was applied to achieve sufficient replicates to assess the effect of the individual fermentation parameters on robustness and transcriptome with a minimized number of fermentations (Table 1). Fermentations were performed on two separate days (fermentation number 1–6 on day 1, 7–12 on day 2) and, therefore, a replicate of fermentation 6 was added on day 2 (fermentation 13). In exponential phase of growth (OD_600_ between 0.6 and 0.75), cells were harvested for heat and oxidative stress survival assays and RNA isolation.

To assess the effect of cysteine in the medium, MG1363 was grown in CDM with or without cysteine at 30°C at a starting pH of 6.5 without addition of salt or oxygen.

### Heat and oxidative stress survival assays

Stress survival was determined by a method previously developed in our laboratory, with minor modifications [4]. Cells were harvested from 5 ml of culture by centrifugation at 1865 × *g* for 10 minutes and resuspended in 2.5 ml sterile 50 mM sodium phosphate (Merck) buffer pH 7.2. To measure heat stress survival, 0.5 ml of the cell suspensions were diluted twice by adding 0.5 ml of phosphate buffer and were incubated in duplicate in a volume of 0.1 ml at 50°C for 30 and 60 minutes in 0.2 ml PCR tubes (Bioplastics BV, Landgraaf, The Netherlands) in a Gene-Amp PCR system 9600 (Applied BioSystems, Foster City, California, USA). For assessment of oxidative stress survival, hydrogen peroxide (Merck) in phosphate buffer was added to 0.25 ml of the cell suspensions in duplicate to a final concentration of 5 mM and an end volume of 0.5 ml, followed by incubation for 30 and 60 minutes at 30°C in a water bath. After incubation, samples were centrifuged at 15,000 × *g* for 3 minutes and cells were resuspended in 0.5 ml of phosphate buffer. Survival was assessed by spotting serial dilutions in triplicate on M17 agar plates supplemented with 0.5% glucose [35]. Colony forming units (CFU) were determined after incubation of the plates for 72 hours at 30°C.

### RNA isolation and DNA microarrays

RNA isolation, subsequent cDNA synthesis and labeling, as well as DNA microarray hybridizations were performed using routine procedures, as described previously for *Lactobacillus plantarum* by Bron *et al*. [36] with minor adjustments. Aliquots of 5 ml of culture were centrifuged at 4000 × *g* for 3 minutes at 2°C and cells were resuspended in 0.5 ml cold TE buffer. To this suspension, 500 μl 1:1 phenol/chloroform, 30 μl 10% SDS, 30 μl 3 M sodium acetate pH 5.2 and 500 mg 0.1 mm zirconia beads (Biospec Products, Inc., Bartlesville, USA) was added in a 2 ml screw-cap tube and samples were frozen in liquid nitrogen and stored at −80°C. The DNA microarray hybridization scheme contained two connected loops, both containing samples derived on a single day (Additional file 4). A two-dye microarray-based gene expression analysis was performed on a custom-made 60-mer oligonucleotide array (Agilent Technologies, Santa Clara, California, USA, submitted in Gene Expression Omnibus under GEO Series accession number GSE58284) to determine genome-wide gene transcription levels. Co-hybridization of Cy5- and Cy3-labeled cDNA probes was performed on these oligonucleotide arrays at 65°C and 10 rpm for 17 h using GEX HI-RPM buffer (Agilent Technologies). After hybridization, slides were washed twice in Washbuffer#1 (Agilent Technologies) and subsequently in Washbuffer#2 (Agilent Technologies) at 37°C.

### Data analysis

The raw expression data were Lowess normalized and scaled to normalized probe expression levels using MicroPreP [37]. As in general multiple probes were designed for each ORF, the ORF expression level was calculated from the median of its probe signals. Normalized gene expression levels were further analyzed using the R BioConductor package [38]. After 2-log transformation, gene expression levels were plotted against robustness levels and significance of the correlation was assessed by a linear model.

Correlation of survival at the different time points, correlation of survival and growth rate or optical density and correlation of heat stress survival and oxidative stress survival was determined by calculating the Pearson correlation coefficient and corresponding *P*-values. Differences in the effect of individual fermentation parameters on growth characteristics, gene expression and robustness were assessed with a *t*-test. All statistic calculations were done in R (version 3.0.1 [39]). Correlations and differences were considered significant if the *P*-value of the corresponding tests was smaller than 0.05, unless otherwise stated.

## Additional files

Additional file 1:
**Growth curves of MG1363 during various fermentations.** Growth curves of strain MG1363 in fermentations as presented in Table 1. The data points between the dotted lines indicate the moment of harvesting cells for RNA isolation and stress survival assays. Additional file 2:
**Correlation of fermentation parameters and robustness.** Significance of correlation of individual fermentation parameters and robustness phenotypes, indicated by *P*-values (*t*-test, significant differences [*P* <0.05] are underlined). Additional file 3:
**Plots of gene expression and robustness levels.** Expression levels of all genes plotted against survival after 30 and 60 minutes heat and oxidative stress (A: genes *llmg_0001* to *llmg_1229*, B: genes llmg_*1230* to llmg_*2563*). Survival is expressed as the difference of log CFU/ml after stress and before stress. Numbers indicate fermentations as presented in Table 1. *P*-values above the plots indicate significance of correlation (assessed by a linear model). Additional file 4:
**DNA microarray hybridization scheme.** Numbers indicate fermentations as presented in Table 1. Samples connected with arrows were hybridized together, the arrow head represents Cy5-labeling, the back end Cy3-labeling.
