# Supplementary figures and images for: Fermentation-induced variation in heat and oxidative stress phenotypes of Lactococcus lactis MG1363 reveals transcriptome signatures for robustness (part 1 of 3)
Source: Microb Cell Fact. 2014 Nov 4;13:148. doi: 10.1186/s12934-014-0148-6 (PMC4229599; doi:10.1186/s12934-014-0148-6)

## Slide 1
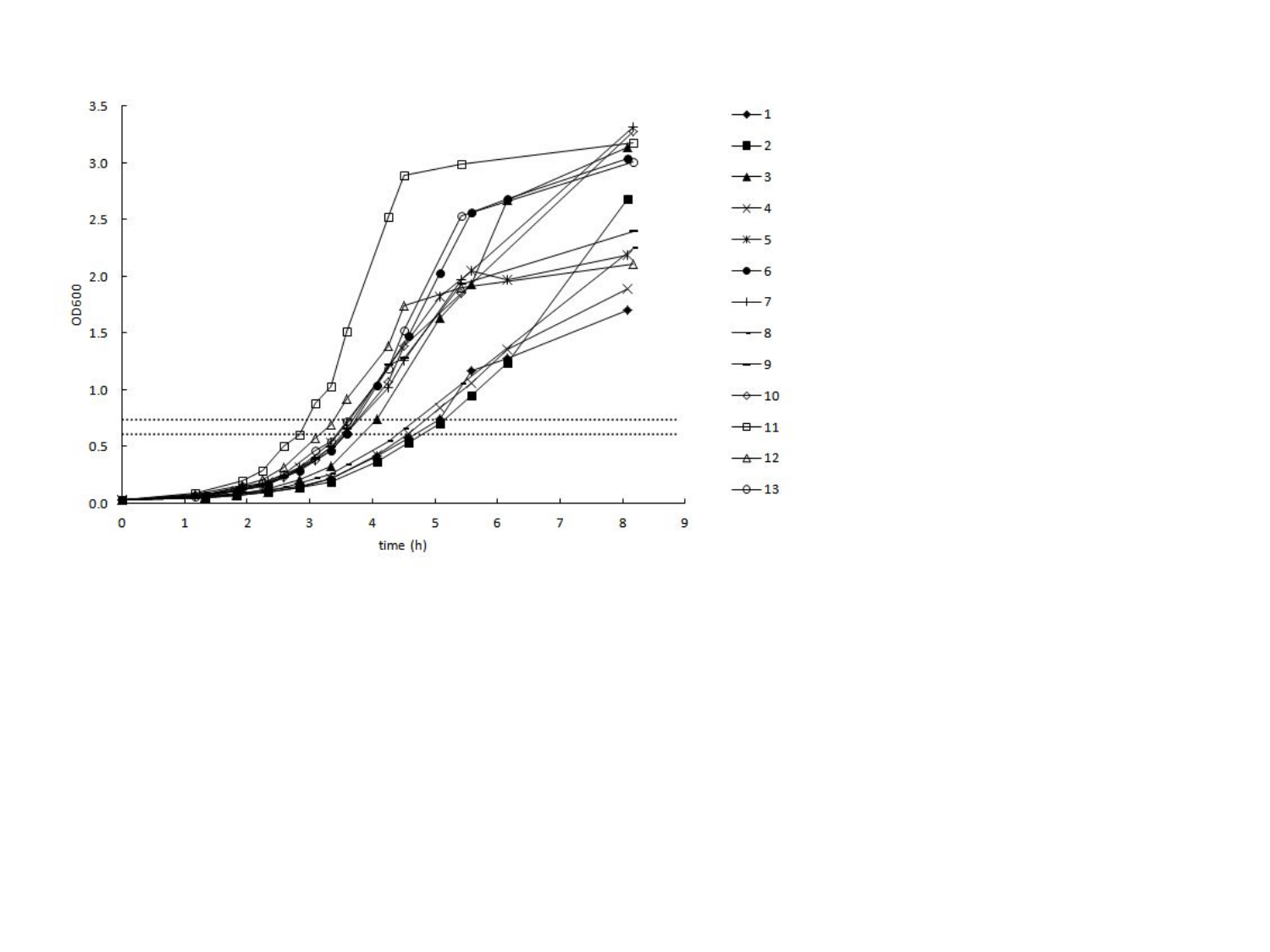

Supplement: Additional file 1: — Growth curves of MG1363 during various fermentations. Growth curves of strain MG1363 in fermentations as presented in Table 1. The data points between the dotted lines indicate the moment of harvesting cells for RNA isolation and stress survival assays. [file 12934_2014_148_MOESM1_ESM.pptx]

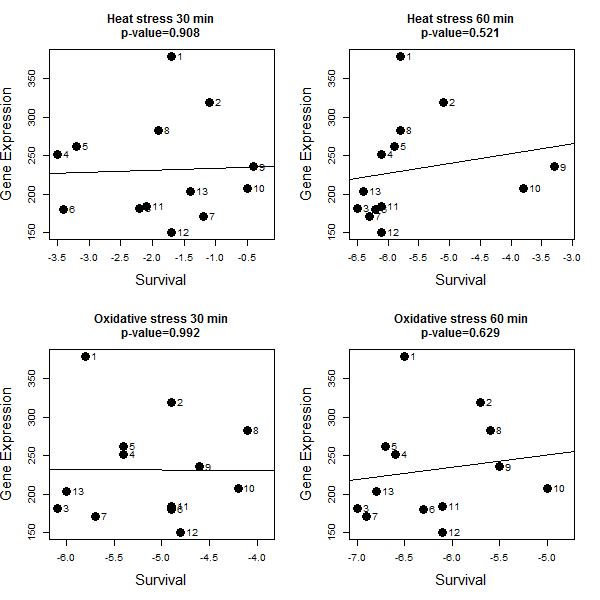

Supplement: Additional file 3: — Plots of gene expression and robustness levels. Expression levels of all genes plotted against survival after 30 and 60 minutes heat and oxidative stress (A: genes llmg_0001 to llmg_1229, B: genes llmg_1230 to llmg_2563). Survival is expressed as the difference of log CFU/ml after stress and before stress. Numbers indicate fermentations as presented in Table 1. P-values above the plots indicate significance of correlation (assessed by a linear model). [file 12934_2014_148_MOESM3_ESM.zip › Additional File 3A/llmg_0001_real_dat.png]

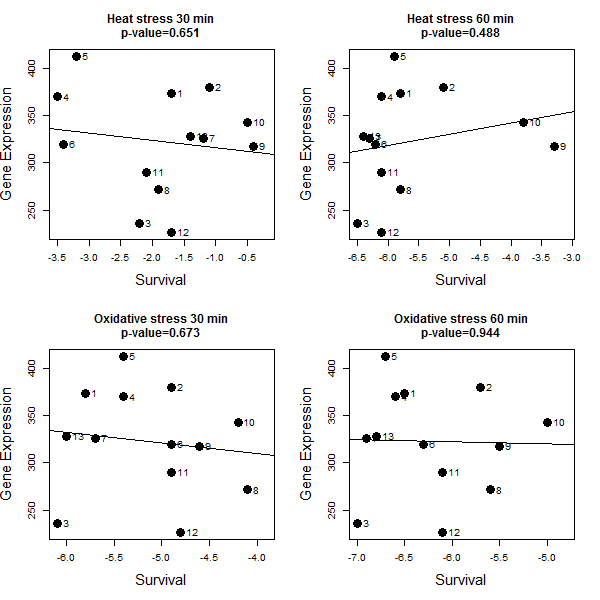

Supplement: Additional file 3: — Plots of gene expression and robustness levels. Expression levels of all genes plotted against survival after 30 and 60 minutes heat and oxidative stress (A: genes llmg_0001 to llmg_1229, B: genes llmg_1230 to llmg_2563). Survival is expressed as the difference of log CFU/ml after stress and before stress. Numbers indicate fermentations as presented in Table 1. P-values above the plots indicate significance of correlation (assessed by a linear model). [file 12934_2014_148_MOESM3_ESM.zip › Additional File 3A/llmg_0002_real_dat.png]

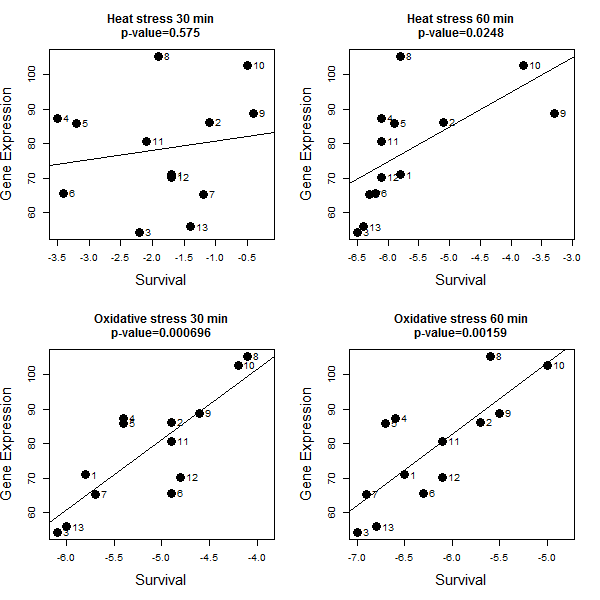

Supplement: Additional file 3: — Plots of gene expression and robustness levels. Expression levels of all genes plotted against survival after 30 and 60 minutes heat and oxidative stress (A: genes llmg_0001 to llmg_1229, B: genes llmg_1230 to llmg_2563). Survival is expressed as the difference of log CFU/ml after stress and before stress. Numbers indicate fermentations as presented in Table 1. P-values above the plots indicate significance of correlation (assessed by a linear model). [file 12934_2014_148_MOESM3_ESM.zip › Additional File 3A/llmg_0003_real_dat.png]

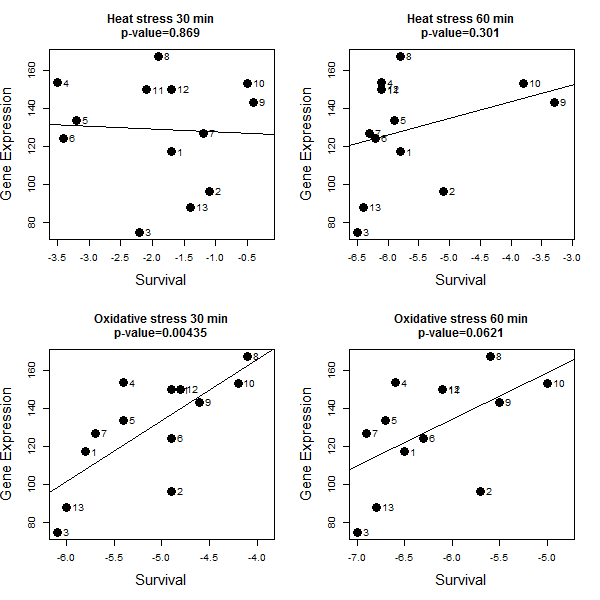

Supplement: Additional file 3: — Plots of gene expression and robustness levels. Expression levels of all genes plotted against survival after 30 and 60 minutes heat and oxidative stress (A: genes llmg_0001 to llmg_1229, B: genes llmg_1230 to llmg_2563). Survival is expressed as the difference of log CFU/ml after stress and before stress. Numbers indicate fermentations as presented in Table 1. P-values above the plots indicate significance of correlation (assessed by a linear model). [file 12934_2014_148_MOESM3_ESM.zip › Additional File 3A/llmg_0004_real_dat.png]

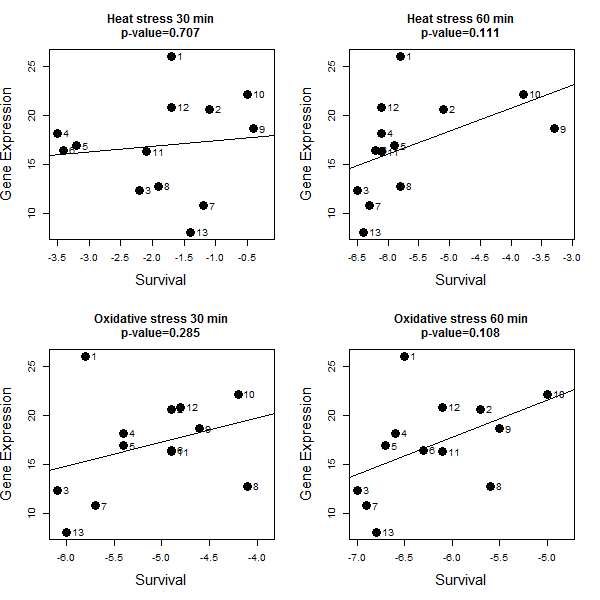

Supplement: Additional file 3: — Plots of gene expression and robustness levels. Expression levels of all genes plotted against survival after 30 and 60 minutes heat and oxidative stress (A: genes llmg_0001 to llmg_1229, B: genes llmg_1230 to llmg_2563). Survival is expressed as the difference of log CFU/ml after stress and before stress. Numbers indicate fermentations as presented in Table 1. P-values above the plots indicate significance of correlation (assessed by a linear model). [file 12934_2014_148_MOESM3_ESM.zip › Additional File 3A/llmg_0005_real_dat.png]

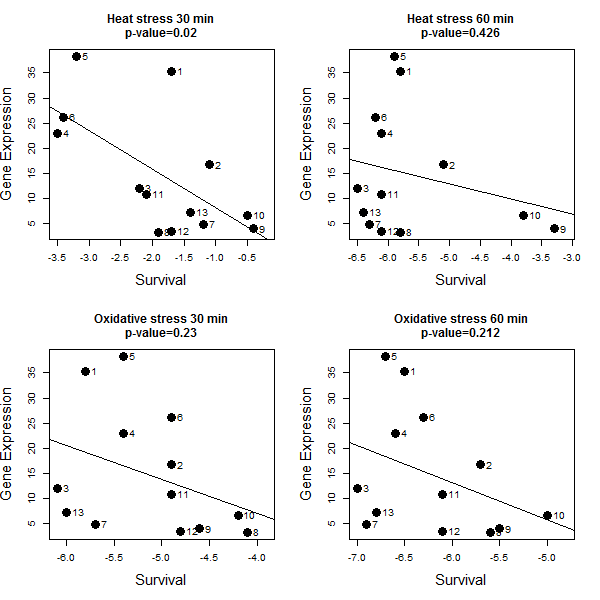

Supplement: Additional file 3: — Plots of gene expression and robustness levels. Expression levels of all genes plotted against survival after 30 and 60 minutes heat and oxidative stress (A: genes llmg_0001 to llmg_1229, B: genes llmg_1230 to llmg_2563). Survival is expressed as the difference of log CFU/ml after stress and before stress. Numbers indicate fermentations as presented in Table 1. P-values above the plots indicate significance of correlation (assessed by a linear model). [file 12934_2014_148_MOESM3_ESM.zip › Additional File 3A/llmg_0006_real_dat.png]

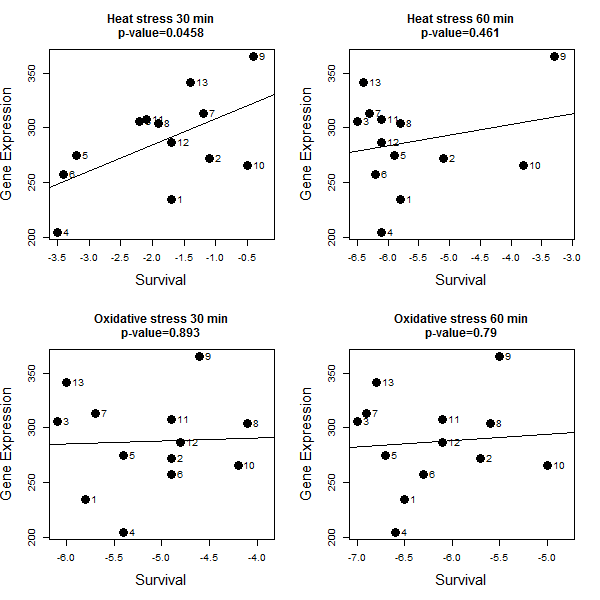

Supplement: Additional file 3: — Plots of gene expression and robustness levels. Expression levels of all genes plotted against survival after 30 and 60 minutes heat and oxidative stress (A: genes llmg_0001 to llmg_1229, B: genes llmg_1230 to llmg_2563). Survival is expressed as the difference of log CFU/ml after stress and before stress. Numbers indicate fermentations as presented in Table 1. P-values above the plots indicate significance of correlation (assessed by a linear model). [file 12934_2014_148_MOESM3_ESM.zip › Additional File 3A/llmg_0007_real_dat.png]

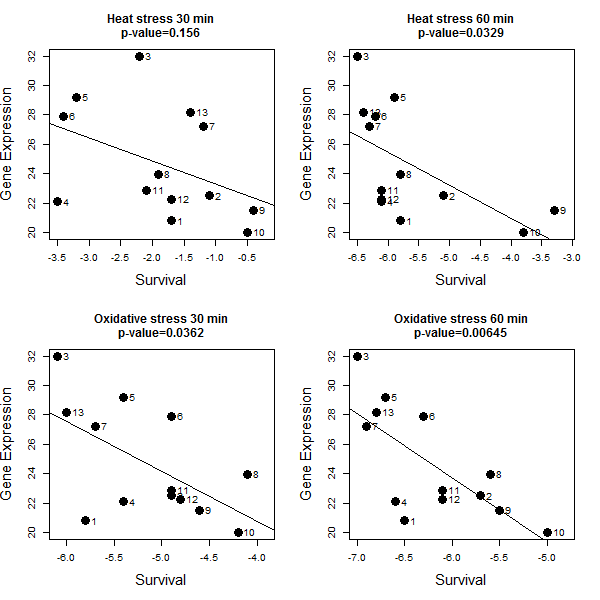

Supplement: Additional file 3: — Plots of gene expression and robustness levels. Expression levels of all genes plotted against survival after 30 and 60 minutes heat and oxidative stress (A: genes llmg_0001 to llmg_1229, B: genes llmg_1230 to llmg_2563). Survival is expressed as the difference of log CFU/ml after stress and before stress. Numbers indicate fermentations as presented in Table 1. P-values above the plots indicate significance of correlation (assessed by a linear model). [file 12934_2014_148_MOESM3_ESM.zip › Additional File 3A/llmg_0008_real_dat.png]

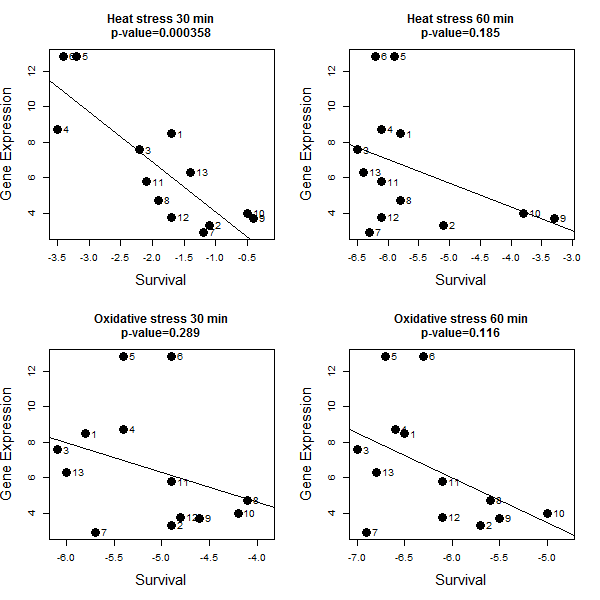

Supplement: Additional file 3: — Plots of gene expression and robustness levels. Expression levels of all genes plotted against survival after 30 and 60 minutes heat and oxidative stress (A: genes llmg_0001 to llmg_1229, B: genes llmg_1230 to llmg_2563). Survival is expressed as the difference of log CFU/ml after stress and before stress. Numbers indicate fermentations as presented in Table 1. P-values above the plots indicate significance of correlation (assessed by a linear model). [file 12934_2014_148_MOESM3_ESM.zip › Additional File 3A/llmg_0009_real_dat.png]

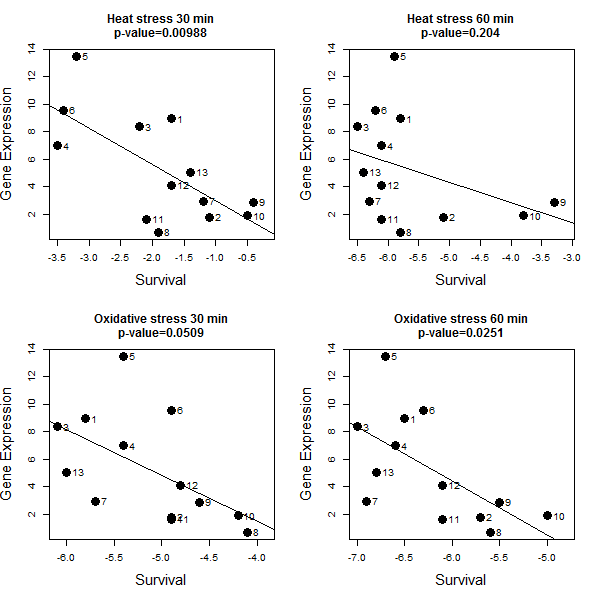

Supplement: Additional file 3: — Plots of gene expression and robustness levels. Expression levels of all genes plotted against survival after 30 and 60 minutes heat and oxidative stress (A: genes llmg_0001 to llmg_1229, B: genes llmg_1230 to llmg_2563). Survival is expressed as the difference of log CFU/ml after stress and before stress. Numbers indicate fermentations as presented in Table 1. P-values above the plots indicate significance of correlation (assessed by a linear model). [file 12934_2014_148_MOESM3_ESM.zip › Additional File 3A/llmg_0010_real_dat.png]

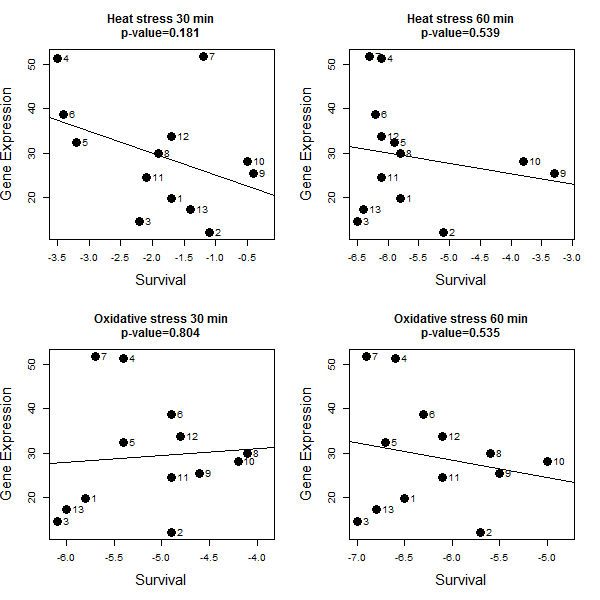

Supplement: Additional file 3: — Plots of gene expression and robustness levels. Expression levels of all genes plotted against survival after 30 and 60 minutes heat and oxidative stress (A: genes llmg_0001 to llmg_1229, B: genes llmg_1230 to llmg_2563). Survival is expressed as the difference of log CFU/ml after stress and before stress. Numbers indicate fermentations as presented in Table 1. P-values above the plots indicate significance of correlation (assessed by a linear model). [file 12934_2014_148_MOESM3_ESM.zip › Additional File 3A/llmg_0011_real_dat.png]

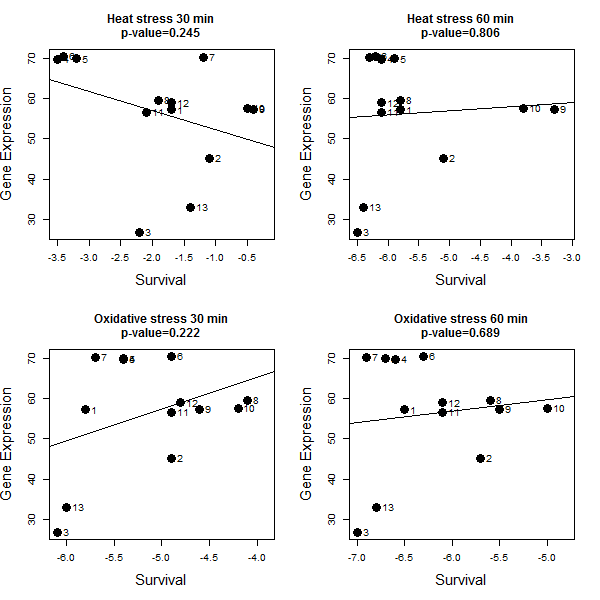

Supplement: Additional file 3: — Plots of gene expression and robustness levels. Expression levels of all genes plotted against survival after 30 and 60 minutes heat and oxidative stress (A: genes llmg_0001 to llmg_1229, B: genes llmg_1230 to llmg_2563). Survival is expressed as the difference of log CFU/ml after stress and before stress. Numbers indicate fermentations as presented in Table 1. P-values above the plots indicate significance of correlation (assessed by a linear model). [file 12934_2014_148_MOESM3_ESM.zip › Additional File 3A/llmg_0012_real_dat.png]

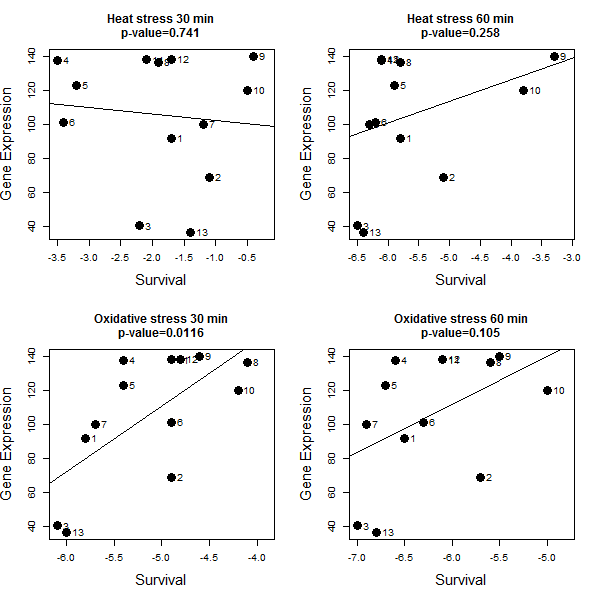

Supplement: Additional file 3: — Plots of gene expression and robustness levels. Expression levels of all genes plotted against survival after 30 and 60 minutes heat and oxidative stress (A: genes llmg_0001 to llmg_1229, B: genes llmg_1230 to llmg_2563). Survival is expressed as the difference of log CFU/ml after stress and before stress. Numbers indicate fermentations as presented in Table 1. P-values above the plots indicate significance of correlation (assessed by a linear model). [file 12934_2014_148_MOESM3_ESM.zip › Additional File 3A/llmg_0013_real_dat.png]

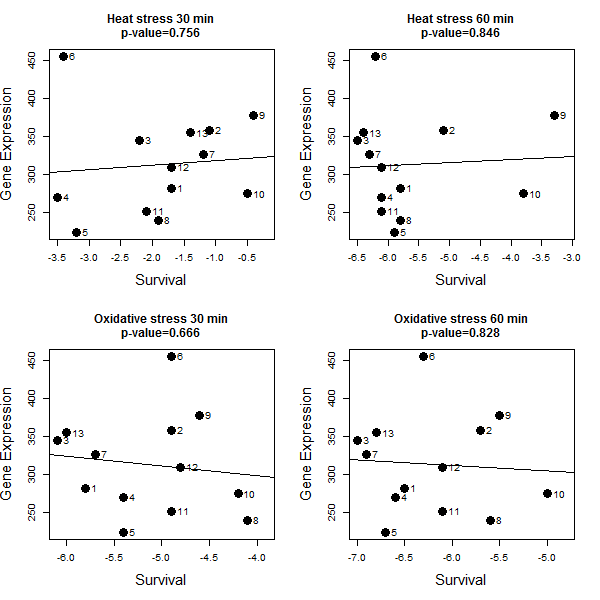

Supplement: Additional file 3: — Plots of gene expression and robustness levels. Expression levels of all genes plotted against survival after 30 and 60 minutes heat and oxidative stress (A: genes llmg_0001 to llmg_1229, B: genes llmg_1230 to llmg_2563). Survival is expressed as the difference of log CFU/ml after stress and before stress. Numbers indicate fermentations as presented in Table 1. P-values above the plots indicate significance of correlation (assessed by a linear model). [file 12934_2014_148_MOESM3_ESM.zip › Additional File 3A/llmg_0015_real_dat.png]

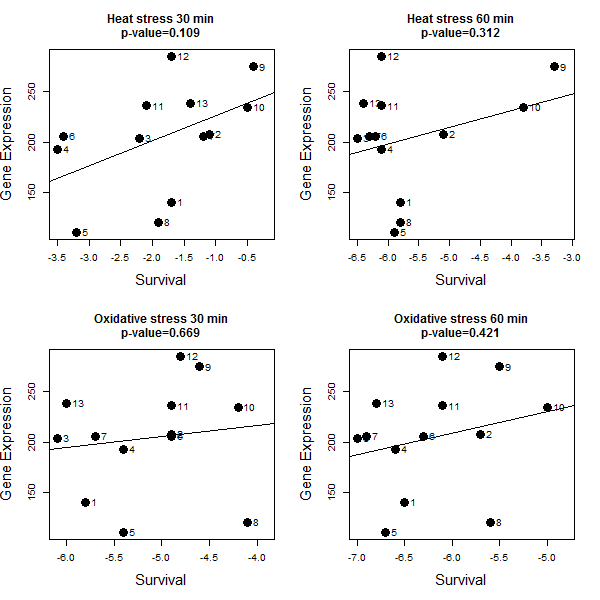

Supplement: Additional file 3: — Plots of gene expression and robustness levels. Expression levels of all genes plotted against survival after 30 and 60 minutes heat and oxidative stress (A: genes llmg_0001 to llmg_1229, B: genes llmg_1230 to llmg_2563). Survival is expressed as the difference of log CFU/ml after stress and before stress. Numbers indicate fermentations as presented in Table 1. P-values above the plots indicate significance of correlation (assessed by a linear model). [file 12934_2014_148_MOESM3_ESM.zip › Additional File 3A/llmg_0016_real_dat.png]

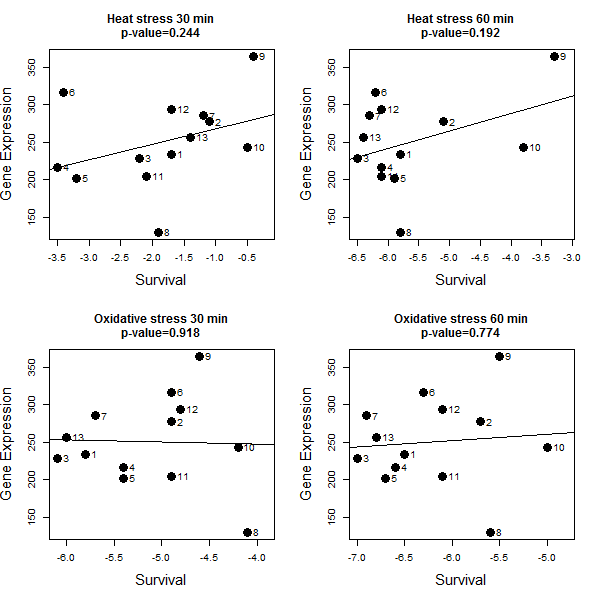

Supplement: Additional file 3: — Plots of gene expression and robustness levels. Expression levels of all genes plotted against survival after 30 and 60 minutes heat and oxidative stress (A: genes llmg_0001 to llmg_1229, B: genes llmg_1230 to llmg_2563). Survival is expressed as the difference of log CFU/ml after stress and before stress. Numbers indicate fermentations as presented in Table 1. P-values above the plots indicate significance of correlation (assessed by a linear model). [file 12934_2014_148_MOESM3_ESM.zip › Additional File 3A/llmg_0017_real_dat.png]

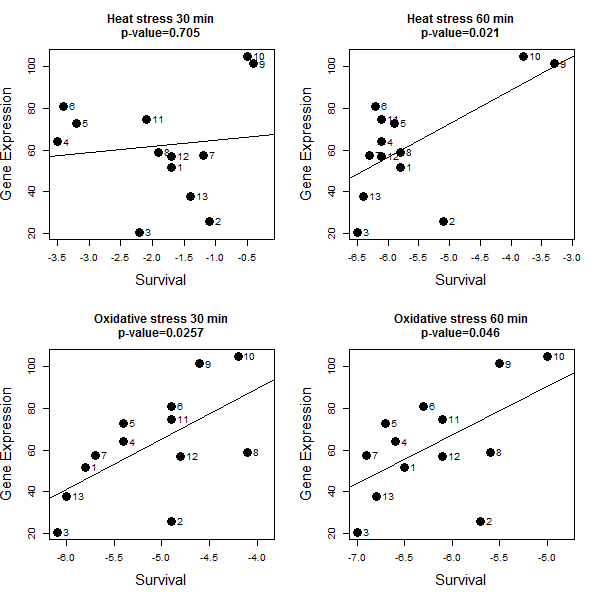

Supplement: Additional file 3: — Plots of gene expression and robustness levels. Expression levels of all genes plotted against survival after 30 and 60 minutes heat and oxidative stress (A: genes llmg_0001 to llmg_1229, B: genes llmg_1230 to llmg_2563). Survival is expressed as the difference of log CFU/ml after stress and before stress. Numbers indicate fermentations as presented in Table 1. P-values above the plots indicate significance of correlation (assessed by a linear model). [file 12934_2014_148_MOESM3_ESM.zip › Additional File 3A/llmg_0018_real_dat.png]

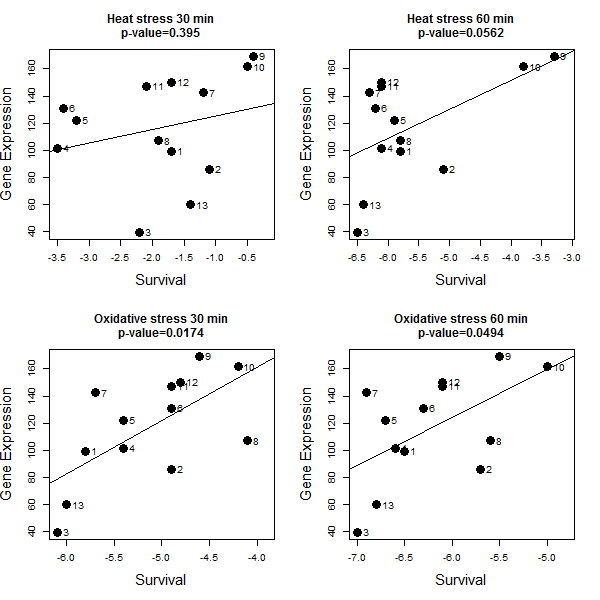

Supplement: Additional file 3: — Plots of gene expression and robustness levels. Expression levels of all genes plotted against survival after 30 and 60 minutes heat and oxidative stress (A: genes llmg_0001 to llmg_1229, B: genes llmg_1230 to llmg_2563). Survival is expressed as the difference of log CFU/ml after stress and before stress. Numbers indicate fermentations as presented in Table 1. P-values above the plots indicate significance of correlation (assessed by a linear model). [file 12934_2014_148_MOESM3_ESM.zip › Additional File 3A/llmg_0019_real_dat.png]

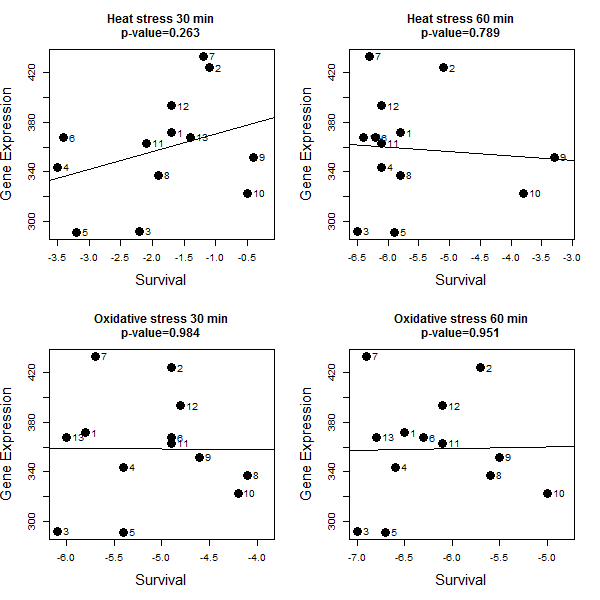

Supplement: Additional file 3: — Plots of gene expression and robustness levels. Expression levels of all genes plotted against survival after 30 and 60 minutes heat and oxidative stress (A: genes llmg_0001 to llmg_1229, B: genes llmg_1230 to llmg_2563). Survival is expressed as the difference of log CFU/ml after stress and before stress. Numbers indicate fermentations as presented in Table 1. P-values above the plots indicate significance of correlation (assessed by a linear model). [file 12934_2014_148_MOESM3_ESM.zip › Additional File 3A/llmg_0020_real_dat.png]

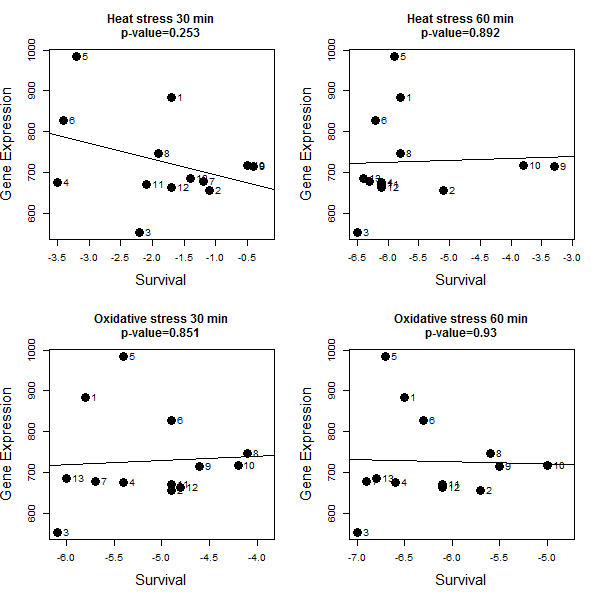

Supplement: Additional file 3: — Plots of gene expression and robustness levels. Expression levels of all genes plotted against survival after 30 and 60 minutes heat and oxidative stress (A: genes llmg_0001 to llmg_1229, B: genes llmg_1230 to llmg_2563). Survival is expressed as the difference of log CFU/ml after stress and before stress. Numbers indicate fermentations as presented in Table 1. P-values above the plots indicate significance of correlation (assessed by a linear model). [file 12934_2014_148_MOESM3_ESM.zip › Additional File 3A/llmg_0021_real_dat.png]

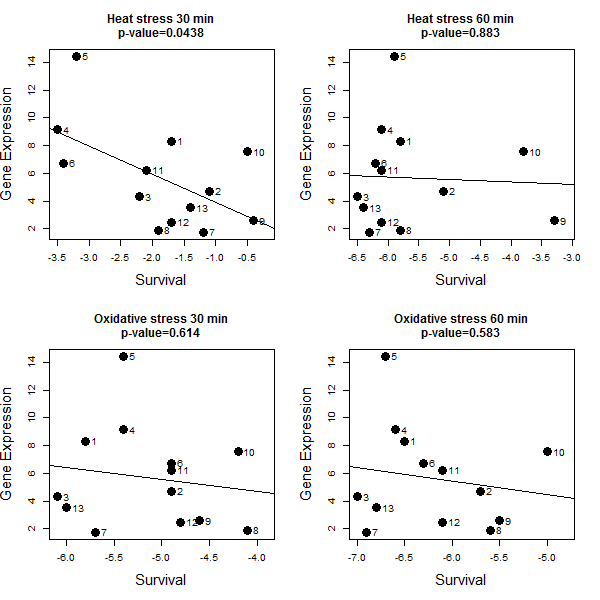

Supplement: Additional file 3: — Plots of gene expression and robustness levels. Expression levels of all genes plotted against survival after 30 and 60 minutes heat and oxidative stress (A: genes llmg_0001 to llmg_1229, B: genes llmg_1230 to llmg_2563). Survival is expressed as the difference of log CFU/ml after stress and before stress. Numbers indicate fermentations as presented in Table 1. P-values above the plots indicate significance of correlation (assessed by a linear model). [file 12934_2014_148_MOESM3_ESM.zip › Additional File 3A/llmg_0022_real_dat.png]

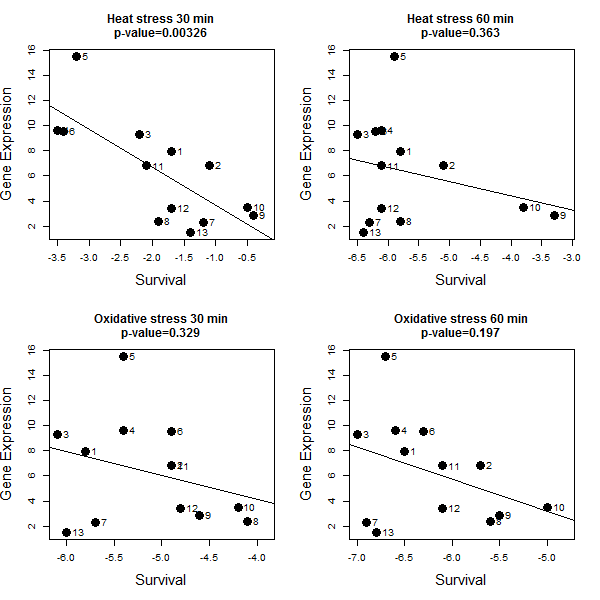

Supplement: Additional file 3: — Plots of gene expression and robustness levels. Expression levels of all genes plotted against survival after 30 and 60 minutes heat and oxidative stress (A: genes llmg_0001 to llmg_1229, B: genes llmg_1230 to llmg_2563). Survival is expressed as the difference of log CFU/ml after stress and before stress. Numbers indicate fermentations as presented in Table 1. P-values above the plots indicate significance of correlation (assessed by a linear model). [file 12934_2014_148_MOESM3_ESM.zip › Additional File 3A/llmg_0023_real_dat.png]

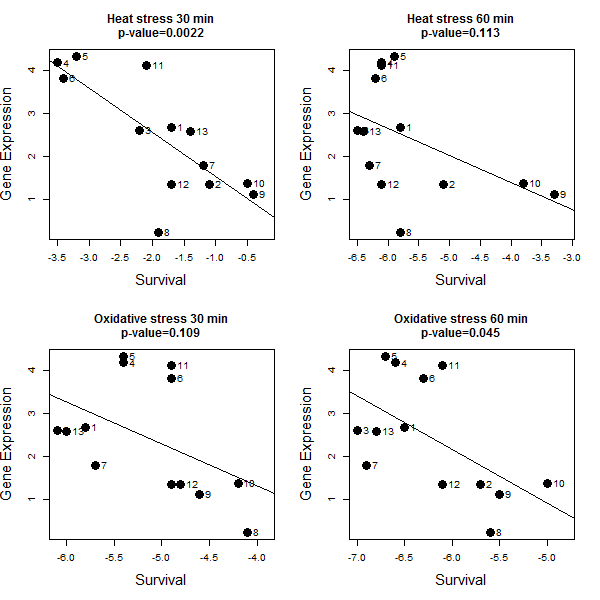

Supplement: Additional file 3: — Plots of gene expression and robustness levels. Expression levels of all genes plotted against survival after 30 and 60 minutes heat and oxidative stress (A: genes llmg_0001 to llmg_1229, B: genes llmg_1230 to llmg_2563). Survival is expressed as the difference of log CFU/ml after stress and before stress. Numbers indicate fermentations as presented in Table 1. P-values above the plots indicate significance of correlation (assessed by a linear model). [file 12934_2014_148_MOESM3_ESM.zip › Additional File 3A/llmg_0024_real_dat.png]

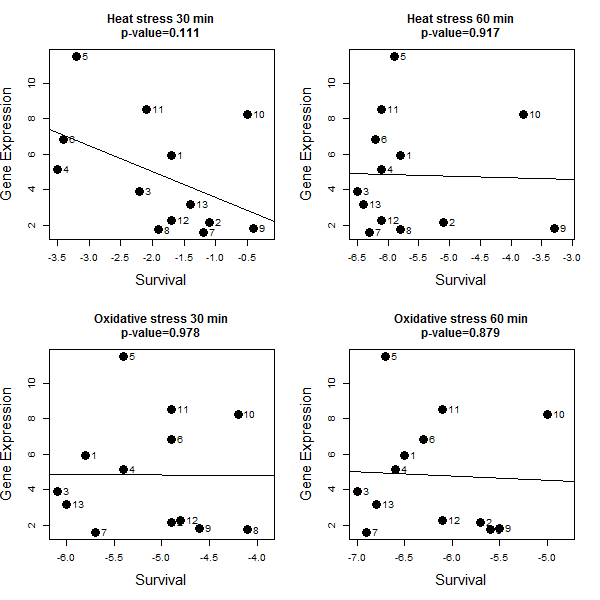

Supplement: Additional file 3: — Plots of gene expression and robustness levels. Expression levels of all genes plotted against survival after 30 and 60 minutes heat and oxidative stress (A: genes llmg_0001 to llmg_1229, B: genes llmg_1230 to llmg_2563). Survival is expressed as the difference of log CFU/ml after stress and before stress. Numbers indicate fermentations as presented in Table 1. P-values above the plots indicate significance of correlation (assessed by a linear model). [file 12934_2014_148_MOESM3_ESM.zip › Additional File 3A/llmg_0025_real_dat.png]

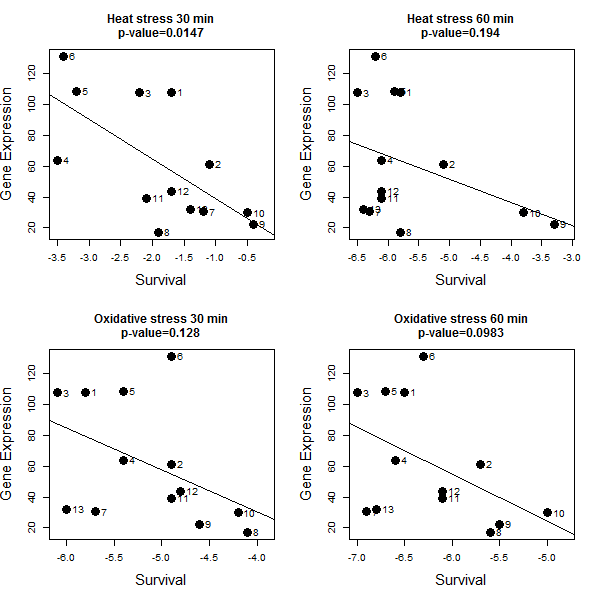

Supplement: Additional file 3: — Plots of gene expression and robustness levels. Expression levels of all genes plotted against survival after 30 and 60 minutes heat and oxidative stress (A: genes llmg_0001 to llmg_1229, B: genes llmg_1230 to llmg_2563). Survival is expressed as the difference of log CFU/ml after stress and before stress. Numbers indicate fermentations as presented in Table 1. P-values above the plots indicate significance of correlation (assessed by a linear model). [file 12934_2014_148_MOESM3_ESM.zip › Additional File 3A/llmg_0026_real_dat.png]

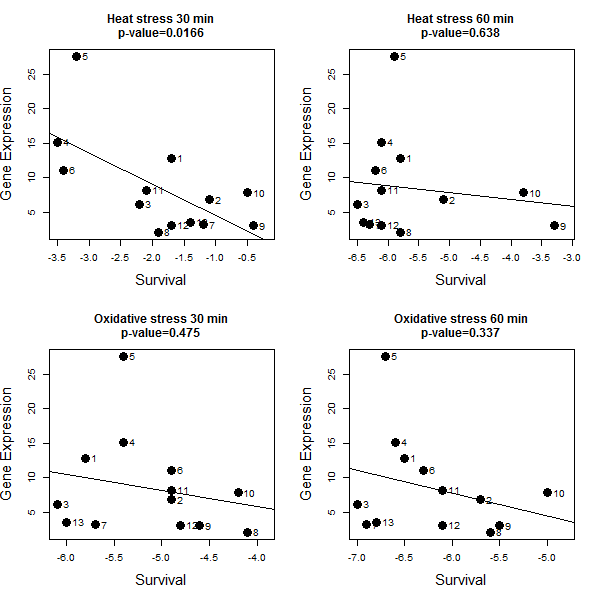

Supplement: Additional file 3: — Plots of gene expression and robustness levels. Expression levels of all genes plotted against survival after 30 and 60 minutes heat and oxidative stress (A: genes llmg_0001 to llmg_1229, B: genes llmg_1230 to llmg_2563). Survival is expressed as the difference of log CFU/ml after stress and before stress. Numbers indicate fermentations as presented in Table 1. P-values above the plots indicate significance of correlation (assessed by a linear model). [file 12934_2014_148_MOESM3_ESM.zip › Additional File 3A/llmg_0027_real_dat.png]

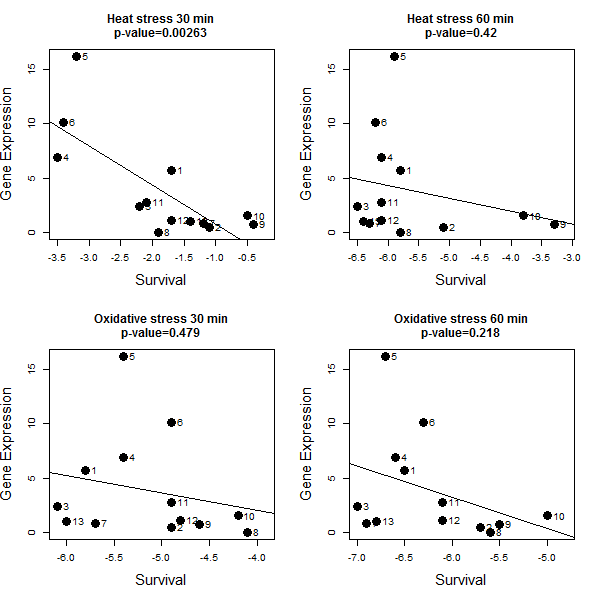

Supplement: Additional file 3: — Plots of gene expression and robustness levels. Expression levels of all genes plotted against survival after 30 and 60 minutes heat and oxidative stress (A: genes llmg_0001 to llmg_1229, B: genes llmg_1230 to llmg_2563). Survival is expressed as the difference of log CFU/ml after stress and before stress. Numbers indicate fermentations as presented in Table 1. P-values above the plots indicate significance of correlation (assessed by a linear model). [file 12934_2014_148_MOESM3_ESM.zip › Additional File 3A/llmg_0028_real_dat.png]

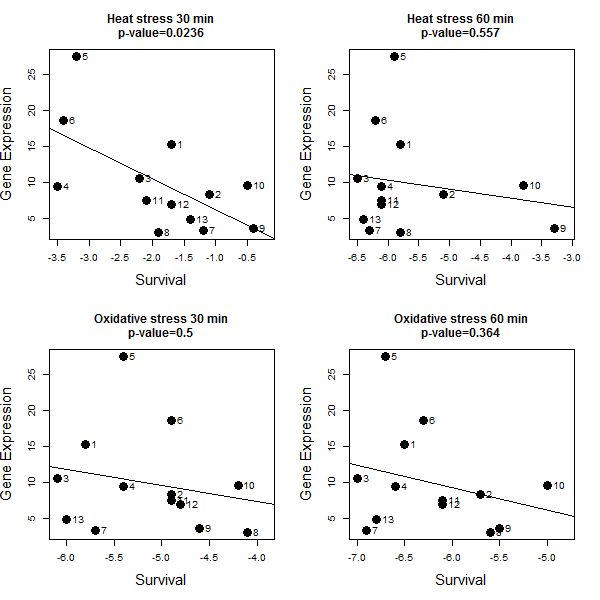

Supplement: Additional file 3: — Plots of gene expression and robustness levels. Expression levels of all genes plotted against survival after 30 and 60 minutes heat and oxidative stress (A: genes llmg_0001 to llmg_1229, B: genes llmg_1230 to llmg_2563). Survival is expressed as the difference of log CFU/ml after stress and before stress. Numbers indicate fermentations as presented in Table 1. P-values above the plots indicate significance of correlation (assessed by a linear model). [file 12934_2014_148_MOESM3_ESM.zip › Additional File 3A/llmg_0029_real_dat.png]

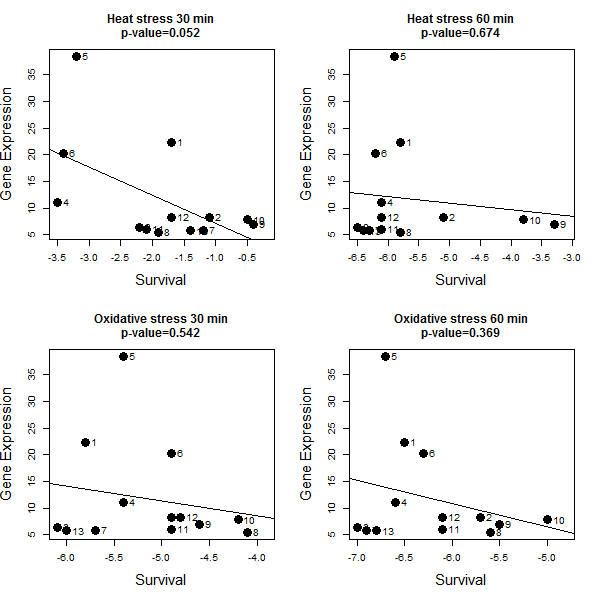

Supplement: Additional file 3: — Plots of gene expression and robustness levels. Expression levels of all genes plotted against survival after 30 and 60 minutes heat and oxidative stress (A: genes llmg_0001 to llmg_1229, B: genes llmg_1230 to llmg_2563). Survival is expressed as the difference of log CFU/ml after stress and before stress. Numbers indicate fermentations as presented in Table 1. P-values above the plots indicate significance of correlation (assessed by a linear model). [file 12934_2014_148_MOESM3_ESM.zip › Additional File 3A/llmg_0030_real_dat.png]

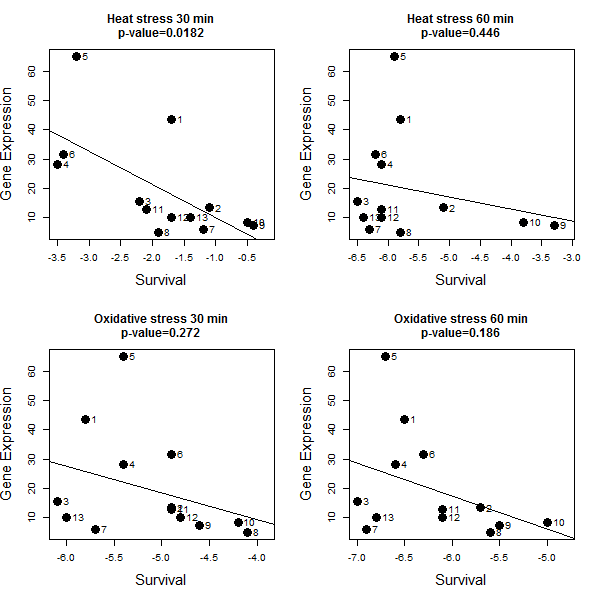

Supplement: Additional file 3: — Plots of gene expression and robustness levels. Expression levels of all genes plotted against survival after 30 and 60 minutes heat and oxidative stress (A: genes llmg_0001 to llmg_1229, B: genes llmg_1230 to llmg_2563). Survival is expressed as the difference of log CFU/ml after stress and before stress. Numbers indicate fermentations as presented in Table 1. P-values above the plots indicate significance of correlation (assessed by a linear model). [file 12934_2014_148_MOESM3_ESM.zip › Additional File 3A/llmg_0031_real_dat.png]

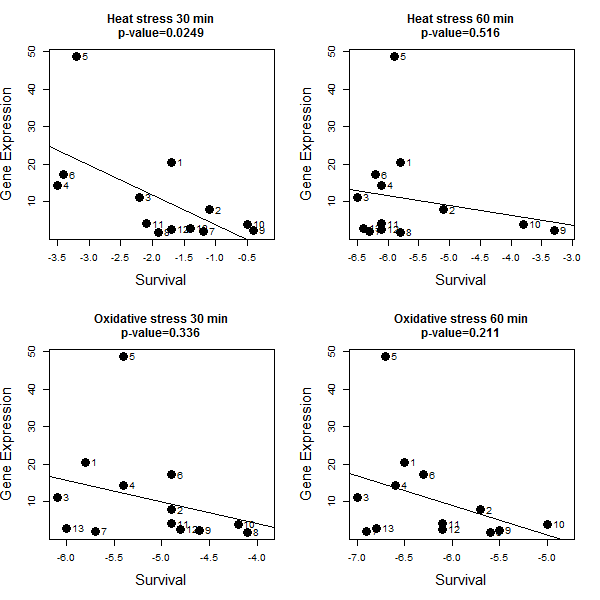

Supplement: Additional file 3: — Plots of gene expression and robustness levels. Expression levels of all genes plotted against survival after 30 and 60 minutes heat and oxidative stress (A: genes llmg_0001 to llmg_1229, B: genes llmg_1230 to llmg_2563). Survival is expressed as the difference of log CFU/ml after stress and before stress. Numbers indicate fermentations as presented in Table 1. P-values above the plots indicate significance of correlation (assessed by a linear model). [file 12934_2014_148_MOESM3_ESM.zip › Additional File 3A/llmg_0032_real_dat.png]

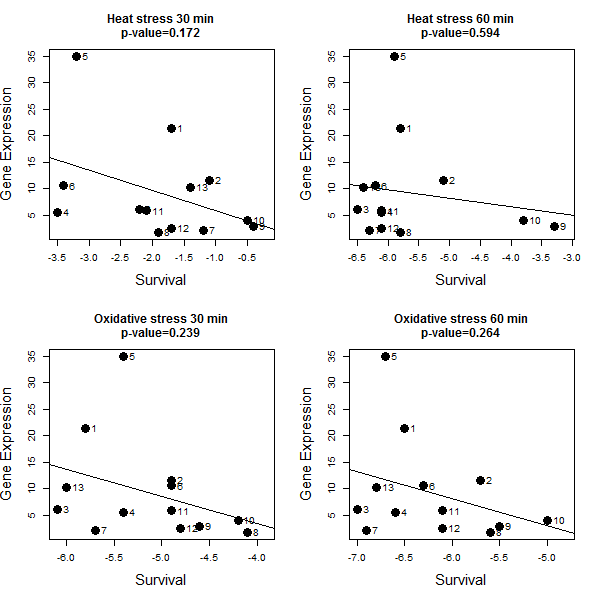

Supplement: Additional file 3: — Plots of gene expression and robustness levels. Expression levels of all genes plotted against survival after 30 and 60 minutes heat and oxidative stress (A: genes llmg_0001 to llmg_1229, B: genes llmg_1230 to llmg_2563). Survival is expressed as the difference of log CFU/ml after stress and before stress. Numbers indicate fermentations as presented in Table 1. P-values above the plots indicate significance of correlation (assessed by a linear model). [file 12934_2014_148_MOESM3_ESM.zip › Additional File 3A/llmg_0033_real_dat.png]

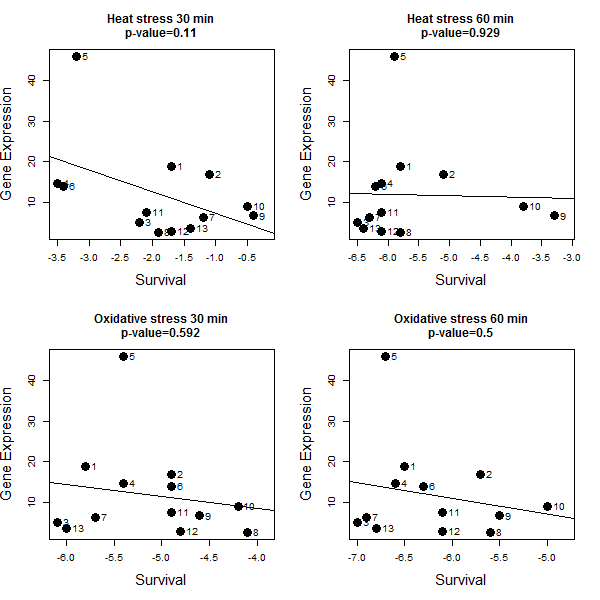

Supplement: Additional file 3: — Plots of gene expression and robustness levels. Expression levels of all genes plotted against survival after 30 and 60 minutes heat and oxidative stress (A: genes llmg_0001 to llmg_1229, B: genes llmg_1230 to llmg_2563). Survival is expressed as the difference of log CFU/ml after stress and before stress. Numbers indicate fermentations as presented in Table 1. P-values above the plots indicate significance of correlation (assessed by a linear model). [file 12934_2014_148_MOESM3_ESM.zip › Additional File 3A/llmg_0034_real_dat.png]

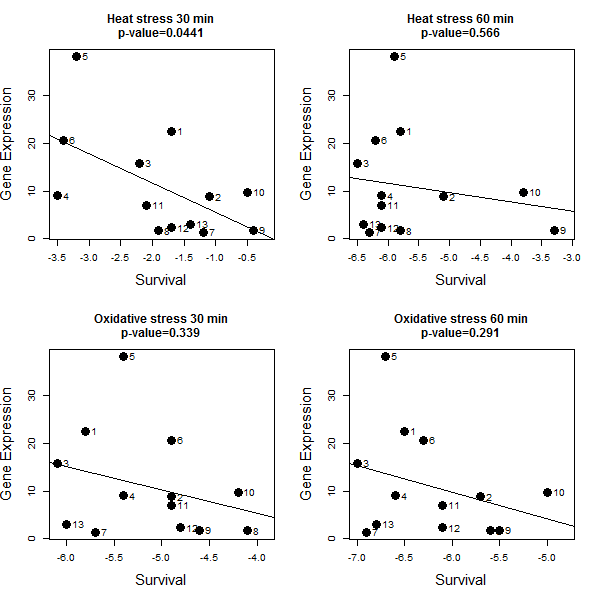

Supplement: Additional file 3: — Plots of gene expression and robustness levels. Expression levels of all genes plotted against survival after 30 and 60 minutes heat and oxidative stress (A: genes llmg_0001 to llmg_1229, B: genes llmg_1230 to llmg_2563). Survival is expressed as the difference of log CFU/ml after stress and before stress. Numbers indicate fermentations as presented in Table 1. P-values above the plots indicate significance of correlation (assessed by a linear model). [file 12934_2014_148_MOESM3_ESM.zip › Additional File 3A/llmg_0035_real_dat.png]

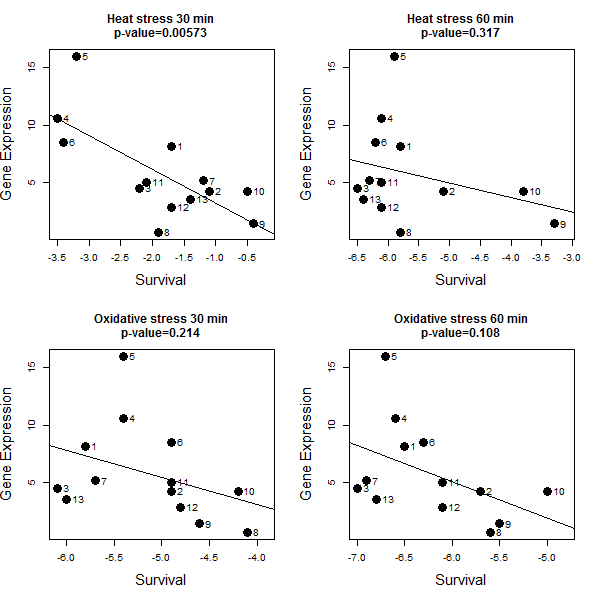

Supplement: Additional file 3: — Plots of gene expression and robustness levels. Expression levels of all genes plotted against survival after 30 and 60 minutes heat and oxidative stress (A: genes llmg_0001 to llmg_1229, B: genes llmg_1230 to llmg_2563). Survival is expressed as the difference of log CFU/ml after stress and before stress. Numbers indicate fermentations as presented in Table 1. P-values above the plots indicate significance of correlation (assessed by a linear model). [file 12934_2014_148_MOESM3_ESM.zip › Additional File 3A/llmg_0036_real_dat.png]

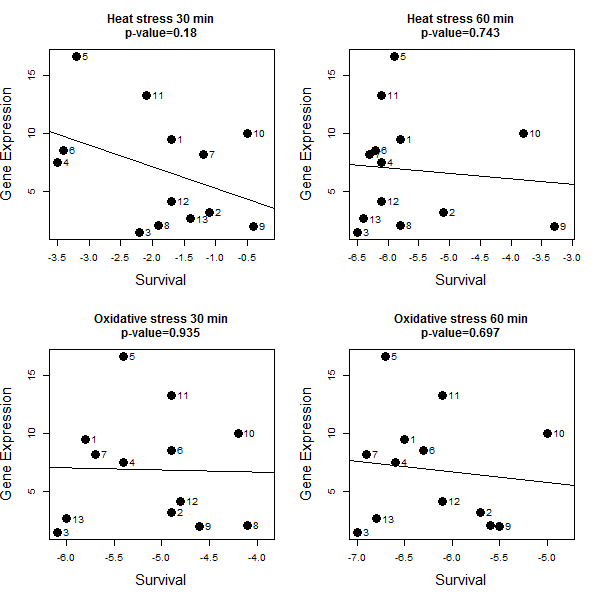

Supplement: Additional file 3: — Plots of gene expression and robustness levels. Expression levels of all genes plotted against survival after 30 and 60 minutes heat and oxidative stress (A: genes llmg_0001 to llmg_1229, B: genes llmg_1230 to llmg_2563). Survival is expressed as the difference of log CFU/ml after stress and before stress. Numbers indicate fermentations as presented in Table 1. P-values above the plots indicate significance of correlation (assessed by a linear model). [file 12934_2014_148_MOESM3_ESM.zip › Additional File 3A/llmg_0037_real_dat.png]

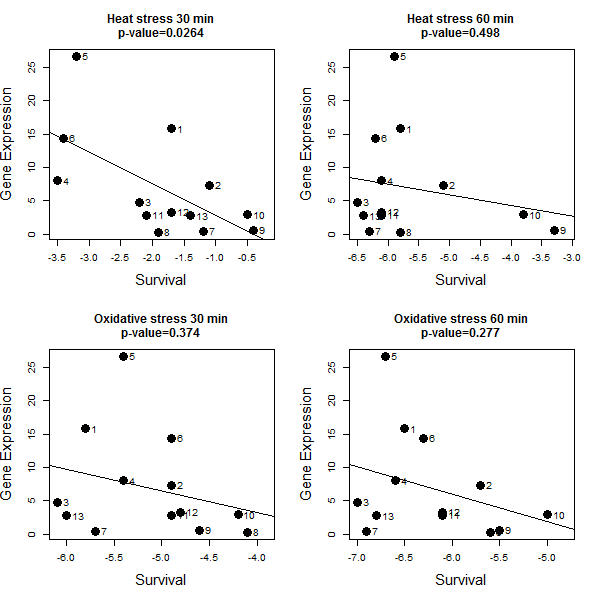

Supplement: Additional file 3: — Plots of gene expression and robustness levels. Expression levels of all genes plotted against survival after 30 and 60 minutes heat and oxidative stress (A: genes llmg_0001 to llmg_1229, B: genes llmg_1230 to llmg_2563). Survival is expressed as the difference of log CFU/ml after stress and before stress. Numbers indicate fermentations as presented in Table 1. P-values above the plots indicate significance of correlation (assessed by a linear model). [file 12934_2014_148_MOESM3_ESM.zip › Additional File 3A/llmg_0038_real_dat.png]

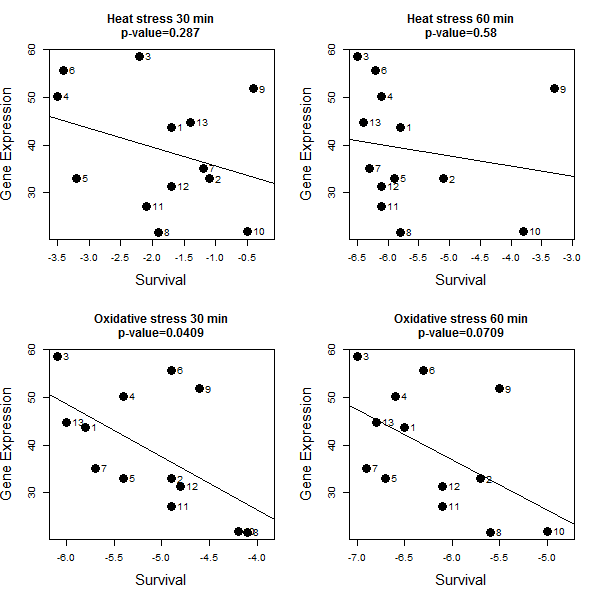

Supplement: Additional file 3: — Plots of gene expression and robustness levels. Expression levels of all genes plotted against survival after 30 and 60 minutes heat and oxidative stress (A: genes llmg_0001 to llmg_1229, B: genes llmg_1230 to llmg_2563). Survival is expressed as the difference of log CFU/ml after stress and before stress. Numbers indicate fermentations as presented in Table 1. P-values above the plots indicate significance of correlation (assessed by a linear model). [file 12934_2014_148_MOESM3_ESM.zip › Additional File 3A/llmg_0039_real_dat.png]

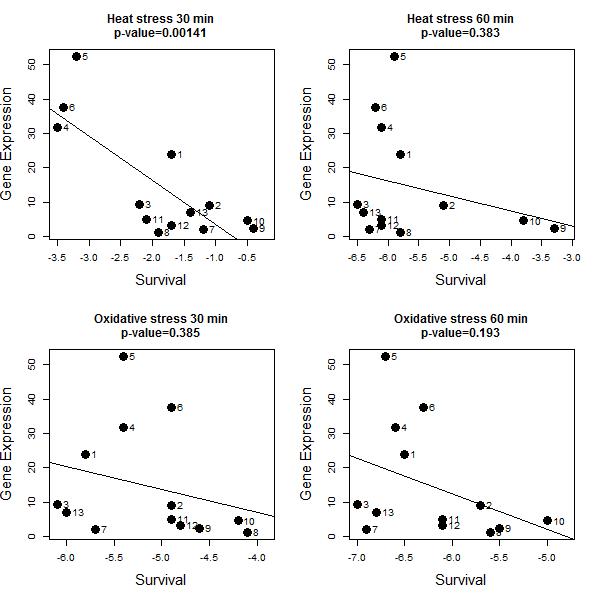

Supplement: Additional file 3: — Plots of gene expression and robustness levels. Expression levels of all genes plotted against survival after 30 and 60 minutes heat and oxidative stress (A: genes llmg_0001 to llmg_1229, B: genes llmg_1230 to llmg_2563). Survival is expressed as the difference of log CFU/ml after stress and before stress. Numbers indicate fermentations as presented in Table 1. P-values above the plots indicate significance of correlation (assessed by a linear model). [file 12934_2014_148_MOESM3_ESM.zip › Additional File 3A/llmg_0040_real_dat.png]

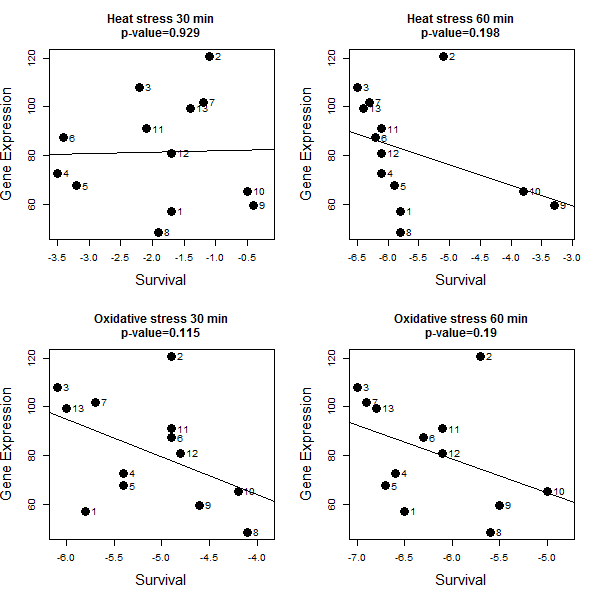

Supplement: Additional file 3: — Plots of gene expression and robustness levels. Expression levels of all genes plotted against survival after 30 and 60 minutes heat and oxidative stress (A: genes llmg_0001 to llmg_1229, B: genes llmg_1230 to llmg_2563). Survival is expressed as the difference of log CFU/ml after stress and before stress. Numbers indicate fermentations as presented in Table 1. P-values above the plots indicate significance of correlation (assessed by a linear model). [file 12934_2014_148_MOESM3_ESM.zip › Additional File 3A/llmg_0041_real_dat.png]

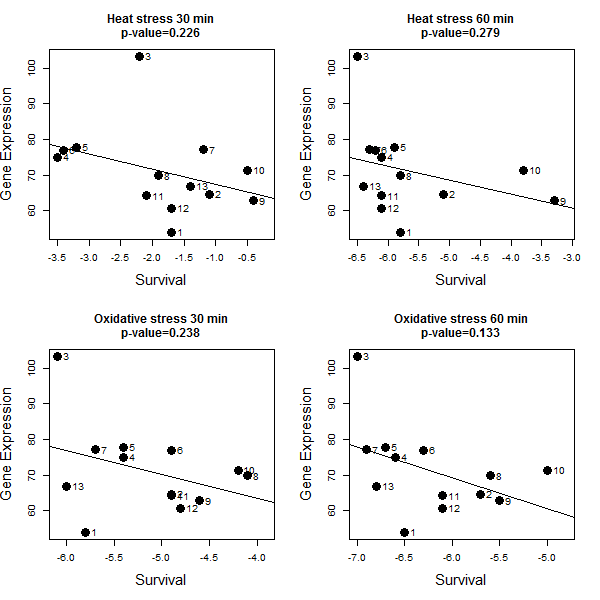

Supplement: Additional file 3: — Plots of gene expression and robustness levels. Expression levels of all genes plotted against survival after 30 and 60 minutes heat and oxidative stress (A: genes llmg_0001 to llmg_1229, B: genes llmg_1230 to llmg_2563). Survival is expressed as the difference of log CFU/ml after stress and before stress. Numbers indicate fermentations as presented in Table 1. P-values above the plots indicate significance of correlation (assessed by a linear model). [file 12934_2014_148_MOESM3_ESM.zip › Additional File 3A/llmg_0042_real_dat.png]

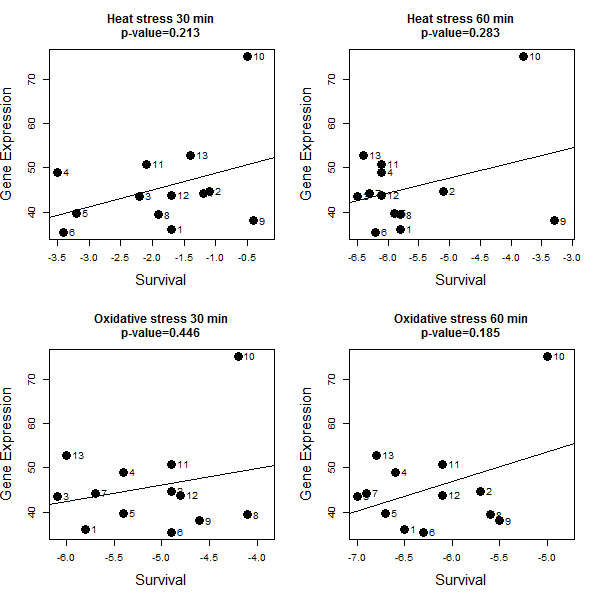

Supplement: Additional file 3: — Plots of gene expression and robustness levels. Expression levels of all genes plotted against survival after 30 and 60 minutes heat and oxidative stress (A: genes llmg_0001 to llmg_1229, B: genes llmg_1230 to llmg_2563). Survival is expressed as the difference of log CFU/ml after stress and before stress. Numbers indicate fermentations as presented in Table 1. P-values above the plots indicate significance of correlation (assessed by a linear model). [file 12934_2014_148_MOESM3_ESM.zip › Additional File 3A/llmg_0043_real_dat.png]

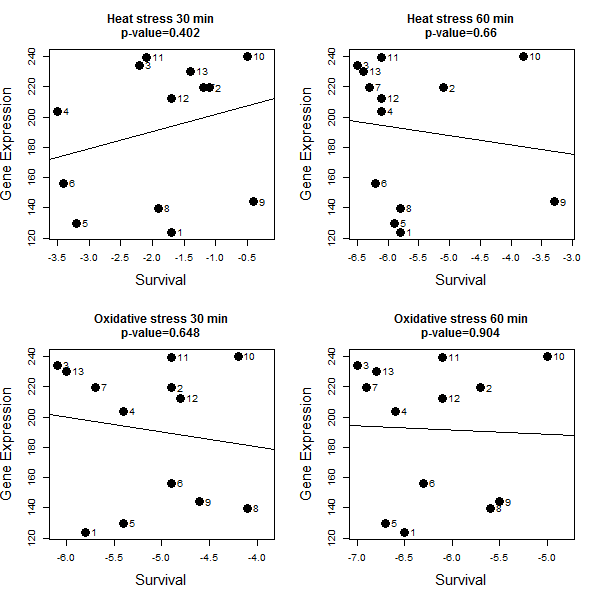

Supplement: Additional file 3: — Plots of gene expression and robustness levels. Expression levels of all genes plotted against survival after 30 and 60 minutes heat and oxidative stress (A: genes llmg_0001 to llmg_1229, B: genes llmg_1230 to llmg_2563). Survival is expressed as the difference of log CFU/ml after stress and before stress. Numbers indicate fermentations as presented in Table 1. P-values above the plots indicate significance of correlation (assessed by a linear model). [file 12934_2014_148_MOESM3_ESM.zip › Additional File 3A/llmg_0044_real_dat.png]

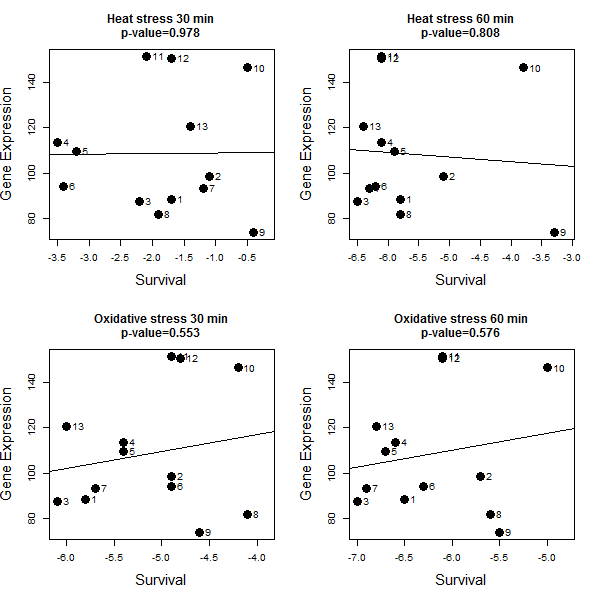

Supplement: Additional file 3: — Plots of gene expression and robustness levels. Expression levels of all genes plotted against survival after 30 and 60 minutes heat and oxidative stress (A: genes llmg_0001 to llmg_1229, B: genes llmg_1230 to llmg_2563). Survival is expressed as the difference of log CFU/ml after stress and before stress. Numbers indicate fermentations as presented in Table 1. P-values above the plots indicate significance of correlation (assessed by a linear model). [file 12934_2014_148_MOESM3_ESM.zip › Additional File 3A/llmg_0045_real_dat.png]

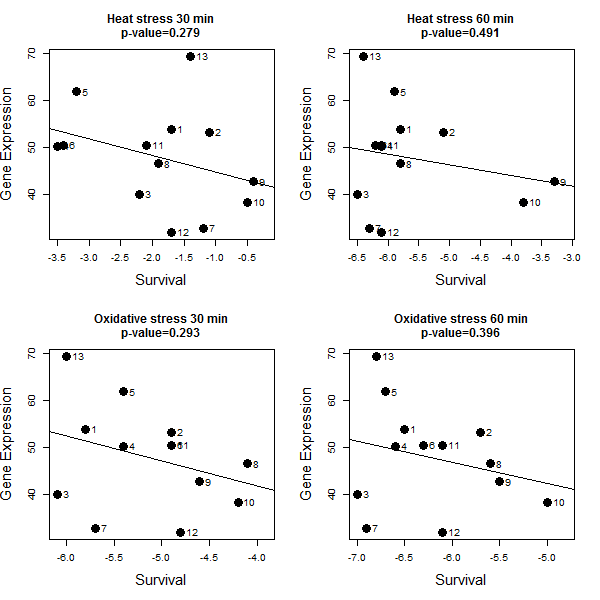

Supplement: Additional file 3: — Plots of gene expression and robustness levels. Expression levels of all genes plotted against survival after 30 and 60 minutes heat and oxidative stress (A: genes llmg_0001 to llmg_1229, B: genes llmg_1230 to llmg_2563). Survival is expressed as the difference of log CFU/ml after stress and before stress. Numbers indicate fermentations as presented in Table 1. P-values above the plots indicate significance of correlation (assessed by a linear model). [file 12934_2014_148_MOESM3_ESM.zip › Additional File 3A/llmg_0046_real_dat.png]

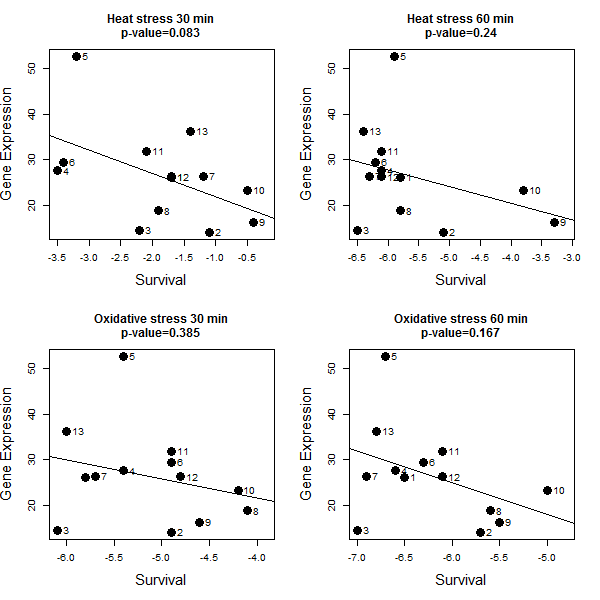

Supplement: Additional file 3: — Plots of gene expression and robustness levels. Expression levels of all genes plotted against survival after 30 and 60 minutes heat and oxidative stress (A: genes llmg_0001 to llmg_1229, B: genes llmg_1230 to llmg_2563). Survival is expressed as the difference of log CFU/ml after stress and before stress. Numbers indicate fermentations as presented in Table 1. P-values above the plots indicate significance of correlation (assessed by a linear model). [file 12934_2014_148_MOESM3_ESM.zip › Additional File 3A/llmg_0047_real_dat.png]

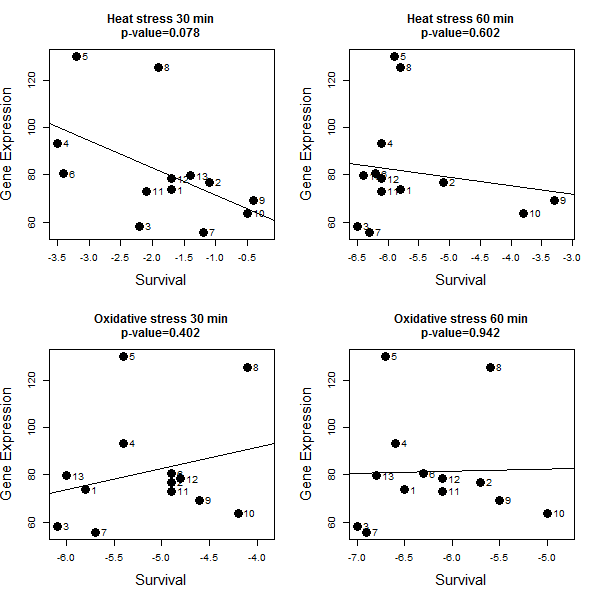

Supplement: Additional file 3: — Plots of gene expression and robustness levels. Expression levels of all genes plotted against survival after 30 and 60 minutes heat and oxidative stress (A: genes llmg_0001 to llmg_1229, B: genes llmg_1230 to llmg_2563). Survival is expressed as the difference of log CFU/ml after stress and before stress. Numbers indicate fermentations as presented in Table 1. P-values above the plots indicate significance of correlation (assessed by a linear model). [file 12934_2014_148_MOESM3_ESM.zip › Additional File 3A/llmg_0048_real_dat.png]

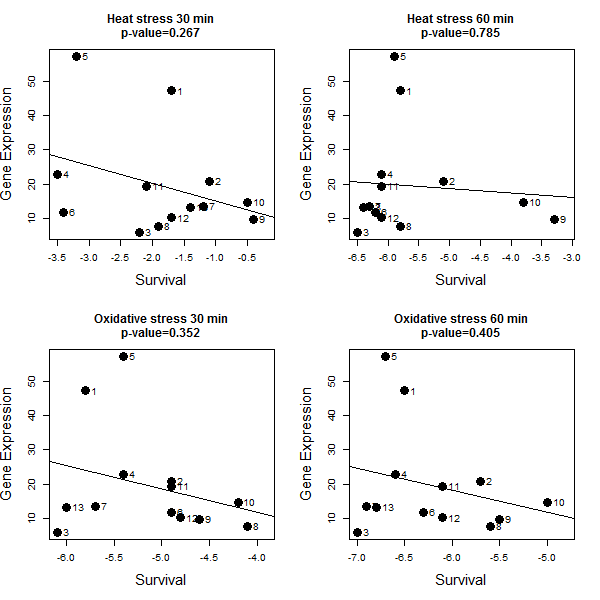

Supplement: Additional file 3: — Plots of gene expression and robustness levels. Expression levels of all genes plotted against survival after 30 and 60 minutes heat and oxidative stress (A: genes llmg_0001 to llmg_1229, B: genes llmg_1230 to llmg_2563). Survival is expressed as the difference of log CFU/ml after stress and before stress. Numbers indicate fermentations as presented in Table 1. P-values above the plots indicate significance of correlation (assessed by a linear model). [file 12934_2014_148_MOESM3_ESM.zip › Additional File 3A/llmg_0051_real_dat.png]

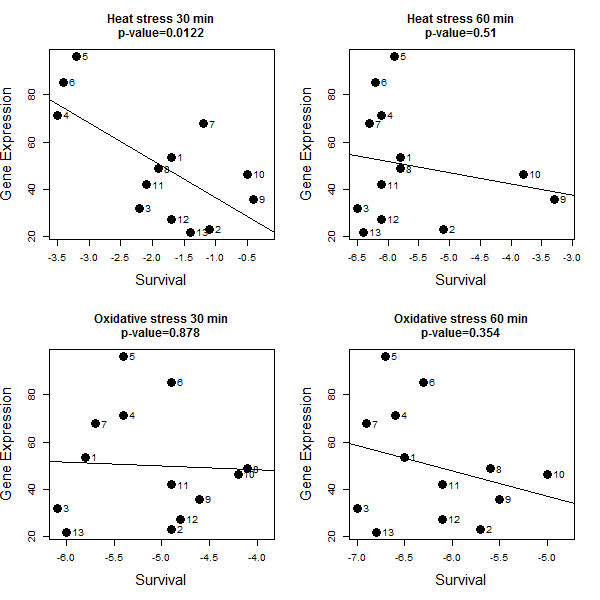

Supplement: Additional file 3: — Plots of gene expression and robustness levels. Expression levels of all genes plotted against survival after 30 and 60 minutes heat and oxidative stress (A: genes llmg_0001 to llmg_1229, B: genes llmg_1230 to llmg_2563). Survival is expressed as the difference of log CFU/ml after stress and before stress. Numbers indicate fermentations as presented in Table 1. P-values above the plots indicate significance of correlation (assessed by a linear model). [file 12934_2014_148_MOESM3_ESM.zip › Additional File 3A/llmg_0052_real_dat.png]

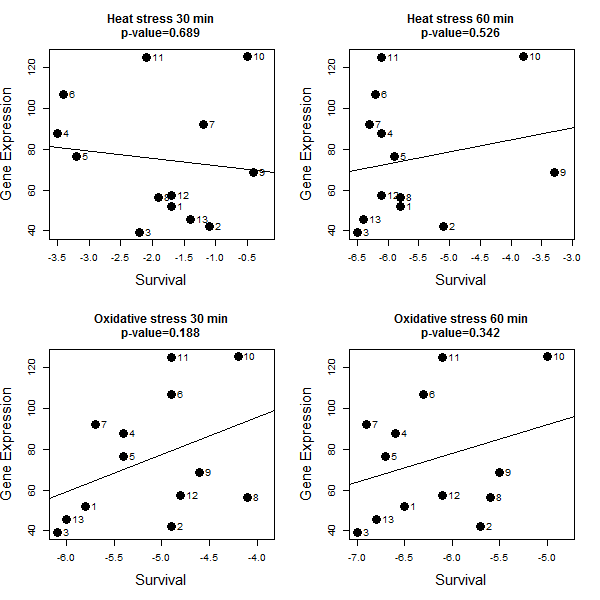

Supplement: Additional file 3: — Plots of gene expression and robustness levels. Expression levels of all genes plotted against survival after 30 and 60 minutes heat and oxidative stress (A: genes llmg_0001 to llmg_1229, B: genes llmg_1230 to llmg_2563). Survival is expressed as the difference of log CFU/ml after stress and before stress. Numbers indicate fermentations as presented in Table 1. P-values above the plots indicate significance of correlation (assessed by a linear model). [file 12934_2014_148_MOESM3_ESM.zip › Additional File 3A/llmg_0053_real_dat.png]

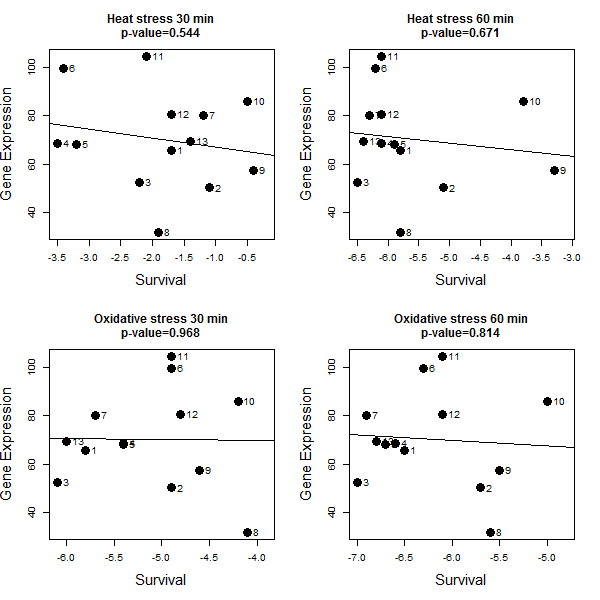

Supplement: Additional file 3: — Plots of gene expression and robustness levels. Expression levels of all genes plotted against survival after 30 and 60 minutes heat and oxidative stress (A: genes llmg_0001 to llmg_1229, B: genes llmg_1230 to llmg_2563). Survival is expressed as the difference of log CFU/ml after stress and before stress. Numbers indicate fermentations as presented in Table 1. P-values above the plots indicate significance of correlation (assessed by a linear model). [file 12934_2014_148_MOESM3_ESM.zip › Additional File 3A/llmg_0054_real_dat.png]

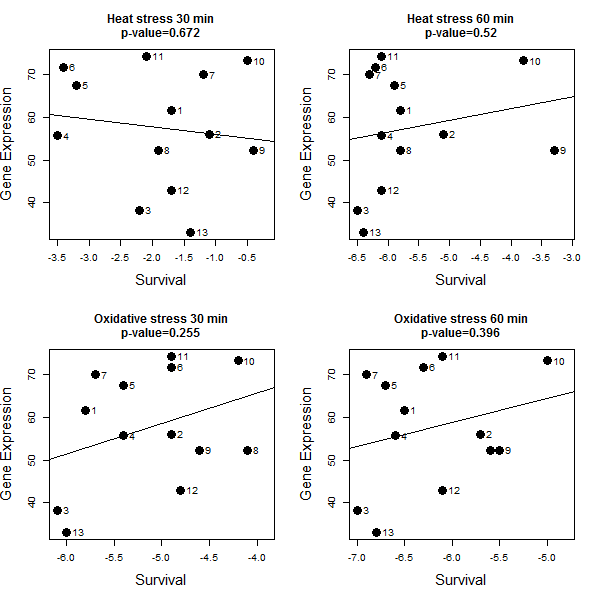

Supplement: Additional file 3: — Plots of gene expression and robustness levels. Expression levels of all genes plotted against survival after 30 and 60 minutes heat and oxidative stress (A: genes llmg_0001 to llmg_1229, B: genes llmg_1230 to llmg_2563). Survival is expressed as the difference of log CFU/ml after stress and before stress. Numbers indicate fermentations as presented in Table 1. P-values above the plots indicate significance of correlation (assessed by a linear model). [file 12934_2014_148_MOESM3_ESM.zip › Additional File 3A/llmg_0055_real_dat.png]

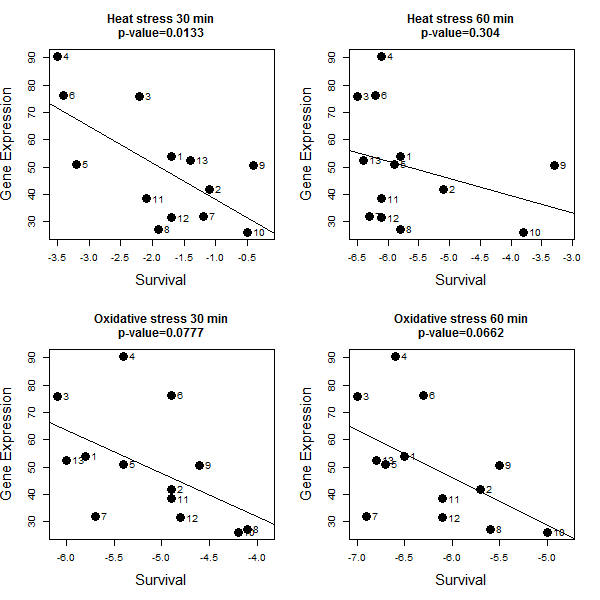

Supplement: Additional file 3: — Plots of gene expression and robustness levels. Expression levels of all genes plotted against survival after 30 and 60 minutes heat and oxidative stress (A: genes llmg_0001 to llmg_1229, B: genes llmg_1230 to llmg_2563). Survival is expressed as the difference of log CFU/ml after stress and before stress. Numbers indicate fermentations as presented in Table 1. P-values above the plots indicate significance of correlation (assessed by a linear model). [file 12934_2014_148_MOESM3_ESM.zip › Additional File 3A/llmg_0056_real_dat.png]

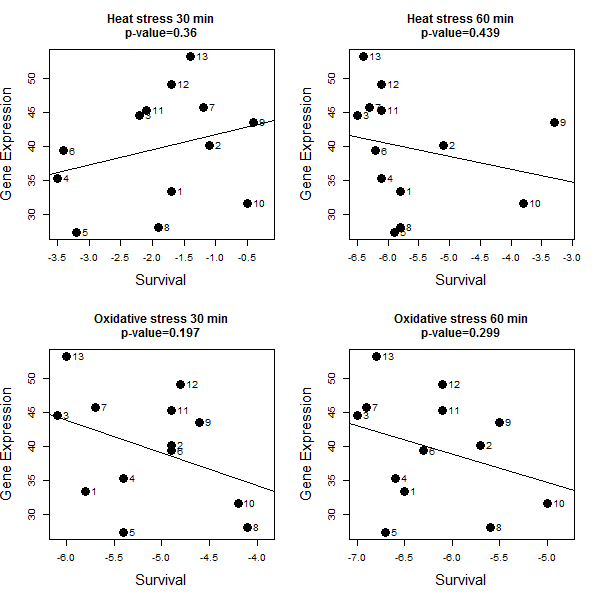

Supplement: Additional file 3: — Plots of gene expression and robustness levels. Expression levels of all genes plotted against survival after 30 and 60 minutes heat and oxidative stress (A: genes llmg_0001 to llmg_1229, B: genes llmg_1230 to llmg_2563). Survival is expressed as the difference of log CFU/ml after stress and before stress. Numbers indicate fermentations as presented in Table 1. P-values above the plots indicate significance of correlation (assessed by a linear model). [file 12934_2014_148_MOESM3_ESM.zip › Additional File 3A/llmg_0057_real_dat.png]

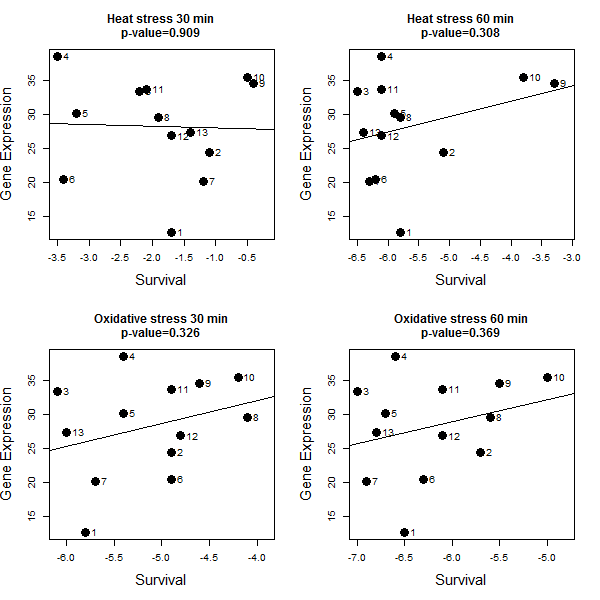

Supplement: Additional file 3: — Plots of gene expression and robustness levels. Expression levels of all genes plotted against survival after 30 and 60 minutes heat and oxidative stress (A: genes llmg_0001 to llmg_1229, B: genes llmg_1230 to llmg_2563). Survival is expressed as the difference of log CFU/ml after stress and before stress. Numbers indicate fermentations as presented in Table 1. P-values above the plots indicate significance of correlation (assessed by a linear model). [file 12934_2014_148_MOESM3_ESM.zip › Additional File 3A/llmg_0059_real_dat.png]

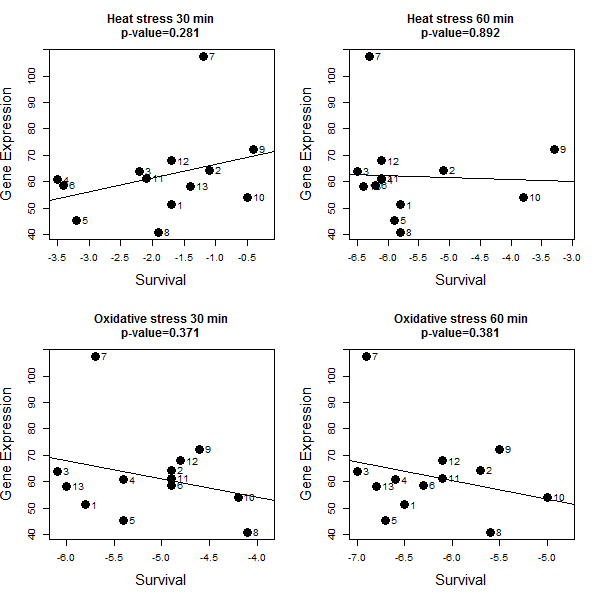

Supplement: Additional file 3: — Plots of gene expression and robustness levels. Expression levels of all genes plotted against survival after 30 and 60 minutes heat and oxidative stress (A: genes llmg_0001 to llmg_1229, B: genes llmg_1230 to llmg_2563). Survival is expressed as the difference of log CFU/ml after stress and before stress. Numbers indicate fermentations as presented in Table 1. P-values above the plots indicate significance of correlation (assessed by a linear model). [file 12934_2014_148_MOESM3_ESM.zip › Additional File 3A/llmg_0060_real_dat.png]

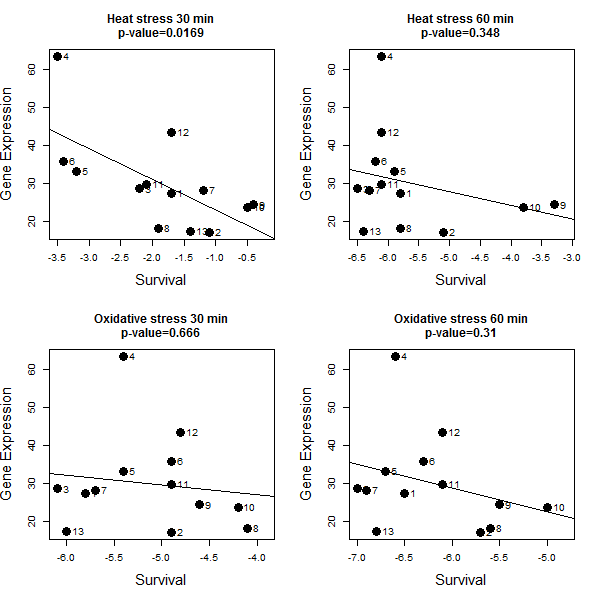

Supplement: Additional file 3: — Plots of gene expression and robustness levels. Expression levels of all genes plotted against survival after 30 and 60 minutes heat and oxidative stress (A: genes llmg_0001 to llmg_1229, B: genes llmg_1230 to llmg_2563). Survival is expressed as the difference of log CFU/ml after stress and before stress. Numbers indicate fermentations as presented in Table 1. P-values above the plots indicate significance of correlation (assessed by a linear model). [file 12934_2014_148_MOESM3_ESM.zip › Additional File 3A/llmg_0061_real_dat.png]

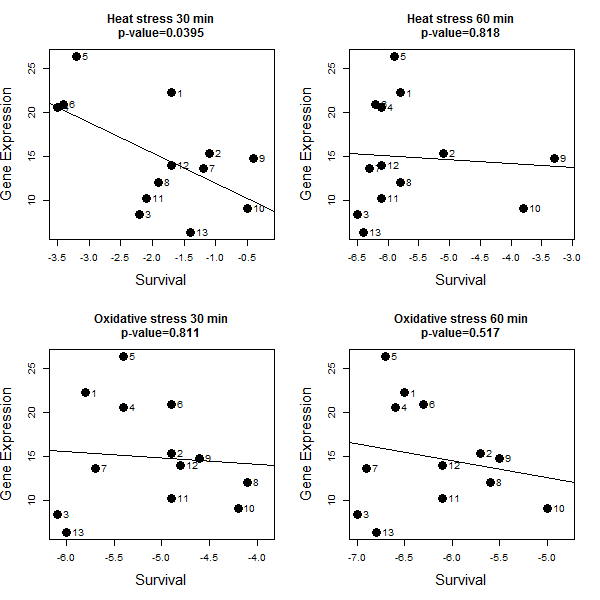

Supplement: Additional file 3: — Plots of gene expression and robustness levels. Expression levels of all genes plotted against survival after 30 and 60 minutes heat and oxidative stress (A: genes llmg_0001 to llmg_1229, B: genes llmg_1230 to llmg_2563). Survival is expressed as the difference of log CFU/ml after stress and before stress. Numbers indicate fermentations as presented in Table 1. P-values above the plots indicate significance of correlation (assessed by a linear model). [file 12934_2014_148_MOESM3_ESM.zip › Additional File 3A/llmg_0062_real_dat.png]

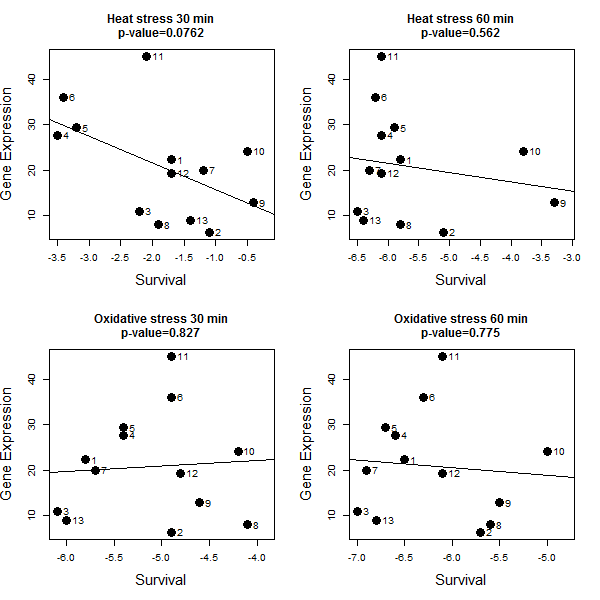

Supplement: Additional file 3: — Plots of gene expression and robustness levels. Expression levels of all genes plotted against survival after 30 and 60 minutes heat and oxidative stress (A: genes llmg_0001 to llmg_1229, B: genes llmg_1230 to llmg_2563). Survival is expressed as the difference of log CFU/ml after stress and before stress. Numbers indicate fermentations as presented in Table 1. P-values above the plots indicate significance of correlation (assessed by a linear model). [file 12934_2014_148_MOESM3_ESM.zip › Additional File 3A/llmg_0063_real_dat.png]

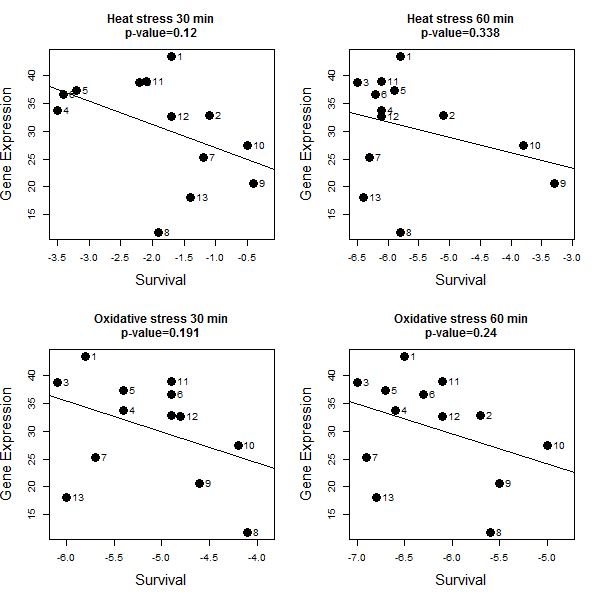

Supplement: Additional file 3: — Plots of gene expression and robustness levels. Expression levels of all genes plotted against survival after 30 and 60 minutes heat and oxidative stress (A: genes llmg_0001 to llmg_1229, B: genes llmg_1230 to llmg_2563). Survival is expressed as the difference of log CFU/ml after stress and before stress. Numbers indicate fermentations as presented in Table 1. P-values above the plots indicate significance of correlation (assessed by a linear model). [file 12934_2014_148_MOESM3_ESM.zip › Additional File 3A/llmg_0065_real_dat.png]

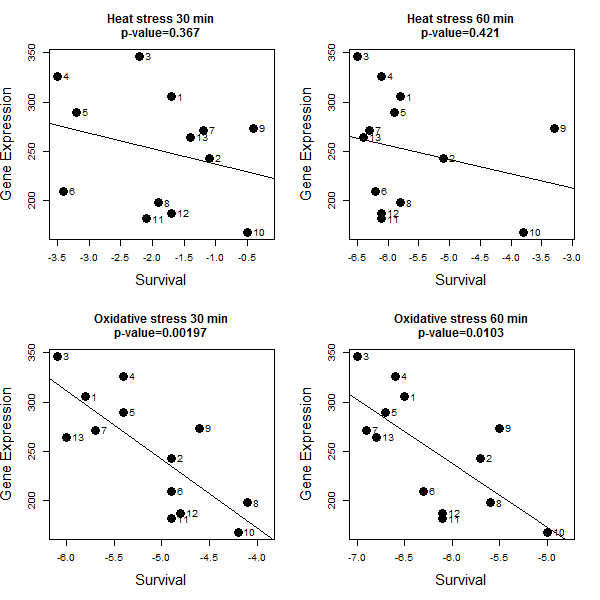

Supplement: Additional file 3: — Plots of gene expression and robustness levels. Expression levels of all genes plotted against survival after 30 and 60 minutes heat and oxidative stress (A: genes llmg_0001 to llmg_1229, B: genes llmg_1230 to llmg_2563). Survival is expressed as the difference of log CFU/ml after stress and before stress. Numbers indicate fermentations as presented in Table 1. P-values above the plots indicate significance of correlation (assessed by a linear model). [file 12934_2014_148_MOESM3_ESM.zip › Additional File 3A/llmg_0066_real_dat.png]

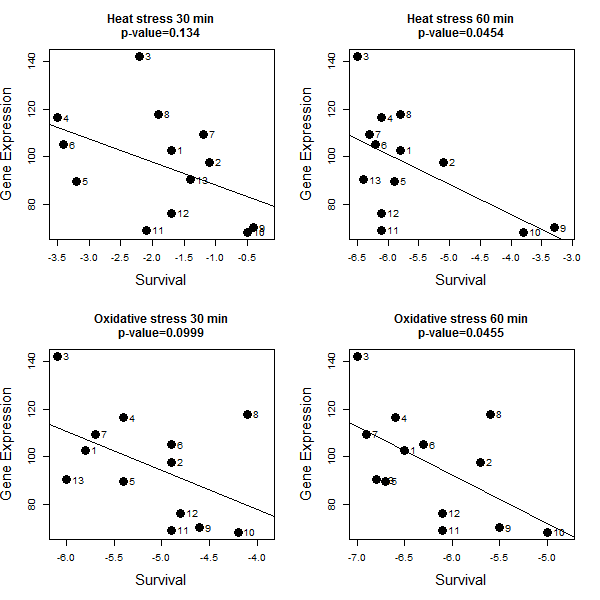

Supplement: Additional file 3: — Plots of gene expression and robustness levels. Expression levels of all genes plotted against survival after 30 and 60 minutes heat and oxidative stress (A: genes llmg_0001 to llmg_1229, B: genes llmg_1230 to llmg_2563). Survival is expressed as the difference of log CFU/ml after stress and before stress. Numbers indicate fermentations as presented in Table 1. P-values above the plots indicate significance of correlation (assessed by a linear model). [file 12934_2014_148_MOESM3_ESM.zip › Additional File 3A/llmg_0067_real_dat.png]

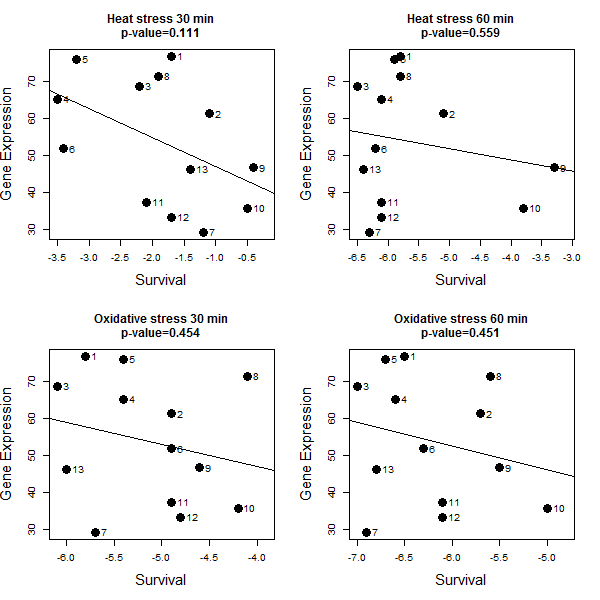

Supplement: Additional file 3: — Plots of gene expression and robustness levels. Expression levels of all genes plotted against survival after 30 and 60 minutes heat and oxidative stress (A: genes llmg_0001 to llmg_1229, B: genes llmg_1230 to llmg_2563). Survival is expressed as the difference of log CFU/ml after stress and before stress. Numbers indicate fermentations as presented in Table 1. P-values above the plots indicate significance of correlation (assessed by a linear model). [file 12934_2014_148_MOESM3_ESM.zip › Additional File 3A/llmg_0069_real_dat.png]

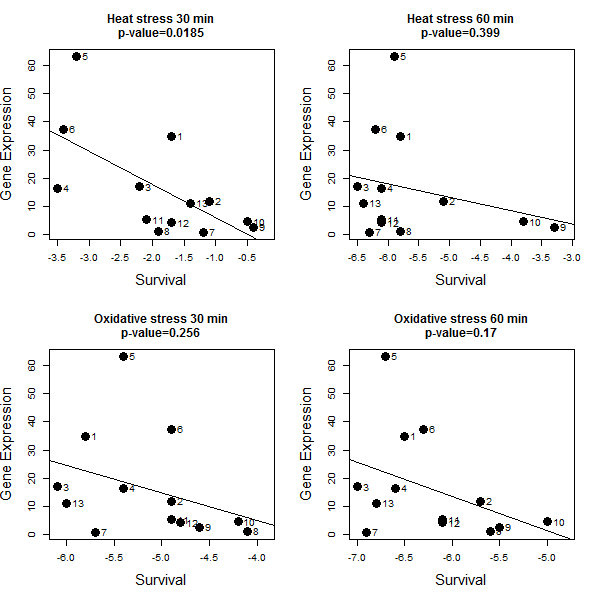

Supplement: Additional file 3: — Plots of gene expression and robustness levels. Expression levels of all genes plotted against survival after 30 and 60 minutes heat and oxidative stress (A: genes llmg_0001 to llmg_1229, B: genes llmg_1230 to llmg_2563). Survival is expressed as the difference of log CFU/ml after stress and before stress. Numbers indicate fermentations as presented in Table 1. P-values above the plots indicate significance of correlation (assessed by a linear model). [file 12934_2014_148_MOESM3_ESM.zip › Additional File 3A/llmg_0070_real_dat.png]

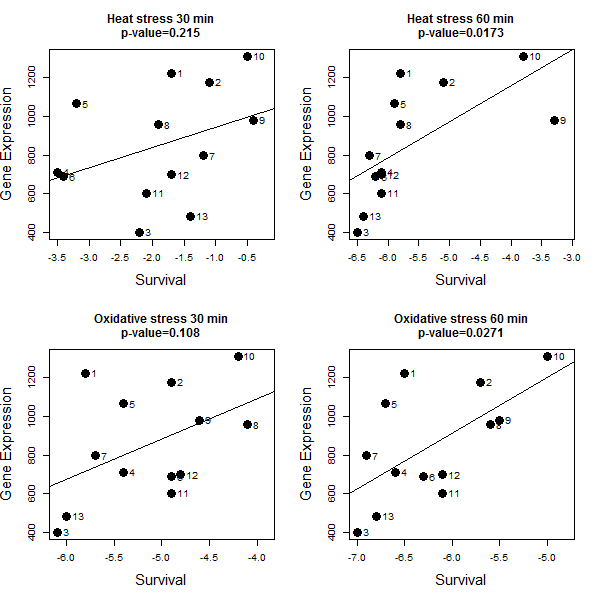

Supplement: Additional file 3: — Plots of gene expression and robustness levels. Expression levels of all genes plotted against survival after 30 and 60 minutes heat and oxidative stress (A: genes llmg_0001 to llmg_1229, B: genes llmg_1230 to llmg_2563). Survival is expressed as the difference of log CFU/ml after stress and before stress. Numbers indicate fermentations as presented in Table 1. P-values above the plots indicate significance of correlation (assessed by a linear model). [file 12934_2014_148_MOESM3_ESM.zip › Additional File 3A/llmg_0071_real_dat.png]

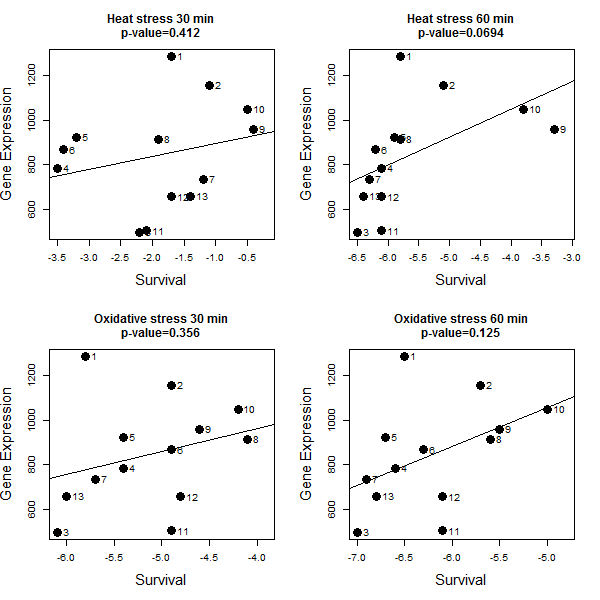

Supplement: Additional file 3: — Plots of gene expression and robustness levels. Expression levels of all genes plotted against survival after 30 and 60 minutes heat and oxidative stress (A: genes llmg_0001 to llmg_1229, B: genes llmg_1230 to llmg_2563). Survival is expressed as the difference of log CFU/ml after stress and before stress. Numbers indicate fermentations as presented in Table 1. P-values above the plots indicate significance of correlation (assessed by a linear model). [file 12934_2014_148_MOESM3_ESM.zip › Additional File 3A/llmg_0072_real_dat.png]

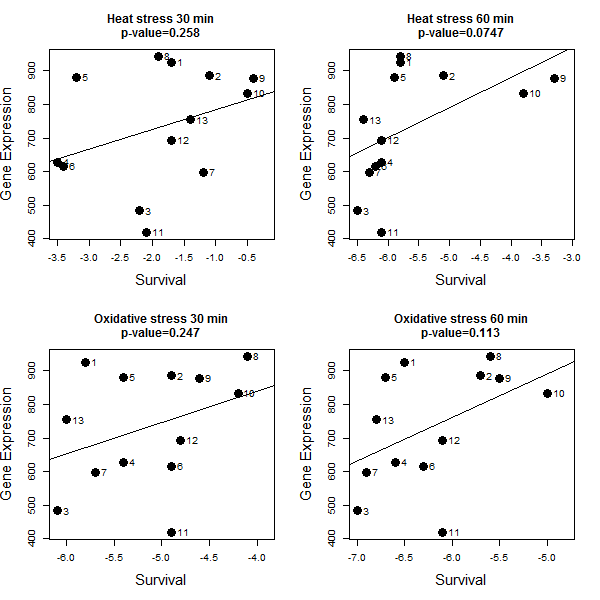

Supplement: Additional file 3: — Plots of gene expression and robustness levels. Expression levels of all genes plotted against survival after 30 and 60 minutes heat and oxidative stress (A: genes llmg_0001 to llmg_1229, B: genes llmg_1230 to llmg_2563). Survival is expressed as the difference of log CFU/ml after stress and before stress. Numbers indicate fermentations as presented in Table 1. P-values above the plots indicate significance of correlation (assessed by a linear model). [file 12934_2014_148_MOESM3_ESM.zip › Additional File 3A/llmg_0073_real_dat.png]

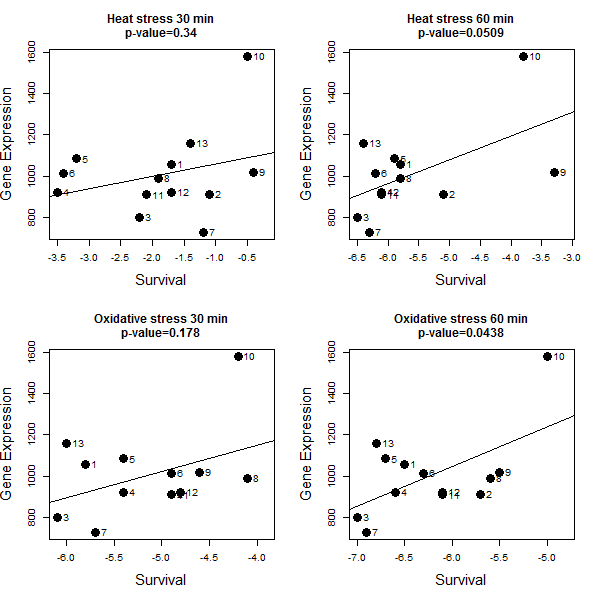

Supplement: Additional file 3: — Plots of gene expression and robustness levels. Expression levels of all genes plotted against survival after 30 and 60 minutes heat and oxidative stress (A: genes llmg_0001 to llmg_1229, B: genes llmg_1230 to llmg_2563). Survival is expressed as the difference of log CFU/ml after stress and before stress. Numbers indicate fermentations as presented in Table 1. P-values above the plots indicate significance of correlation (assessed by a linear model). [file 12934_2014_148_MOESM3_ESM.zip › Additional File 3A/llmg_0074_real_dat.png]

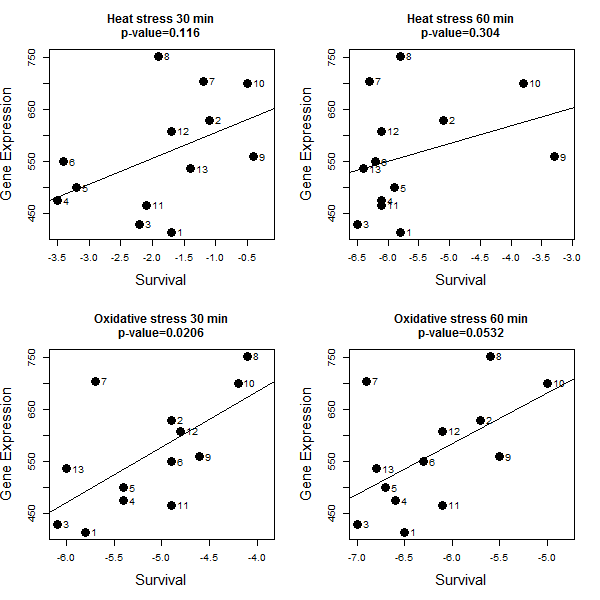

Supplement: Additional file 3: — Plots of gene expression and robustness levels. Expression levels of all genes plotted against survival after 30 and 60 minutes heat and oxidative stress (A: genes llmg_0001 to llmg_1229, B: genes llmg_1230 to llmg_2563). Survival is expressed as the difference of log CFU/ml after stress and before stress. Numbers indicate fermentations as presented in Table 1. P-values above the plots indicate significance of correlation (assessed by a linear model). [file 12934_2014_148_MOESM3_ESM.zip › Additional File 3A/llmg_0075_real_dat.png]

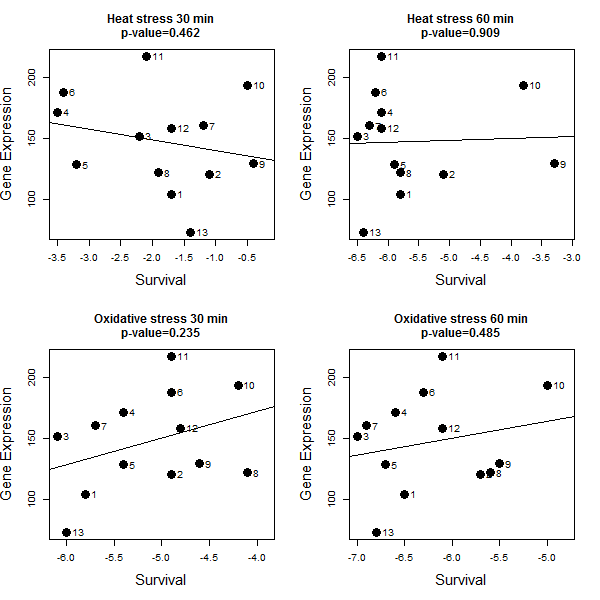

Supplement: Additional file 3: — Plots of gene expression and robustness levels. Expression levels of all genes plotted against survival after 30 and 60 minutes heat and oxidative stress (A: genes llmg_0001 to llmg_1229, B: genes llmg_1230 to llmg_2563). Survival is expressed as the difference of log CFU/ml after stress and before stress. Numbers indicate fermentations as presented in Table 1. P-values above the plots indicate significance of correlation (assessed by a linear model). [file 12934_2014_148_MOESM3_ESM.zip › Additional File 3A/llmg_0076_real_dat.png]

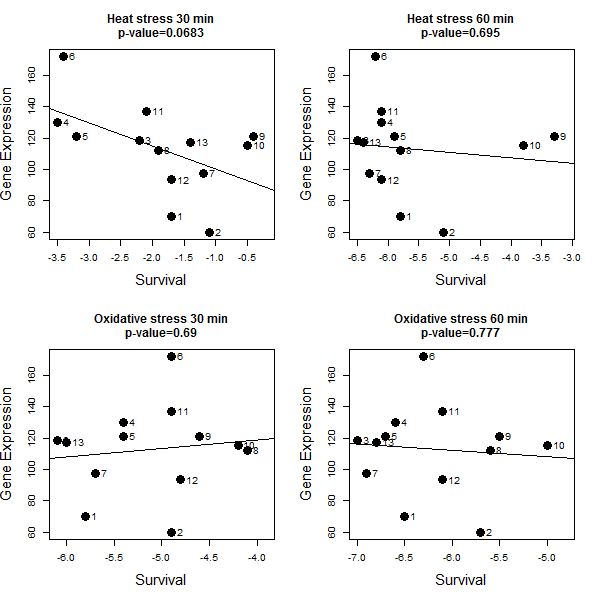

Supplement: Additional file 3: — Plots of gene expression and robustness levels. Expression levels of all genes plotted against survival after 30 and 60 minutes heat and oxidative stress (A: genes llmg_0001 to llmg_1229, B: genes llmg_1230 to llmg_2563). Survival is expressed as the difference of log CFU/ml after stress and before stress. Numbers indicate fermentations as presented in Table 1. P-values above the plots indicate significance of correlation (assessed by a linear model). [file 12934_2014_148_MOESM3_ESM.zip › Additional File 3A/llmg_0077_real_dat.png]

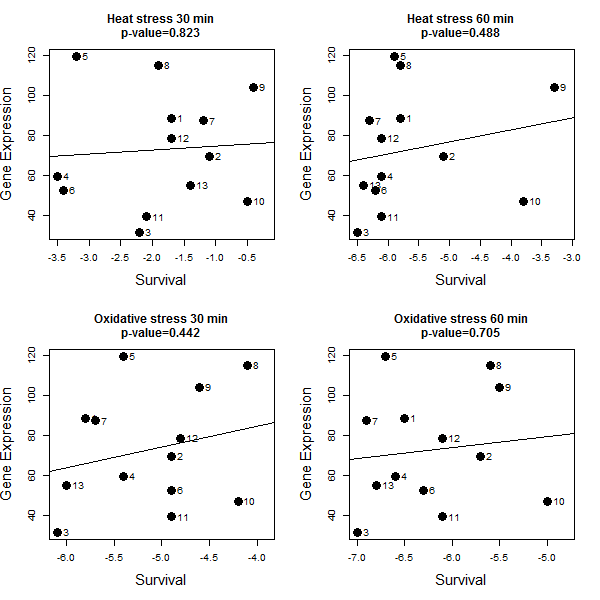

Supplement: Additional file 3: — Plots of gene expression and robustness levels. Expression levels of all genes plotted against survival after 30 and 60 minutes heat and oxidative stress (A: genes llmg_0001 to llmg_1229, B: genes llmg_1230 to llmg_2563). Survival is expressed as the difference of log CFU/ml after stress and before stress. Numbers indicate fermentations as presented in Table 1. P-values above the plots indicate significance of correlation (assessed by a linear model). [file 12934_2014_148_MOESM3_ESM.zip › Additional File 3A/llmg_0078_real_dat.png]

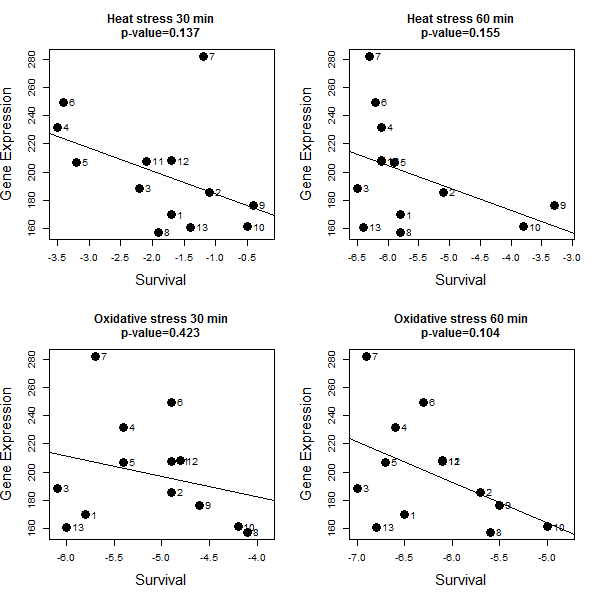

Supplement: Additional file 3: — Plots of gene expression and robustness levels. Expression levels of all genes plotted against survival after 30 and 60 minutes heat and oxidative stress (A: genes llmg_0001 to llmg_1229, B: genes llmg_1230 to llmg_2563). Survival is expressed as the difference of log CFU/ml after stress and before stress. Numbers indicate fermentations as presented in Table 1. P-values above the plots indicate significance of correlation (assessed by a linear model). [file 12934_2014_148_MOESM3_ESM.zip › Additional File 3A/llmg_0079_real_dat.png]

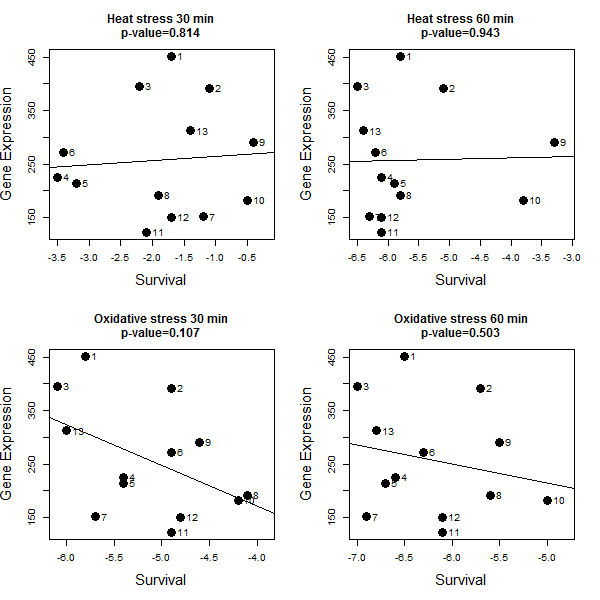

Supplement: Additional file 3: — Plots of gene expression and robustness levels. Expression levels of all genes plotted against survival after 30 and 60 minutes heat and oxidative stress (A: genes llmg_0001 to llmg_1229, B: genes llmg_1230 to llmg_2563). Survival is expressed as the difference of log CFU/ml after stress and before stress. Numbers indicate fermentations as presented in Table 1. P-values above the plots indicate significance of correlation (assessed by a linear model). [file 12934_2014_148_MOESM3_ESM.zip › Additional File 3A/llmg_0080_real_dat.png]

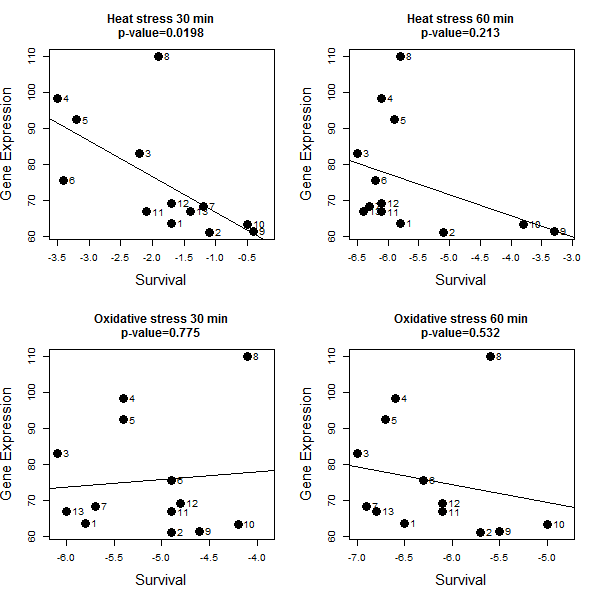

Supplement: Additional file 3: — Plots of gene expression and robustness levels. Expression levels of all genes plotted against survival after 30 and 60 minutes heat and oxidative stress (A: genes llmg_0001 to llmg_1229, B: genes llmg_1230 to llmg_2563). Survival is expressed as the difference of log CFU/ml after stress and before stress. Numbers indicate fermentations as presented in Table 1. P-values above the plots indicate significance of correlation (assessed by a linear model). [file 12934_2014_148_MOESM3_ESM.zip › Additional File 3A/llmg_0081_real_dat.png]

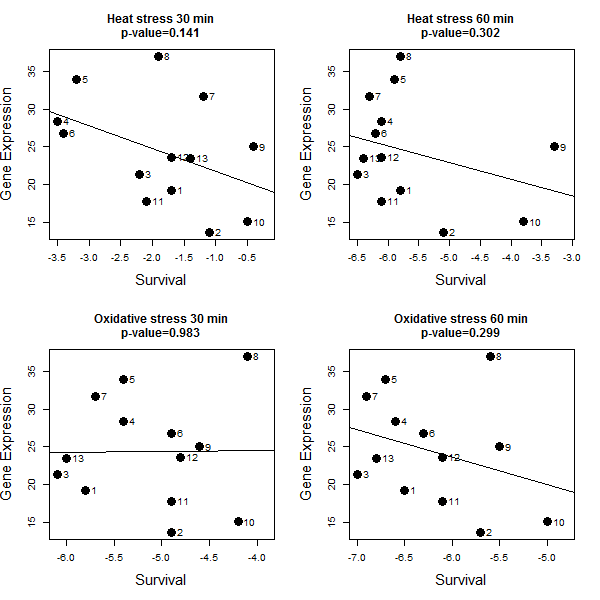

Supplement: Additional file 3: — Plots of gene expression and robustness levels. Expression levels of all genes plotted against survival after 30 and 60 minutes heat and oxidative stress (A: genes llmg_0001 to llmg_1229, B: genes llmg_1230 to llmg_2563). Survival is expressed as the difference of log CFU/ml after stress and before stress. Numbers indicate fermentations as presented in Table 1. P-values above the plots indicate significance of correlation (assessed by a linear model). [file 12934_2014_148_MOESM3_ESM.zip › Additional File 3A/llmg_0082_real_dat.png]

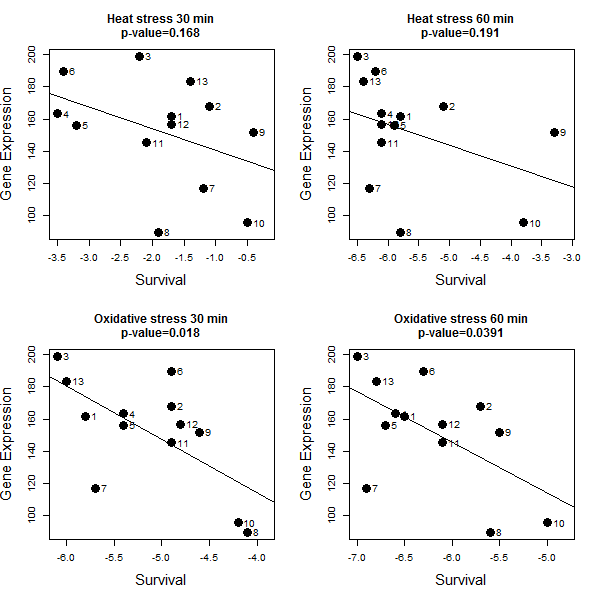

Supplement: Additional file 3: — Plots of gene expression and robustness levels. Expression levels of all genes plotted against survival after 30 and 60 minutes heat and oxidative stress (A: genes llmg_0001 to llmg_1229, B: genes llmg_1230 to llmg_2563). Survival is expressed as the difference of log CFU/ml after stress and before stress. Numbers indicate fermentations as presented in Table 1. P-values above the plots indicate significance of correlation (assessed by a linear model). [file 12934_2014_148_MOESM3_ESM.zip › Additional File 3A/llmg_0083_real_dat.png]

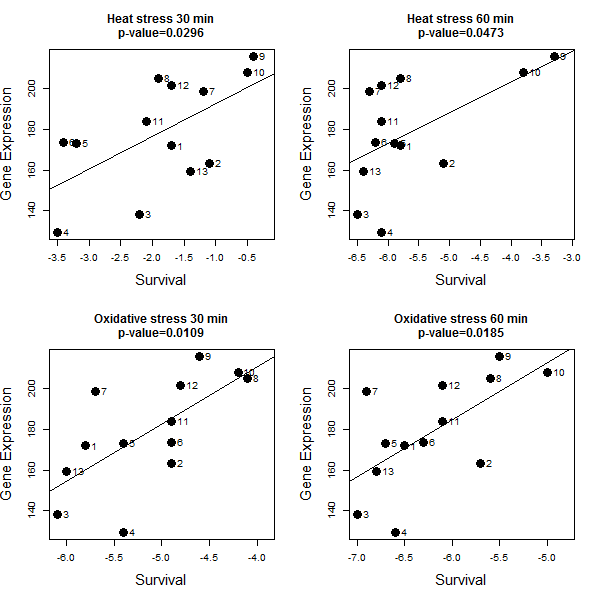

Supplement: Additional file 3: — Plots of gene expression and robustness levels. Expression levels of all genes plotted against survival after 30 and 60 minutes heat and oxidative stress (A: genes llmg_0001 to llmg_1229, B: genes llmg_1230 to llmg_2563). Survival is expressed as the difference of log CFU/ml after stress and before stress. Numbers indicate fermentations as presented in Table 1. P-values above the plots indicate significance of correlation (assessed by a linear model). [file 12934_2014_148_MOESM3_ESM.zip › Additional File 3A/llmg_0084_real_dat.png]

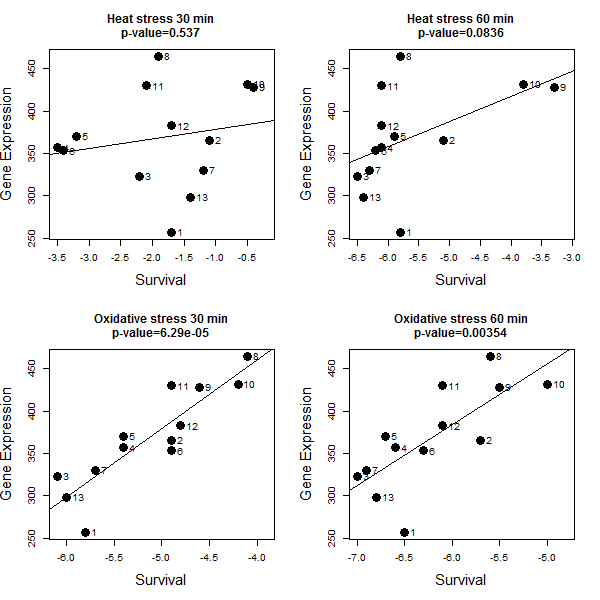

Supplement: Additional file 3: — Plots of gene expression and robustness levels. Expression levels of all genes plotted against survival after 30 and 60 minutes heat and oxidative stress (A: genes llmg_0001 to llmg_1229, B: genes llmg_1230 to llmg_2563). Survival is expressed as the difference of log CFU/ml after stress and before stress. Numbers indicate fermentations as presented in Table 1. P-values above the plots indicate significance of correlation (assessed by a linear model). [file 12934_2014_148_MOESM3_ESM.zip › Additional File 3A/llmg_0085_real_dat.png]

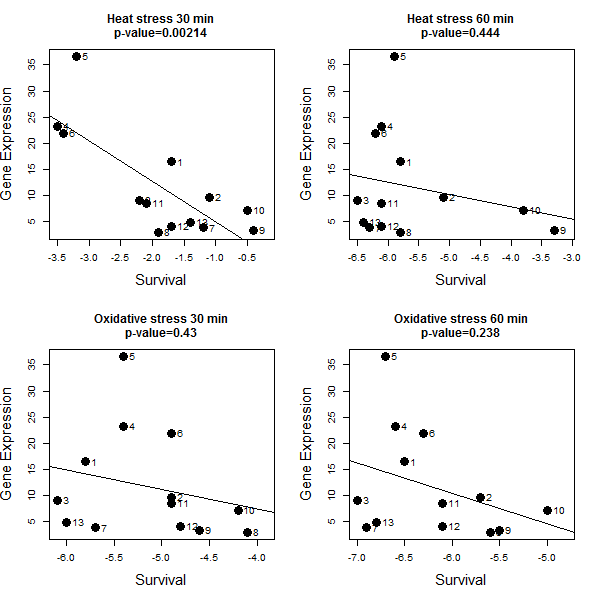

Supplement: Additional file 3: — Plots of gene expression and robustness levels. Expression levels of all genes plotted against survival after 30 and 60 minutes heat and oxidative stress (A: genes llmg_0001 to llmg_1229, B: genes llmg_1230 to llmg_2563). Survival is expressed as the difference of log CFU/ml after stress and before stress. Numbers indicate fermentations as presented in Table 1. P-values above the plots indicate significance of correlation (assessed by a linear model). [file 12934_2014_148_MOESM3_ESM.zip › Additional File 3A/llmg_0086_real_dat.png]

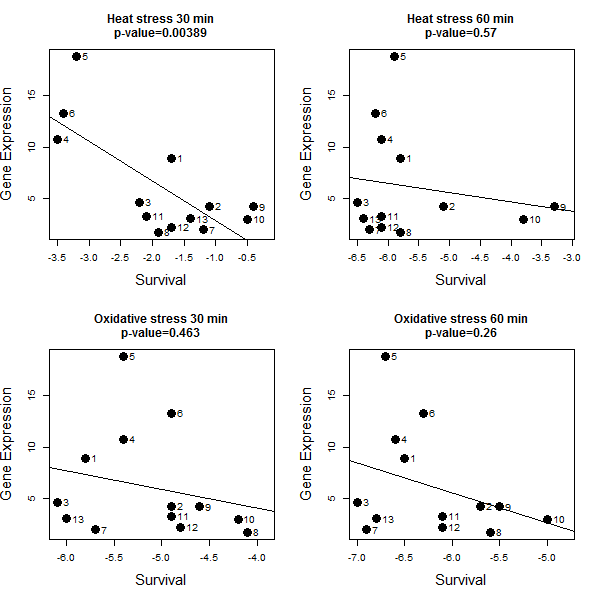

Supplement: Additional file 3: — Plots of gene expression and robustness levels. Expression levels of all genes plotted against survival after 30 and 60 minutes heat and oxidative stress (A: genes llmg_0001 to llmg_1229, B: genes llmg_1230 to llmg_2563). Survival is expressed as the difference of log CFU/ml after stress and before stress. Numbers indicate fermentations as presented in Table 1. P-values above the plots indicate significance of correlation (assessed by a linear model). [file 12934_2014_148_MOESM3_ESM.zip › Additional File 3A/llmg_0087_real_dat.png]

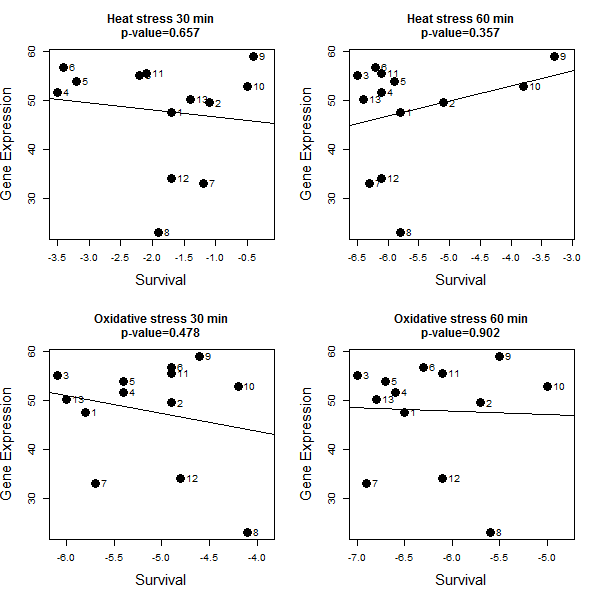

Supplement: Additional file 3: — Plots of gene expression and robustness levels. Expression levels of all genes plotted against survival after 30 and 60 minutes heat and oxidative stress (A: genes llmg_0001 to llmg_1229, B: genes llmg_1230 to llmg_2563). Survival is expressed as the difference of log CFU/ml after stress and before stress. Numbers indicate fermentations as presented in Table 1. P-values above the plots indicate significance of correlation (assessed by a linear model). [file 12934_2014_148_MOESM3_ESM.zip › Additional File 3A/llmg_0089_real_dat.png]

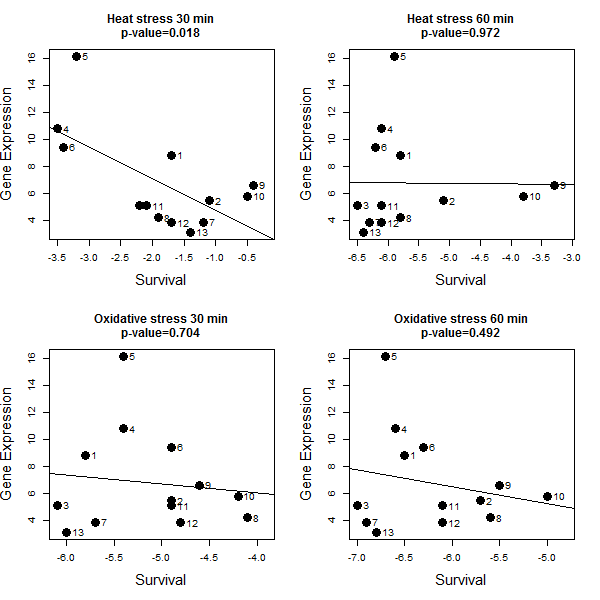

Supplement: Additional file 3: — Plots of gene expression and robustness levels. Expression levels of all genes plotted against survival after 30 and 60 minutes heat and oxidative stress (A: genes llmg_0001 to llmg_1229, B: genes llmg_1230 to llmg_2563). Survival is expressed as the difference of log CFU/ml after stress and before stress. Numbers indicate fermentations as presented in Table 1. P-values above the plots indicate significance of correlation (assessed by a linear model). [file 12934_2014_148_MOESM3_ESM.zip › Additional File 3A/llmg_0090_real_dat.png]

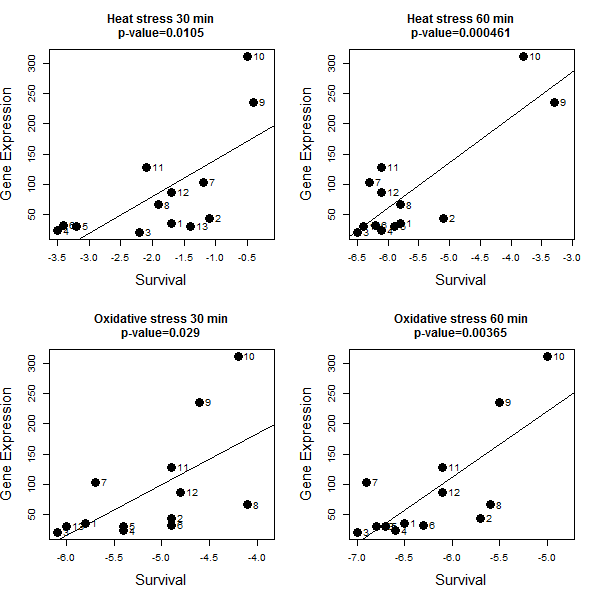

Supplement: Additional file 3: — Plots of gene expression and robustness levels. Expression levels of all genes plotted against survival after 30 and 60 minutes heat and oxidative stress (A: genes llmg_0001 to llmg_1229, B: genes llmg_1230 to llmg_2563). Survival is expressed as the difference of log CFU/ml after stress and before stress. Numbers indicate fermentations as presented in Table 1. P-values above the plots indicate significance of correlation (assessed by a linear model). [file 12934_2014_148_MOESM3_ESM.zip › Additional File 3A/llmg_0091_real_dat.png]

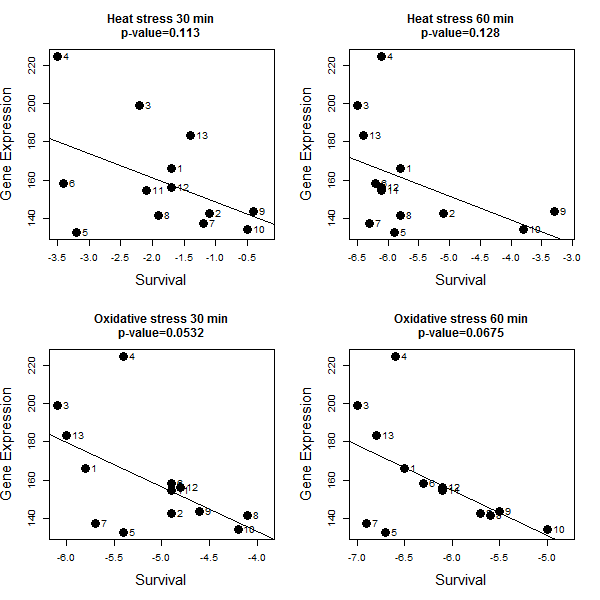

Supplement: Additional file 3: — Plots of gene expression and robustness levels. Expression levels of all genes plotted against survival after 30 and 60 minutes heat and oxidative stress (A: genes llmg_0001 to llmg_1229, B: genes llmg_1230 to llmg_2563). Survival is expressed as the difference of log CFU/ml after stress and before stress. Numbers indicate fermentations as presented in Table 1. P-values above the plots indicate significance of correlation (assessed by a linear model). [file 12934_2014_148_MOESM3_ESM.zip › Additional File 3A/llmg_0092_real_dat.png]

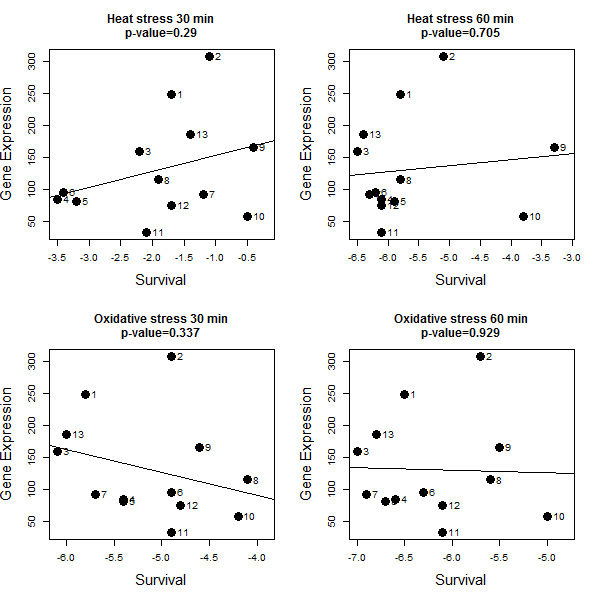

Supplement: Additional file 3: — Plots of gene expression and robustness levels. Expression levels of all genes plotted against survival after 30 and 60 minutes heat and oxidative stress (A: genes llmg_0001 to llmg_1229, B: genes llmg_1230 to llmg_2563). Survival is expressed as the difference of log CFU/ml after stress and before stress. Numbers indicate fermentations as presented in Table 1. P-values above the plots indicate significance of correlation (assessed by a linear model). [file 12934_2014_148_MOESM3_ESM.zip › Additional File 3A/llmg_0093_real_dat.png]

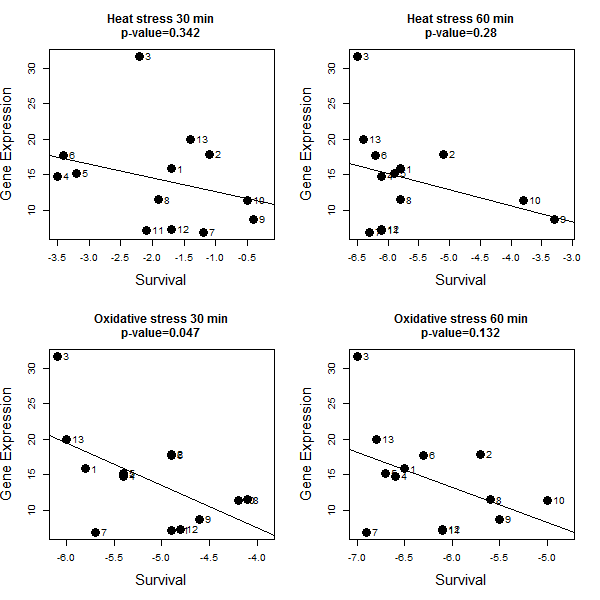

Supplement: Additional file 3: — Plots of gene expression and robustness levels. Expression levels of all genes plotted against survival after 30 and 60 minutes heat and oxidative stress (A: genes llmg_0001 to llmg_1229, B: genes llmg_1230 to llmg_2563). Survival is expressed as the difference of log CFU/ml after stress and before stress. Numbers indicate fermentations as presented in Table 1. P-values above the plots indicate significance of correlation (assessed by a linear model). [file 12934_2014_148_MOESM3_ESM.zip › Additional File 3A/llmg_0094_real_dat.png]

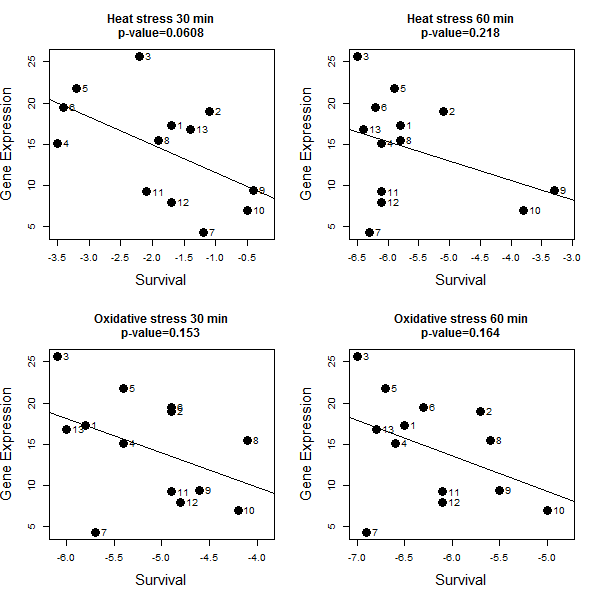

Supplement: Additional file 3: — Plots of gene expression and robustness levels. Expression levels of all genes plotted against survival after 30 and 60 minutes heat and oxidative stress (A: genes llmg_0001 to llmg_1229, B: genes llmg_1230 to llmg_2563). Survival is expressed as the difference of log CFU/ml after stress and before stress. Numbers indicate fermentations as presented in Table 1. P-values above the plots indicate significance of correlation (assessed by a linear model). [file 12934_2014_148_MOESM3_ESM.zip › Additional File 3A/llmg_0095_real_dat.png]

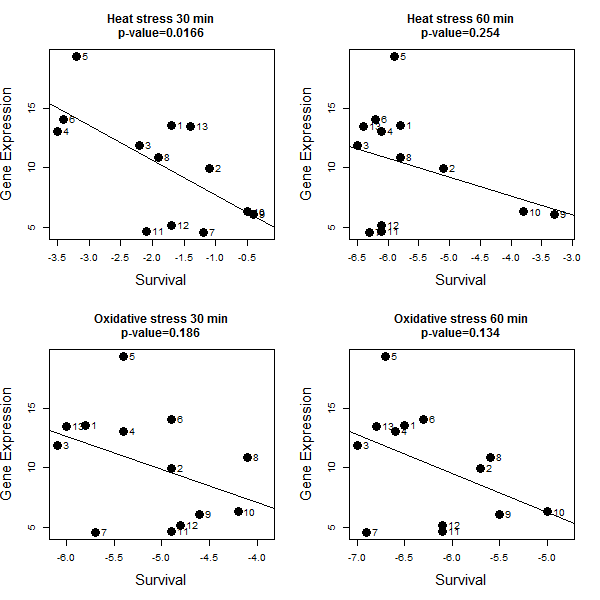

Supplement: Additional file 3: — Plots of gene expression and robustness levels. Expression levels of all genes plotted against survival after 30 and 60 minutes heat and oxidative stress (A: genes llmg_0001 to llmg_1229, B: genes llmg_1230 to llmg_2563). Survival is expressed as the difference of log CFU/ml after stress and before stress. Numbers indicate fermentations as presented in Table 1. P-values above the plots indicate significance of correlation (assessed by a linear model). [file 12934_2014_148_MOESM3_ESM.zip › Additional File 3A/llmg_0096_real_dat.png]

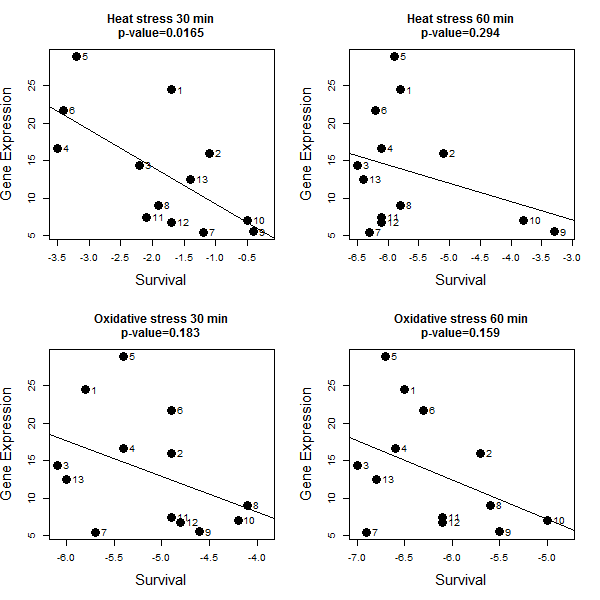

Supplement: Additional file 3: — Plots of gene expression and robustness levels. Expression levels of all genes plotted against survival after 30 and 60 minutes heat and oxidative stress (A: genes llmg_0001 to llmg_1229, B: genes llmg_1230 to llmg_2563). Survival is expressed as the difference of log CFU/ml after stress and before stress. Numbers indicate fermentations as presented in Table 1. P-values above the plots indicate significance of correlation (assessed by a linear model). [file 12934_2014_148_MOESM3_ESM.zip › Additional File 3A/llmg_0097_real_dat.png]

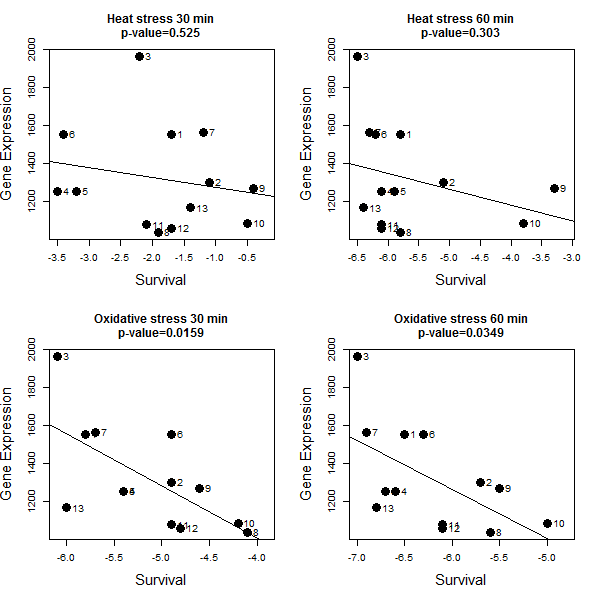

Supplement: Additional file 3: — Plots of gene expression and robustness levels. Expression levels of all genes plotted against survival after 30 and 60 minutes heat and oxidative stress (A: genes llmg_0001 to llmg_1229, B: genes llmg_1230 to llmg_2563). Survival is expressed as the difference of log CFU/ml after stress and before stress. Numbers indicate fermentations as presented in Table 1. P-values above the plots indicate significance of correlation (assessed by a linear model). [file 12934_2014_148_MOESM3_ESM.zip › Additional File 3A/llmg_0098_real_dat.png]

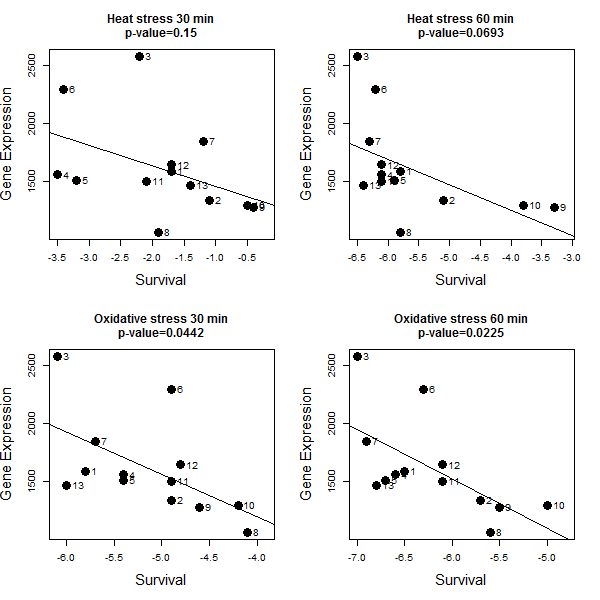

Supplement: Additional file 3: — Plots of gene expression and robustness levels. Expression levels of all genes plotted against survival after 30 and 60 minutes heat and oxidative stress (A: genes llmg_0001 to llmg_1229, B: genes llmg_1230 to llmg_2563). Survival is expressed as the difference of log CFU/ml after stress and before stress. Numbers indicate fermentations as presented in Table 1. P-values above the plots indicate significance of correlation (assessed by a linear model). [file 12934_2014_148_MOESM3_ESM.zip › Additional File 3A/llmg_0099_real_dat.png]

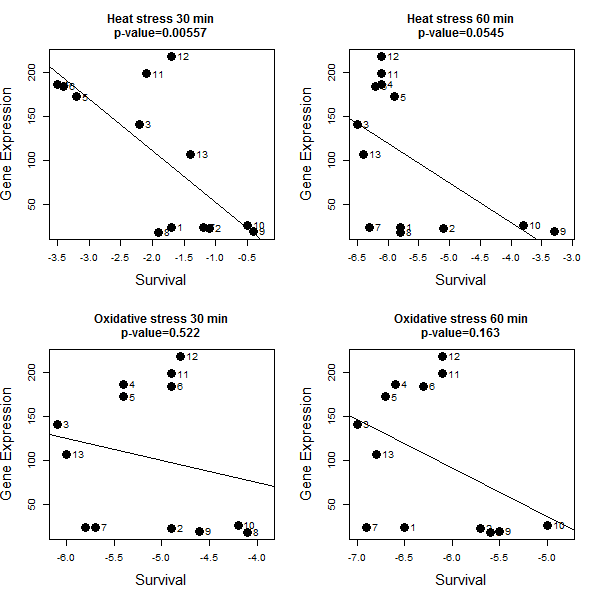

Supplement: Additional file 3: — Plots of gene expression and robustness levels. Expression levels of all genes plotted against survival after 30 and 60 minutes heat and oxidative stress (A: genes llmg_0001 to llmg_1229, B: genes llmg_1230 to llmg_2563). Survival is expressed as the difference of log CFU/ml after stress and before stress. Numbers indicate fermentations as presented in Table 1. P-values above the plots indicate significance of correlation (assessed by a linear model). [file 12934_2014_148_MOESM3_ESM.zip › Additional File 3A/llmg_0100_real_dat.png]

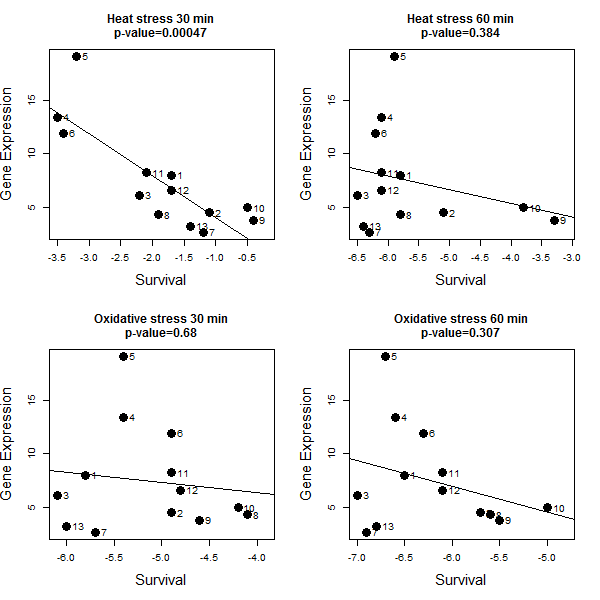

Supplement: Additional file 3: — Plots of gene expression and robustness levels. Expression levels of all genes plotted against survival after 30 and 60 minutes heat and oxidative stress (A: genes llmg_0001 to llmg_1229, B: genes llmg_1230 to llmg_2563). Survival is expressed as the difference of log CFU/ml after stress and before stress. Numbers indicate fermentations as presented in Table 1. P-values above the plots indicate significance of correlation (assessed by a linear model). [file 12934_2014_148_MOESM3_ESM.zip › Additional File 3A/llmg_0101_real_dat.png]

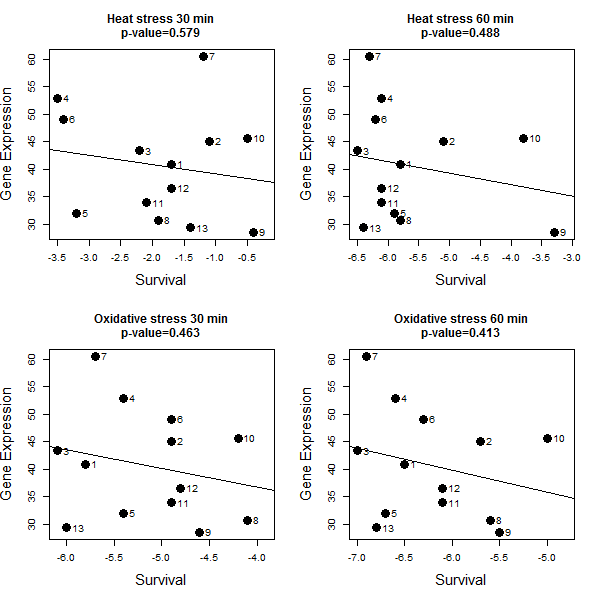

Supplement: Additional file 3: — Plots of gene expression and robustness levels. Expression levels of all genes plotted against survival after 30 and 60 minutes heat and oxidative stress (A: genes llmg_0001 to llmg_1229, B: genes llmg_1230 to llmg_2563). Survival is expressed as the difference of log CFU/ml after stress and before stress. Numbers indicate fermentations as presented in Table 1. P-values above the plots indicate significance of correlation (assessed by a linear model). [file 12934_2014_148_MOESM3_ESM.zip › Additional File 3A/llmg_0102_real_dat.png]

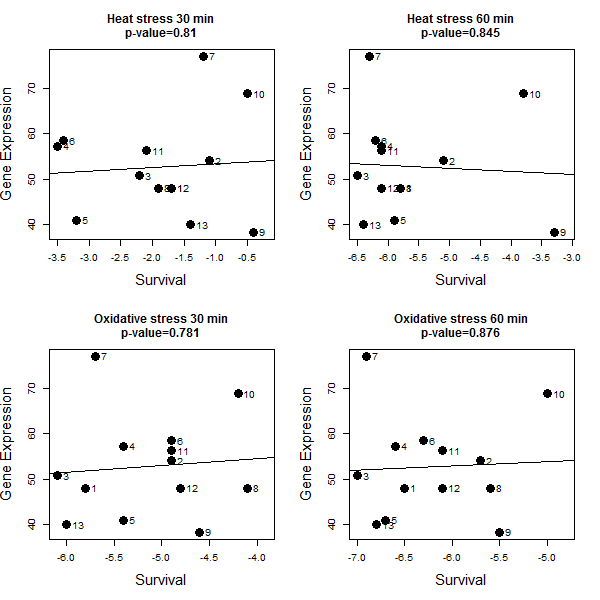

Supplement: Additional file 3: — Plots of gene expression and robustness levels. Expression levels of all genes plotted against survival after 30 and 60 minutes heat and oxidative stress (A: genes llmg_0001 to llmg_1229, B: genes llmg_1230 to llmg_2563). Survival is expressed as the difference of log CFU/ml after stress and before stress. Numbers indicate fermentations as presented in Table 1. P-values above the plots indicate significance of correlation (assessed by a linear model). [file 12934_2014_148_MOESM3_ESM.zip › Additional File 3A/llmg_0103_real_dat.png]

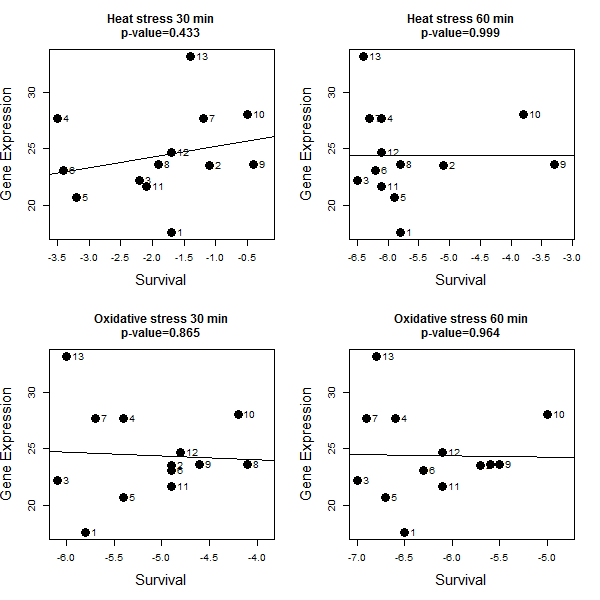

Supplement: Additional file 3: — Plots of gene expression and robustness levels. Expression levels of all genes plotted against survival after 30 and 60 minutes heat and oxidative stress (A: genes llmg_0001 to llmg_1229, B: genes llmg_1230 to llmg_2563). Survival is expressed as the difference of log CFU/ml after stress and before stress. Numbers indicate fermentations as presented in Table 1. P-values above the plots indicate significance of correlation (assessed by a linear model). [file 12934_2014_148_MOESM3_ESM.zip › Additional File 3A/llmg_0104_real_dat.png]

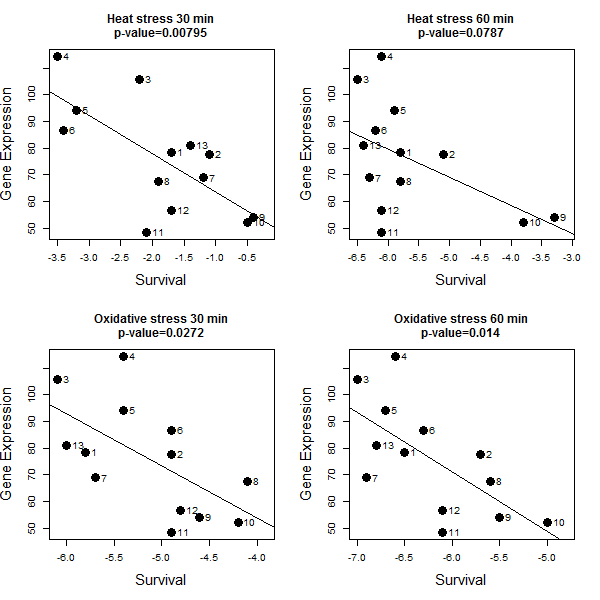

Supplement: Additional file 3: — Plots of gene expression and robustness levels. Expression levels of all genes plotted against survival after 30 and 60 minutes heat and oxidative stress (A: genes llmg_0001 to llmg_1229, B: genes llmg_1230 to llmg_2563). Survival is expressed as the difference of log CFU/ml after stress and before stress. Numbers indicate fermentations as presented in Table 1. P-values above the plots indicate significance of correlation (assessed by a linear model). [file 12934_2014_148_MOESM3_ESM.zip › Additional File 3A/llmg_0105_real_dat.png]

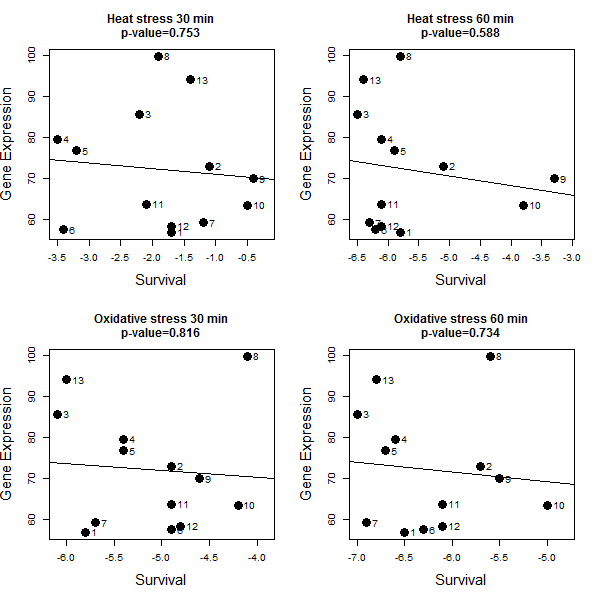

Supplement: Additional file 3: — Plots of gene expression and robustness levels. Expression levels of all genes plotted against survival after 30 and 60 minutes heat and oxidative stress (A: genes llmg_0001 to llmg_1229, B: genes llmg_1230 to llmg_2563). Survival is expressed as the difference of log CFU/ml after stress and before stress. Numbers indicate fermentations as presented in Table 1. P-values above the plots indicate significance of correlation (assessed by a linear model). [file 12934_2014_148_MOESM3_ESM.zip › Additional File 3A/llmg_0106_real_dat.png]
