# Supplementary figures and images for: Fermentation-induced variation in heat and oxidative stress phenotypes of Lactococcus lactis MG1363 reveals transcriptome signatures for robustness (part 3 of 3)
Source: Microb Cell Fact. 2014 Nov 4;13:148. doi: 10.1186/s12934-014-0148-6 (PMC4229599; doi:10.1186/s12934-014-0148-6)

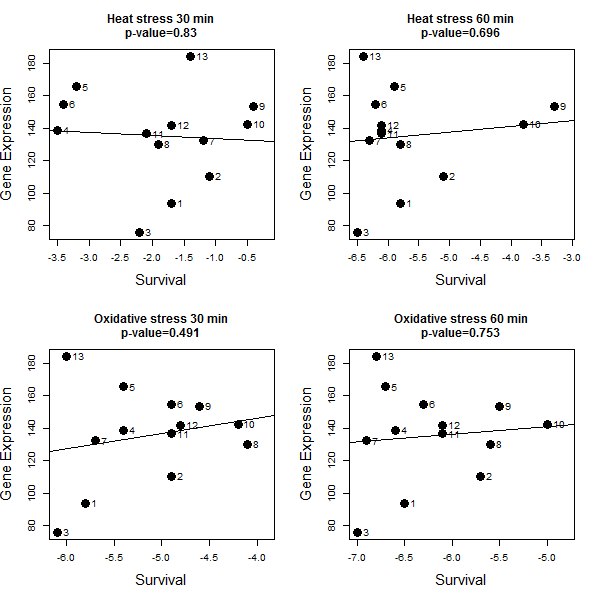

Supplement: Additional file 3: — Plots of gene expression and robustness levels. Expression levels of all genes plotted against survival after 30 and 60 minutes heat and oxidative stress (A: genes llmg_0001 to llmg_1229, B: genes llmg_1230 to llmg_2563). Survival is expressed as the difference of log CFU/ml after stress and before stress. Numbers indicate fermentations as presented in Table 1. P-values above the plots indicate significance of correlation (assessed by a linear model). [file 12934_2014_148_MOESM3_ESM.zip › Additional File 3A/llmg_0211_real_dat.png]

## Slide 1
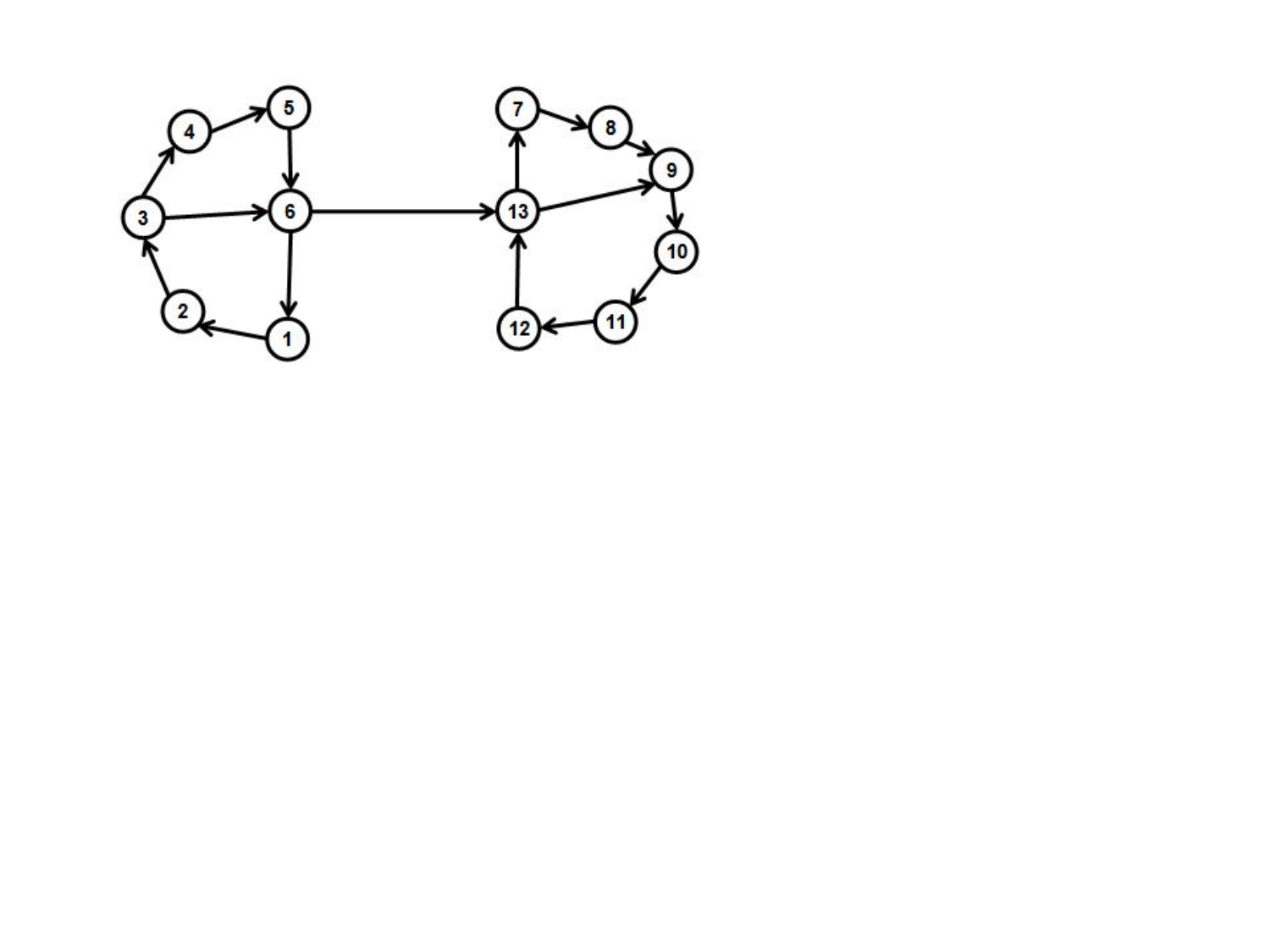

Supplement: Additional file 4: — DNA microarray hybridization scheme. Numbers indicate fermentations as presented in Table 1. Samples connected with arrows were hybridized together, the arrow head represents Cy5-labeling, the back end Cy3-labeling. [file 12934_2014_148_MOESM4_ESM.pptx]
