# Supplementary figures and images for: Fermentation-induced variation in heat and oxidative stress phenotypes of Lactococcus lactis MG1363 reveals transcriptome signatures for robustness (part 2 of 3)
Source: Microb Cell Fact. 2014 Nov 4;13:148. doi: 10.1186/s12934-014-0148-6 (PMC4229599; doi:10.1186/s12934-014-0148-6)

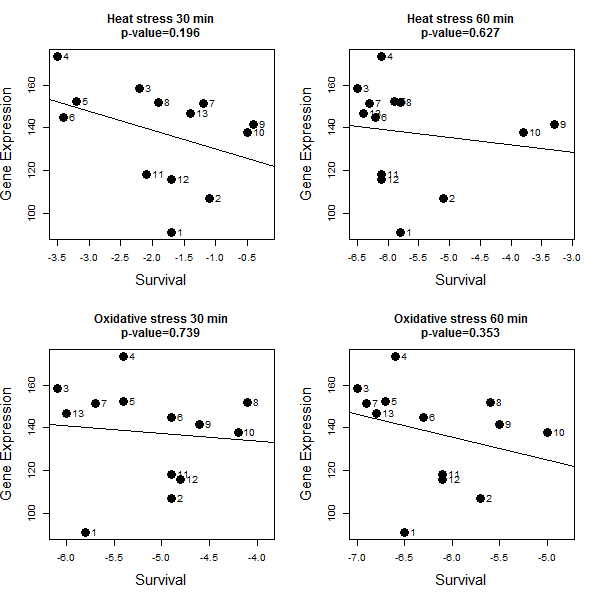

Supplement: Additional file 3: — Plots of gene expression and robustness levels. Expression levels of all genes plotted against survival after 30 and 60 minutes heat and oxidative stress (A: genes llmg_0001 to llmg_1229, B: genes llmg_1230 to llmg_2563). Survival is expressed as the difference of log CFU/ml after stress and before stress. Numbers indicate fermentations as presented in Table 1. P-values above the plots indicate significance of correlation (assessed by a linear model). [file 12934_2014_148_MOESM3_ESM.zip › Additional File 3A/llmg_0107_real_dat.png]

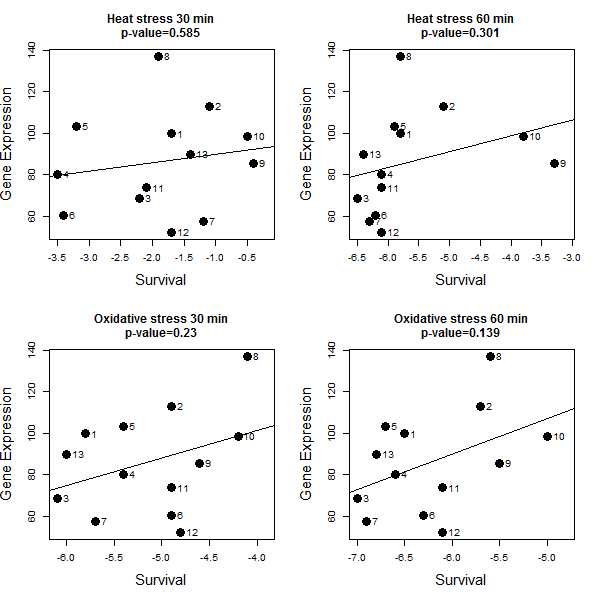

Supplement: Additional file 3: — Plots of gene expression and robustness levels. Expression levels of all genes plotted against survival after 30 and 60 minutes heat and oxidative stress (A: genes llmg_0001 to llmg_1229, B: genes llmg_1230 to llmg_2563). Survival is expressed as the difference of log CFU/ml after stress and before stress. Numbers indicate fermentations as presented in Table 1. P-values above the plots indicate significance of correlation (assessed by a linear model). [file 12934_2014_148_MOESM3_ESM.zip › Additional File 3A/llmg_0108_real_dat.png]

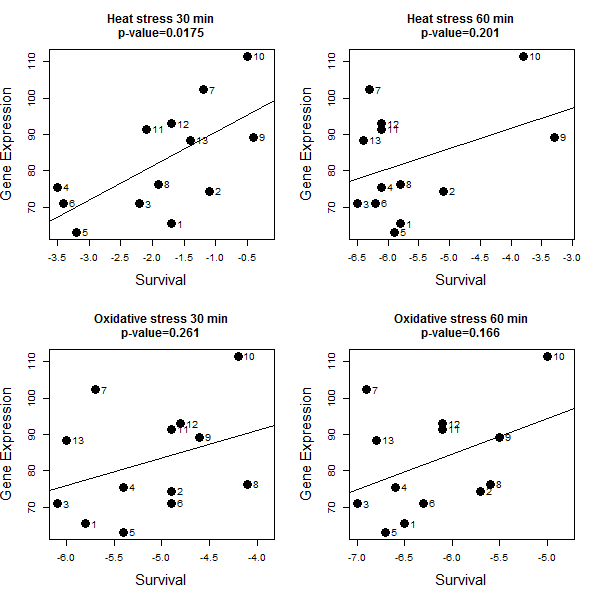

Supplement: Additional file 3: — Plots of gene expression and robustness levels. Expression levels of all genes plotted against survival after 30 and 60 minutes heat and oxidative stress (A: genes llmg_0001 to llmg_1229, B: genes llmg_1230 to llmg_2563). Survival is expressed as the difference of log CFU/ml after stress and before stress. Numbers indicate fermentations as presented in Table 1. P-values above the plots indicate significance of correlation (assessed by a linear model). [file 12934_2014_148_MOESM3_ESM.zip › Additional File 3A/llmg_0109_real_dat.png]

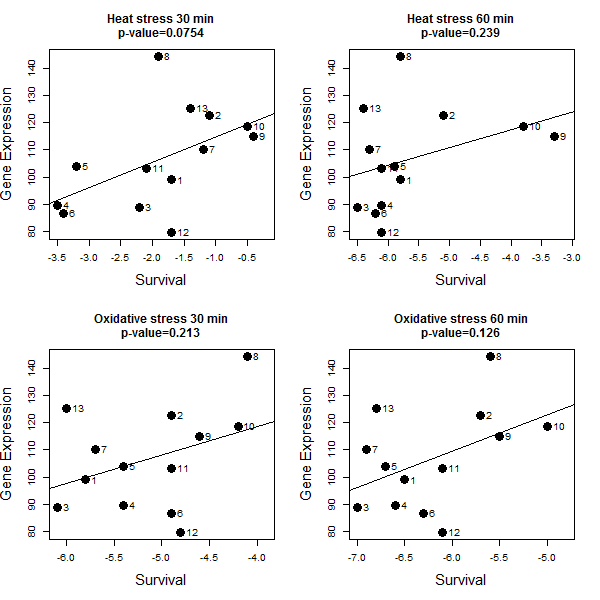

Supplement: Additional file 3: — Plots of gene expression and robustness levels. Expression levels of all genes plotted against survival after 30 and 60 minutes heat and oxidative stress (A: genes llmg_0001 to llmg_1229, B: genes llmg_1230 to llmg_2563). Survival is expressed as the difference of log CFU/ml after stress and before stress. Numbers indicate fermentations as presented in Table 1. P-values above the plots indicate significance of correlation (assessed by a linear model). [file 12934_2014_148_MOESM3_ESM.zip › Additional File 3A/llmg_0110_real_dat.png]

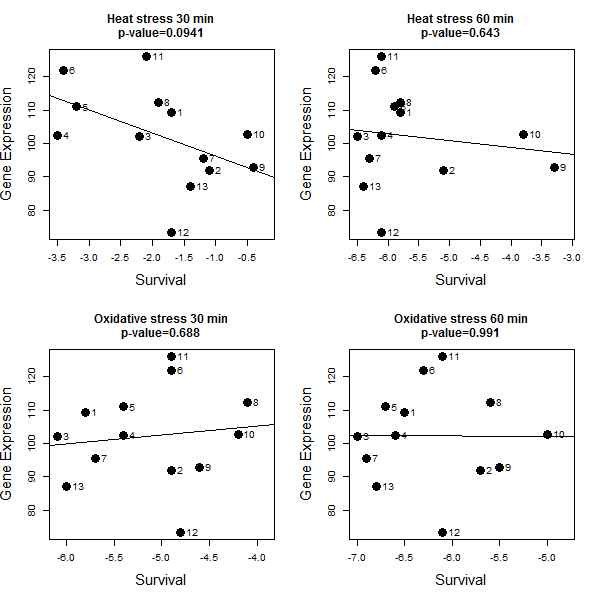

Supplement: Additional file 3: — Plots of gene expression and robustness levels. Expression levels of all genes plotted against survival after 30 and 60 minutes heat and oxidative stress (A: genes llmg_0001 to llmg_1229, B: genes llmg_1230 to llmg_2563). Survival is expressed as the difference of log CFU/ml after stress and before stress. Numbers indicate fermentations as presented in Table 1. P-values above the plots indicate significance of correlation (assessed by a linear model). [file 12934_2014_148_MOESM3_ESM.zip › Additional File 3A/llmg_0111_real_dat.png]

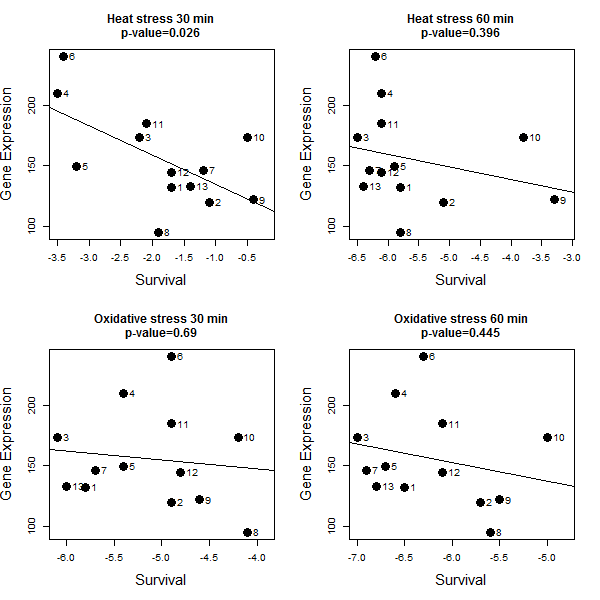

Supplement: Additional file 3: — Plots of gene expression and robustness levels. Expression levels of all genes plotted against survival after 30 and 60 minutes heat and oxidative stress (A: genes llmg_0001 to llmg_1229, B: genes llmg_1230 to llmg_2563). Survival is expressed as the difference of log CFU/ml after stress and before stress. Numbers indicate fermentations as presented in Table 1. P-values above the plots indicate significance of correlation (assessed by a linear model). [file 12934_2014_148_MOESM3_ESM.zip › Additional File 3A/llmg_0112_real_dat.png]

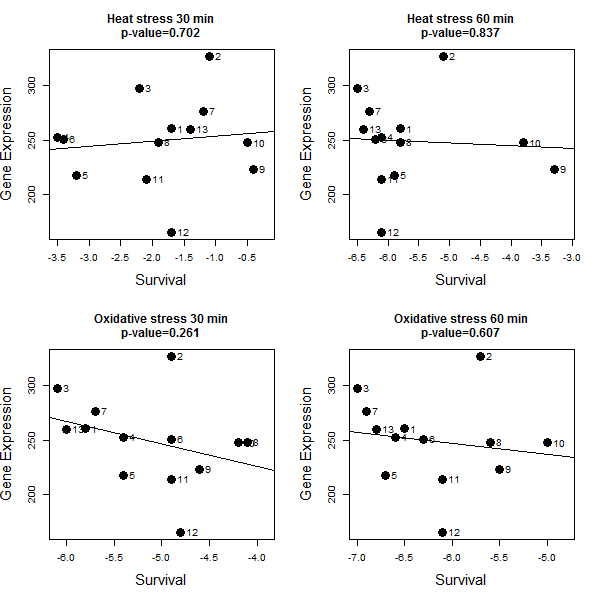

Supplement: Additional file 3: — Plots of gene expression and robustness levels. Expression levels of all genes plotted against survival after 30 and 60 minutes heat and oxidative stress (A: genes llmg_0001 to llmg_1229, B: genes llmg_1230 to llmg_2563). Survival is expressed as the difference of log CFU/ml after stress and before stress. Numbers indicate fermentations as presented in Table 1. P-values above the plots indicate significance of correlation (assessed by a linear model). [file 12934_2014_148_MOESM3_ESM.zip › Additional File 3A/llmg_0113_real_dat.png]

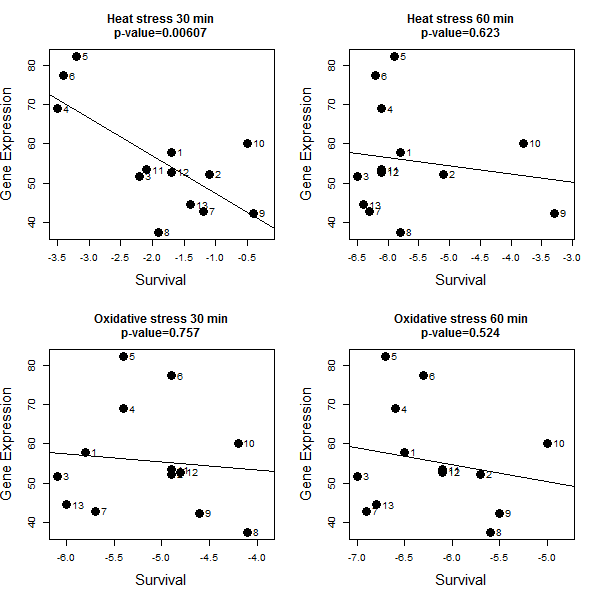

Supplement: Additional file 3: — Plots of gene expression and robustness levels. Expression levels of all genes plotted against survival after 30 and 60 minutes heat and oxidative stress (A: genes llmg_0001 to llmg_1229, B: genes llmg_1230 to llmg_2563). Survival is expressed as the difference of log CFU/ml after stress and before stress. Numbers indicate fermentations as presented in Table 1. P-values above the plots indicate significance of correlation (assessed by a linear model). [file 12934_2014_148_MOESM3_ESM.zip › Additional File 3A/llmg_0114_real_dat.png]

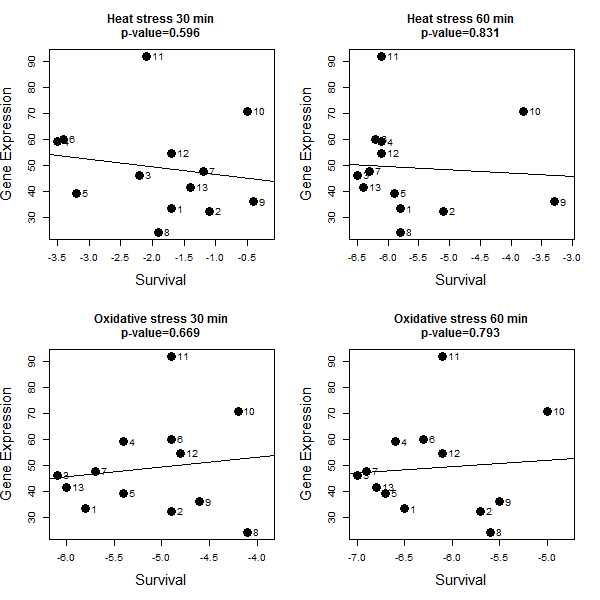

Supplement: Additional file 3: — Plots of gene expression and robustness levels. Expression levels of all genes plotted against survival after 30 and 60 minutes heat and oxidative stress (A: genes llmg_0001 to llmg_1229, B: genes llmg_1230 to llmg_2563). Survival is expressed as the difference of log CFU/ml after stress and before stress. Numbers indicate fermentations as presented in Table 1. P-values above the plots indicate significance of correlation (assessed by a linear model). [file 12934_2014_148_MOESM3_ESM.zip › Additional File 3A/llmg_0115_real_dat.png]

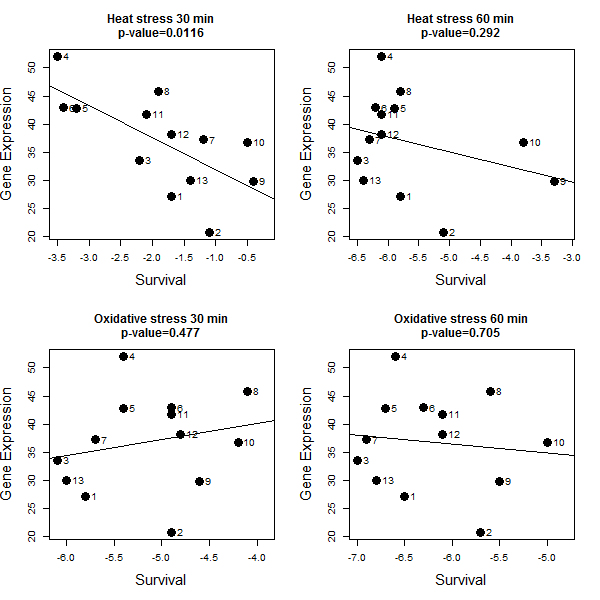

Supplement: Additional file 3: — Plots of gene expression and robustness levels. Expression levels of all genes plotted against survival after 30 and 60 minutes heat and oxidative stress (A: genes llmg_0001 to llmg_1229, B: genes llmg_1230 to llmg_2563). Survival is expressed as the difference of log CFU/ml after stress and before stress. Numbers indicate fermentations as presented in Table 1. P-values above the plots indicate significance of correlation (assessed by a linear model). [file 12934_2014_148_MOESM3_ESM.zip › Additional File 3A/llmg_0116_real_dat.png]

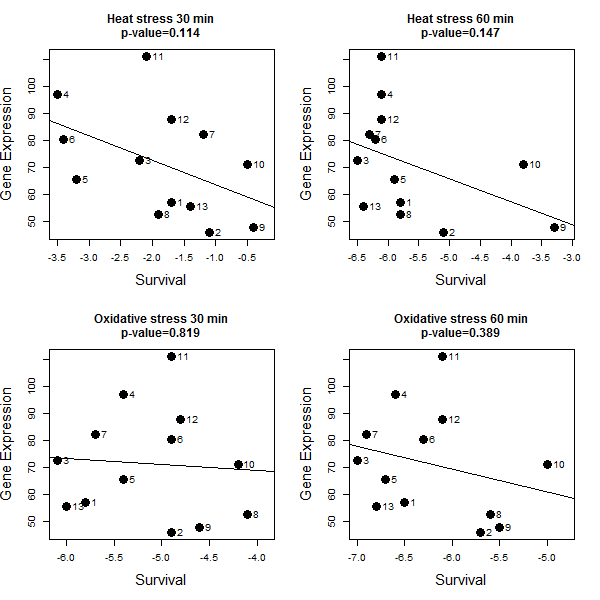

Supplement: Additional file 3: — Plots of gene expression and robustness levels. Expression levels of all genes plotted against survival after 30 and 60 minutes heat and oxidative stress (A: genes llmg_0001 to llmg_1229, B: genes llmg_1230 to llmg_2563). Survival is expressed as the difference of log CFU/ml after stress and before stress. Numbers indicate fermentations as presented in Table 1. P-values above the plots indicate significance of correlation (assessed by a linear model). [file 12934_2014_148_MOESM3_ESM.zip › Additional File 3A/llmg_0117_real_dat.png]

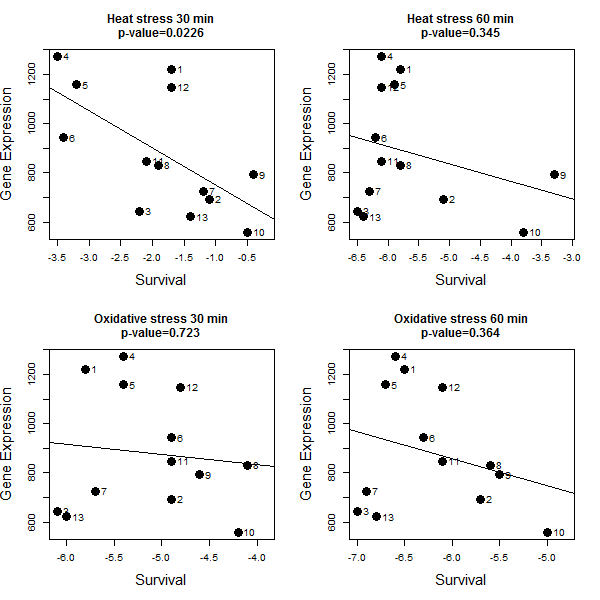

Supplement: Additional file 3: — Plots of gene expression and robustness levels. Expression levels of all genes plotted against survival after 30 and 60 minutes heat and oxidative stress (A: genes llmg_0001 to llmg_1229, B: genes llmg_1230 to llmg_2563). Survival is expressed as the difference of log CFU/ml after stress and before stress. Numbers indicate fermentations as presented in Table 1. P-values above the plots indicate significance of correlation (assessed by a linear model). [file 12934_2014_148_MOESM3_ESM.zip › Additional File 3A/llmg_0118_real_dat.png]

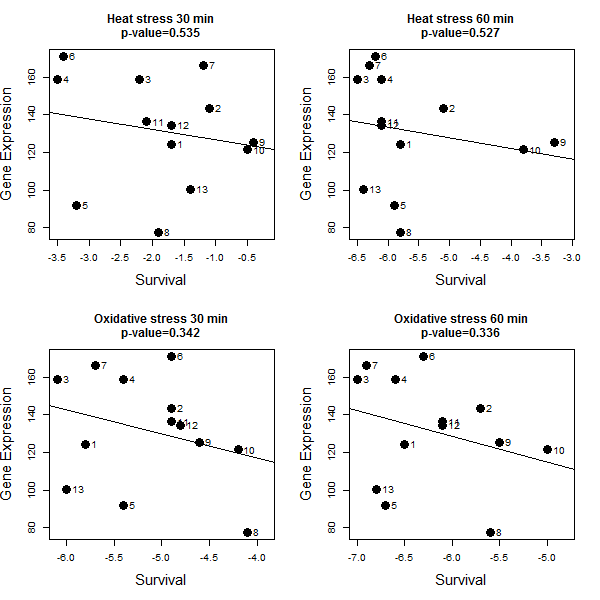

Supplement: Additional file 3: — Plots of gene expression and robustness levels. Expression levels of all genes plotted against survival after 30 and 60 minutes heat and oxidative stress (A: genes llmg_0001 to llmg_1229, B: genes llmg_1230 to llmg_2563). Survival is expressed as the difference of log CFU/ml after stress and before stress. Numbers indicate fermentations as presented in Table 1. P-values above the plots indicate significance of correlation (assessed by a linear model). [file 12934_2014_148_MOESM3_ESM.zip › Additional File 3A/llmg_0119_real_dat.png]

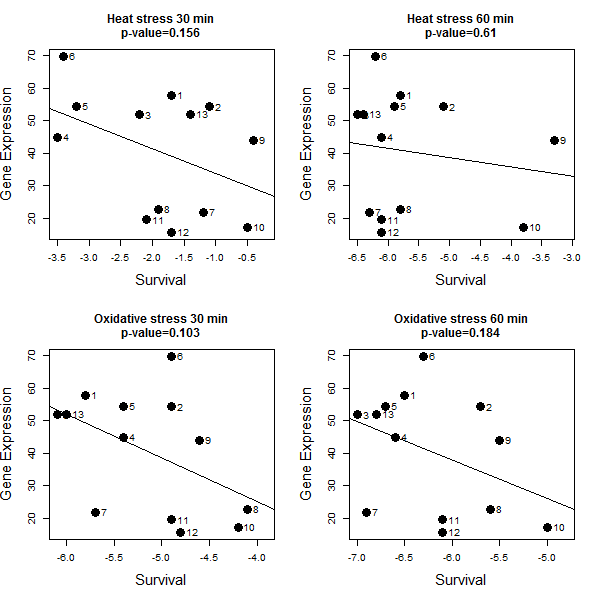

Supplement: Additional file 3: — Plots of gene expression and robustness levels. Expression levels of all genes plotted against survival after 30 and 60 minutes heat and oxidative stress (A: genes llmg_0001 to llmg_1229, B: genes llmg_1230 to llmg_2563). Survival is expressed as the difference of log CFU/ml after stress and before stress. Numbers indicate fermentations as presented in Table 1. P-values above the plots indicate significance of correlation (assessed by a linear model). [file 12934_2014_148_MOESM3_ESM.zip › Additional File 3A/llmg_0120_real_dat.png]

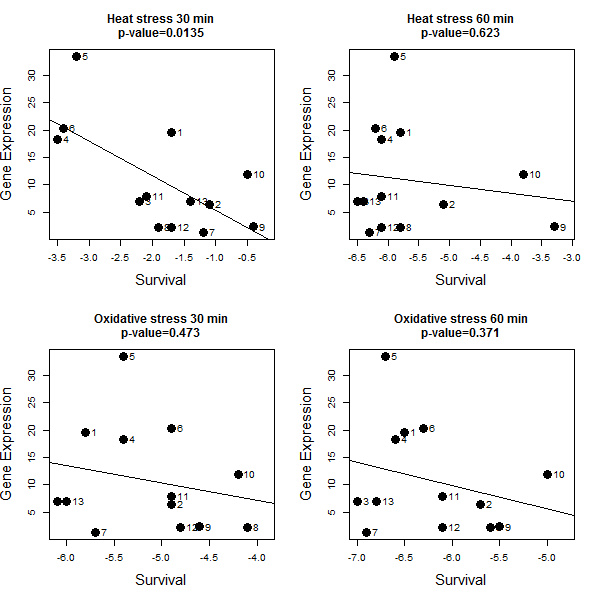

Supplement: Additional file 3: — Plots of gene expression and robustness levels. Expression levels of all genes plotted against survival after 30 and 60 minutes heat and oxidative stress (A: genes llmg_0001 to llmg_1229, B: genes llmg_1230 to llmg_2563). Survival is expressed as the difference of log CFU/ml after stress and before stress. Numbers indicate fermentations as presented in Table 1. P-values above the plots indicate significance of correlation (assessed by a linear model). [file 12934_2014_148_MOESM3_ESM.zip › Additional File 3A/llmg_0123_real_dat.png]

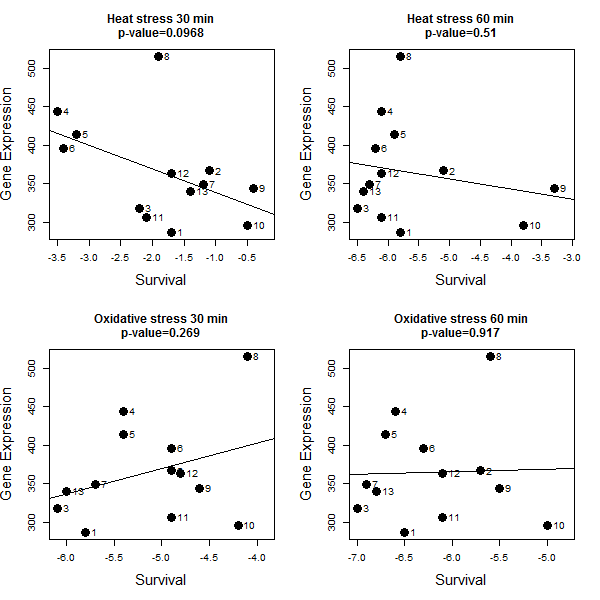

Supplement: Additional file 3: — Plots of gene expression and robustness levels. Expression levels of all genes plotted against survival after 30 and 60 minutes heat and oxidative stress (A: genes llmg_0001 to llmg_1229, B: genes llmg_1230 to llmg_2563). Survival is expressed as the difference of log CFU/ml after stress and before stress. Numbers indicate fermentations as presented in Table 1. P-values above the plots indicate significance of correlation (assessed by a linear model). [file 12934_2014_148_MOESM3_ESM.zip › Additional File 3A/llmg_0124_real_dat.png]

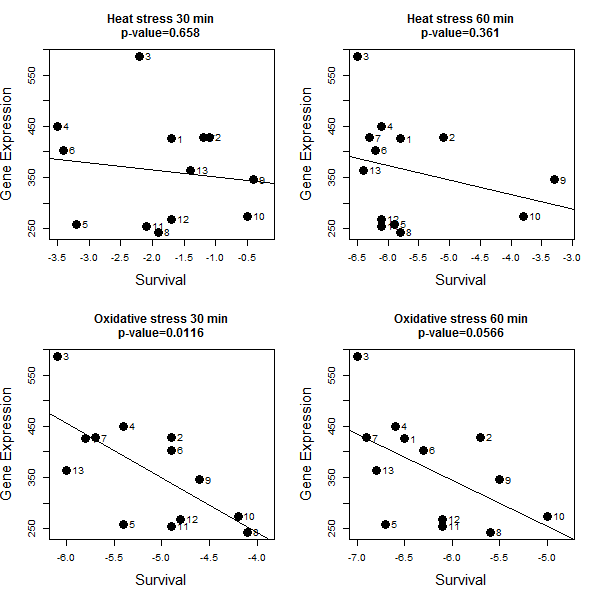

Supplement: Additional file 3: — Plots of gene expression and robustness levels. Expression levels of all genes plotted against survival after 30 and 60 minutes heat and oxidative stress (A: genes llmg_0001 to llmg_1229, B: genes llmg_1230 to llmg_2563). Survival is expressed as the difference of log CFU/ml after stress and before stress. Numbers indicate fermentations as presented in Table 1. P-values above the plots indicate significance of correlation (assessed by a linear model). [file 12934_2014_148_MOESM3_ESM.zip › Additional File 3A/llmg_0125_real_dat.png]

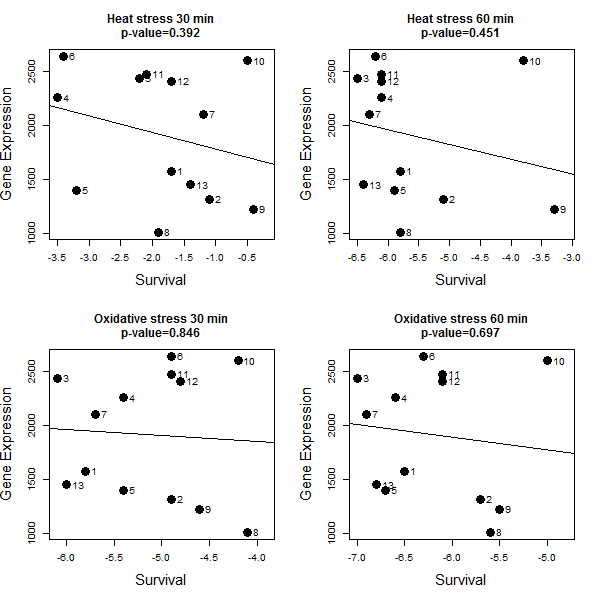

Supplement: Additional file 3: — Plots of gene expression and robustness levels. Expression levels of all genes plotted against survival after 30 and 60 minutes heat and oxidative stress (A: genes llmg_0001 to llmg_1229, B: genes llmg_1230 to llmg_2563). Survival is expressed as the difference of log CFU/ml after stress and before stress. Numbers indicate fermentations as presented in Table 1. P-values above the plots indicate significance of correlation (assessed by a linear model). [file 12934_2014_148_MOESM3_ESM.zip › Additional File 3A/llmg_0126_real_dat.png]

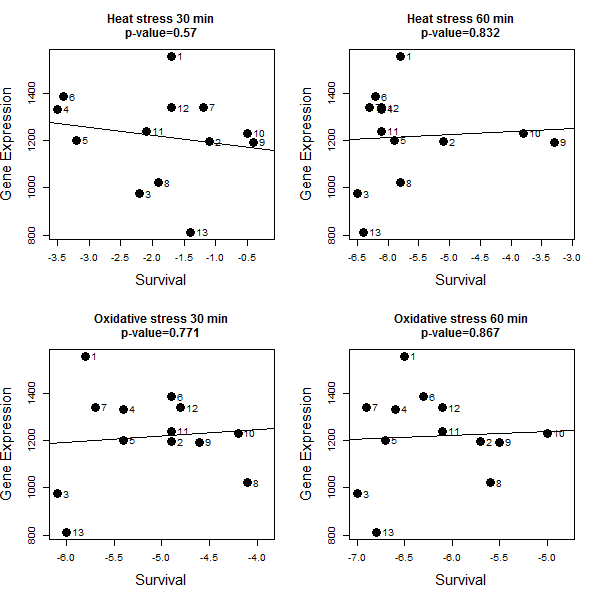

Supplement: Additional file 3: — Plots of gene expression and robustness levels. Expression levels of all genes plotted against survival after 30 and 60 minutes heat and oxidative stress (A: genes llmg_0001 to llmg_1229, B: genes llmg_1230 to llmg_2563). Survival is expressed as the difference of log CFU/ml after stress and before stress. Numbers indicate fermentations as presented in Table 1. P-values above the plots indicate significance of correlation (assessed by a linear model). [file 12934_2014_148_MOESM3_ESM.zip › Additional File 3A/llmg_0127_real_dat.png]

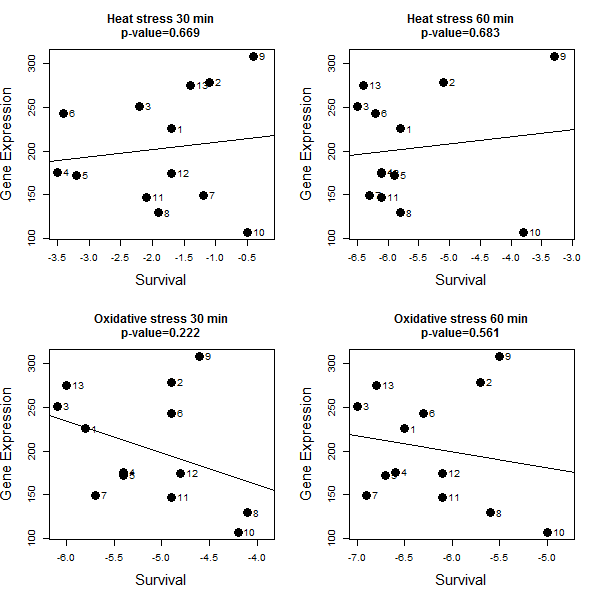

Supplement: Additional file 3: — Plots of gene expression and robustness levels. Expression levels of all genes plotted against survival after 30 and 60 minutes heat and oxidative stress (A: genes llmg_0001 to llmg_1229, B: genes llmg_1230 to llmg_2563). Survival is expressed as the difference of log CFU/ml after stress and before stress. Numbers indicate fermentations as presented in Table 1. P-values above the plots indicate significance of correlation (assessed by a linear model). [file 12934_2014_148_MOESM3_ESM.zip › Additional File 3A/llmg_0128_real_dat.png]

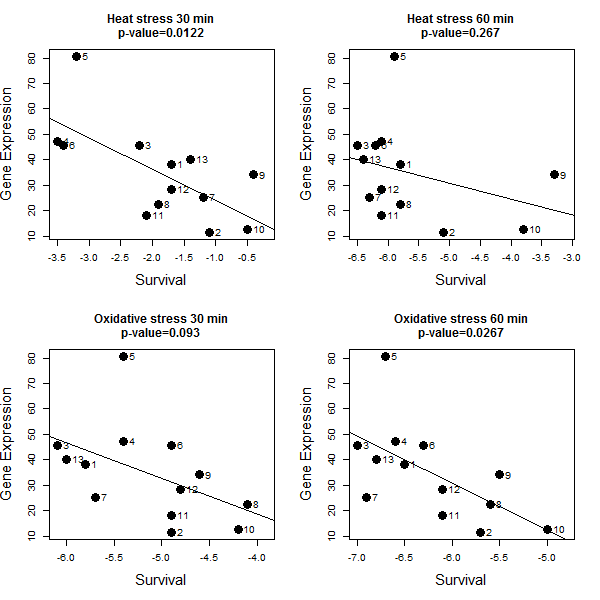

Supplement: Additional file 3: — Plots of gene expression and robustness levels. Expression levels of all genes plotted against survival after 30 and 60 minutes heat and oxidative stress (A: genes llmg_0001 to llmg_1229, B: genes llmg_1230 to llmg_2563). Survival is expressed as the difference of log CFU/ml after stress and before stress. Numbers indicate fermentations as presented in Table 1. P-values above the plots indicate significance of correlation (assessed by a linear model). [file 12934_2014_148_MOESM3_ESM.zip › Additional File 3A/llmg_0129_real_dat.png]

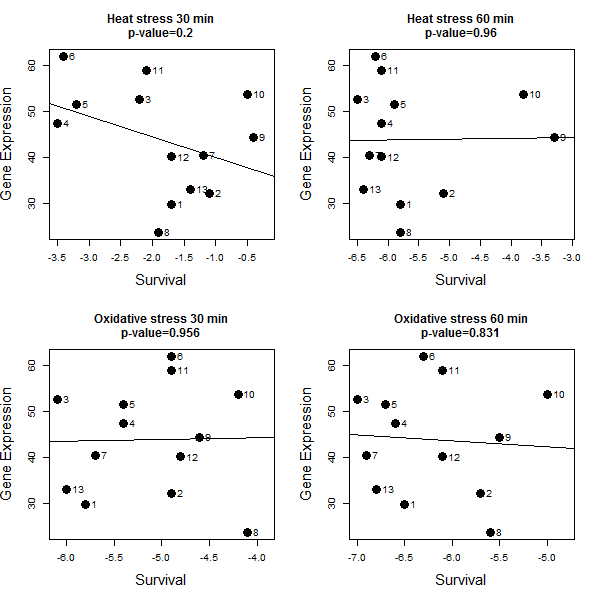

Supplement: Additional file 3: — Plots of gene expression and robustness levels. Expression levels of all genes plotted against survival after 30 and 60 minutes heat and oxidative stress (A: genes llmg_0001 to llmg_1229, B: genes llmg_1230 to llmg_2563). Survival is expressed as the difference of log CFU/ml after stress and before stress. Numbers indicate fermentations as presented in Table 1. P-values above the plots indicate significance of correlation (assessed by a linear model). [file 12934_2014_148_MOESM3_ESM.zip › Additional File 3A/llmg_0130_real_dat.png]

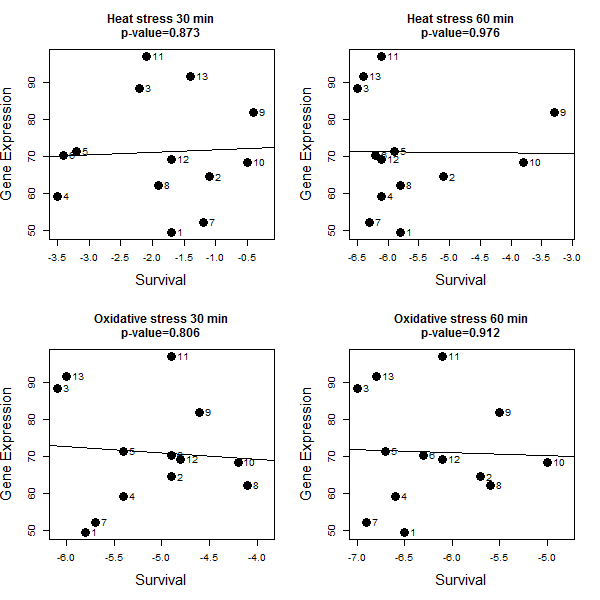

Supplement: Additional file 3: — Plots of gene expression and robustness levels. Expression levels of all genes plotted against survival after 30 and 60 minutes heat and oxidative stress (A: genes llmg_0001 to llmg_1229, B: genes llmg_1230 to llmg_2563). Survival is expressed as the difference of log CFU/ml after stress and before stress. Numbers indicate fermentations as presented in Table 1. P-values above the plots indicate significance of correlation (assessed by a linear model). [file 12934_2014_148_MOESM3_ESM.zip › Additional File 3A/llmg_0131_real_dat.png]

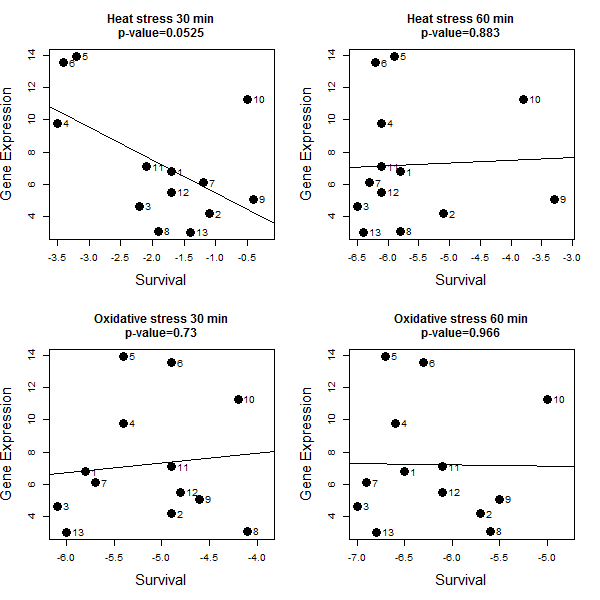

Supplement: Additional file 3: — Plots of gene expression and robustness levels. Expression levels of all genes plotted against survival after 30 and 60 minutes heat and oxidative stress (A: genes llmg_0001 to llmg_1229, B: genes llmg_1230 to llmg_2563). Survival is expressed as the difference of log CFU/ml after stress and before stress. Numbers indicate fermentations as presented in Table 1. P-values above the plots indicate significance of correlation (assessed by a linear model). [file 12934_2014_148_MOESM3_ESM.zip › Additional File 3A/llmg_0132_real_dat.png]

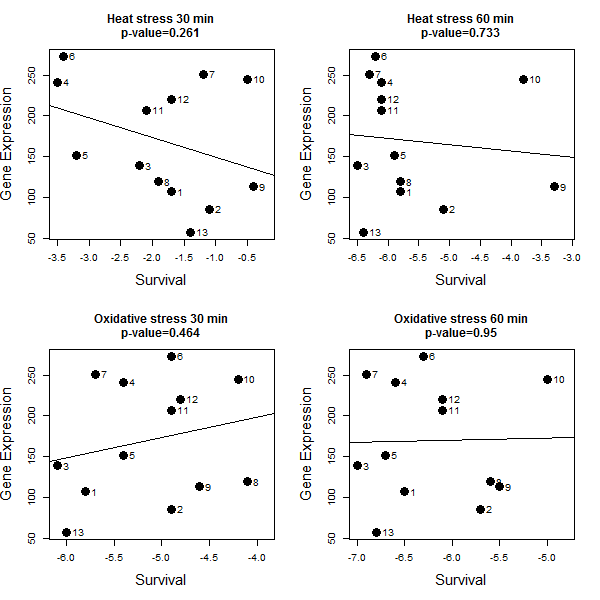

Supplement: Additional file 3: — Plots of gene expression and robustness levels. Expression levels of all genes plotted against survival after 30 and 60 minutes heat and oxidative stress (A: genes llmg_0001 to llmg_1229, B: genes llmg_1230 to llmg_2563). Survival is expressed as the difference of log CFU/ml after stress and before stress. Numbers indicate fermentations as presented in Table 1. P-values above the plots indicate significance of correlation (assessed by a linear model). [file 12934_2014_148_MOESM3_ESM.zip › Additional File 3A/llmg_0133_real_dat.png]

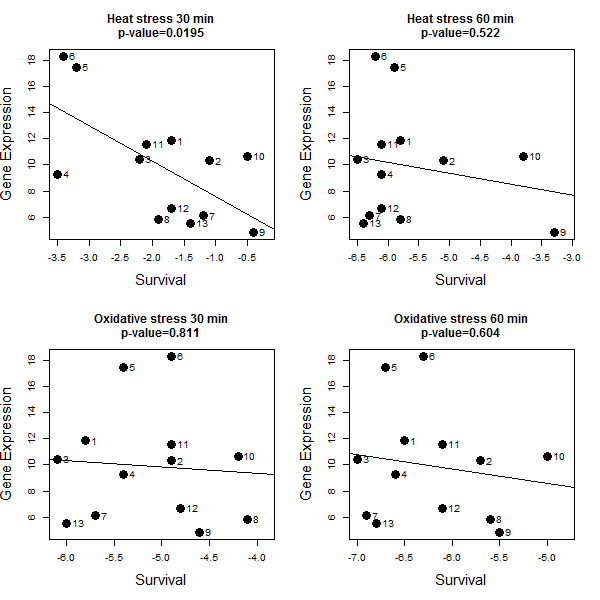

Supplement: Additional file 3: — Plots of gene expression and robustness levels. Expression levels of all genes plotted against survival after 30 and 60 minutes heat and oxidative stress (A: genes llmg_0001 to llmg_1229, B: genes llmg_1230 to llmg_2563). Survival is expressed as the difference of log CFU/ml after stress and before stress. Numbers indicate fermentations as presented in Table 1. P-values above the plots indicate significance of correlation (assessed by a linear model). [file 12934_2014_148_MOESM3_ESM.zip › Additional File 3A/llmg_0134_real_dat.png]

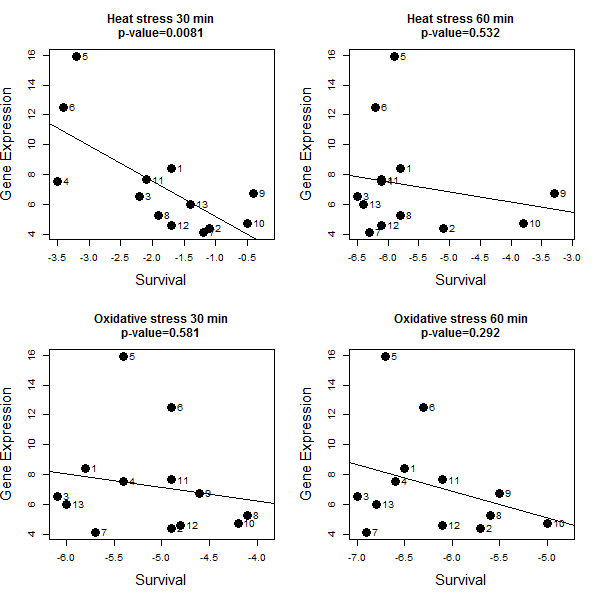

Supplement: Additional file 3: — Plots of gene expression and robustness levels. Expression levels of all genes plotted against survival after 30 and 60 minutes heat and oxidative stress (A: genes llmg_0001 to llmg_1229, B: genes llmg_1230 to llmg_2563). Survival is expressed as the difference of log CFU/ml after stress and before stress. Numbers indicate fermentations as presented in Table 1. P-values above the plots indicate significance of correlation (assessed by a linear model). [file 12934_2014_148_MOESM3_ESM.zip › Additional File 3A/llmg_0135_real_dat.png]

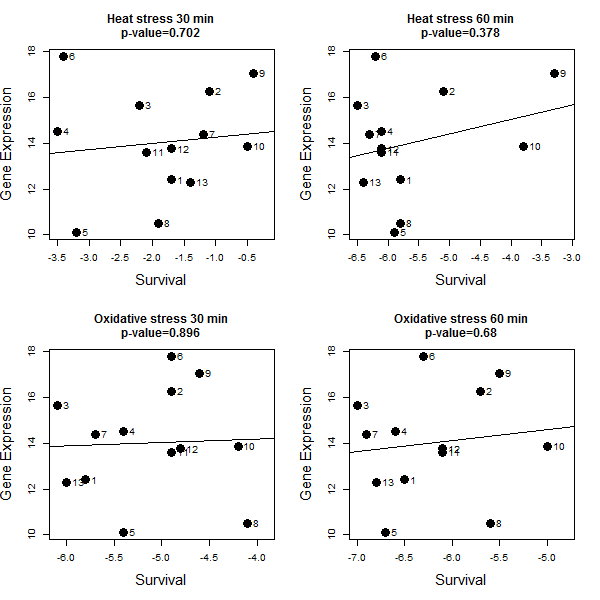

Supplement: Additional file 3: — Plots of gene expression and robustness levels. Expression levels of all genes plotted against survival after 30 and 60 minutes heat and oxidative stress (A: genes llmg_0001 to llmg_1229, B: genes llmg_1230 to llmg_2563). Survival is expressed as the difference of log CFU/ml after stress and before stress. Numbers indicate fermentations as presented in Table 1. P-values above the plots indicate significance of correlation (assessed by a linear model). [file 12934_2014_148_MOESM3_ESM.zip › Additional File 3A/llmg_0136_real_dat.png]

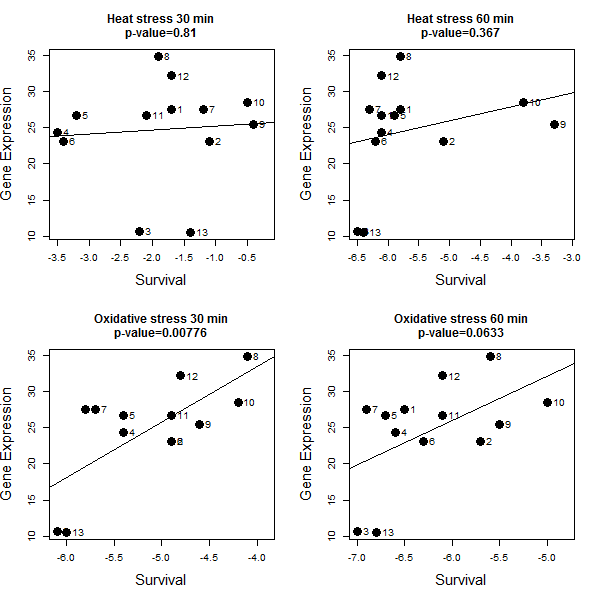

Supplement: Additional file 3: — Plots of gene expression and robustness levels. Expression levels of all genes plotted against survival after 30 and 60 minutes heat and oxidative stress (A: genes llmg_0001 to llmg_1229, B: genes llmg_1230 to llmg_2563). Survival is expressed as the difference of log CFU/ml after stress and before stress. Numbers indicate fermentations as presented in Table 1. P-values above the plots indicate significance of correlation (assessed by a linear model). [file 12934_2014_148_MOESM3_ESM.zip › Additional File 3A/llmg_0137_real_dat.png]

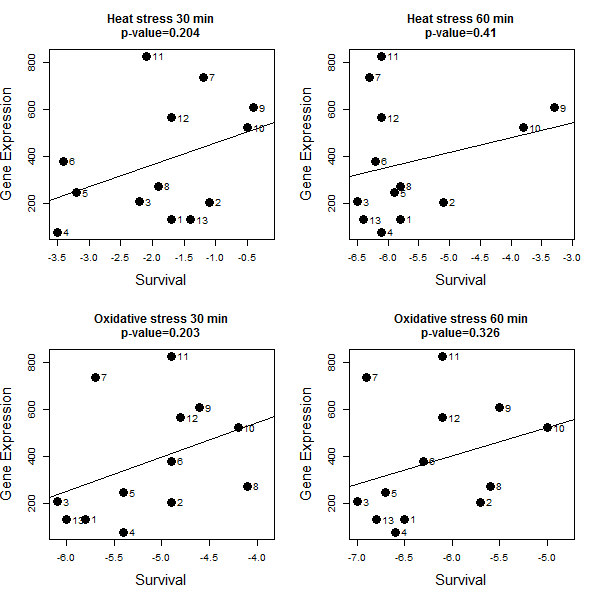

Supplement: Additional file 3: — Plots of gene expression and robustness levels. Expression levels of all genes plotted against survival after 30 and 60 minutes heat and oxidative stress (A: genes llmg_0001 to llmg_1229, B: genes llmg_1230 to llmg_2563). Survival is expressed as the difference of log CFU/ml after stress and before stress. Numbers indicate fermentations as presented in Table 1. P-values above the plots indicate significance of correlation (assessed by a linear model). [file 12934_2014_148_MOESM3_ESM.zip › Additional File 3A/llmg_0138_real_dat.png]

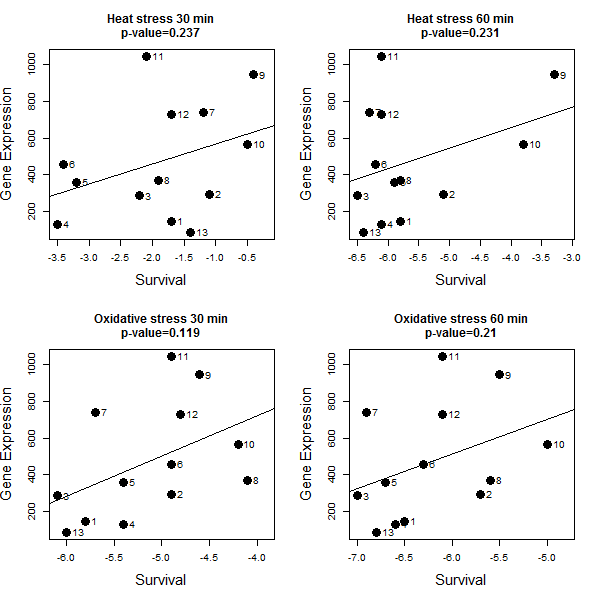

Supplement: Additional file 3: — Plots of gene expression and robustness levels. Expression levels of all genes plotted against survival after 30 and 60 minutes heat and oxidative stress (A: genes llmg_0001 to llmg_1229, B: genes llmg_1230 to llmg_2563). Survival is expressed as the difference of log CFU/ml after stress and before stress. Numbers indicate fermentations as presented in Table 1. P-values above the plots indicate significance of correlation (assessed by a linear model). [file 12934_2014_148_MOESM3_ESM.zip › Additional File 3A/llmg_0139_real_dat.png]

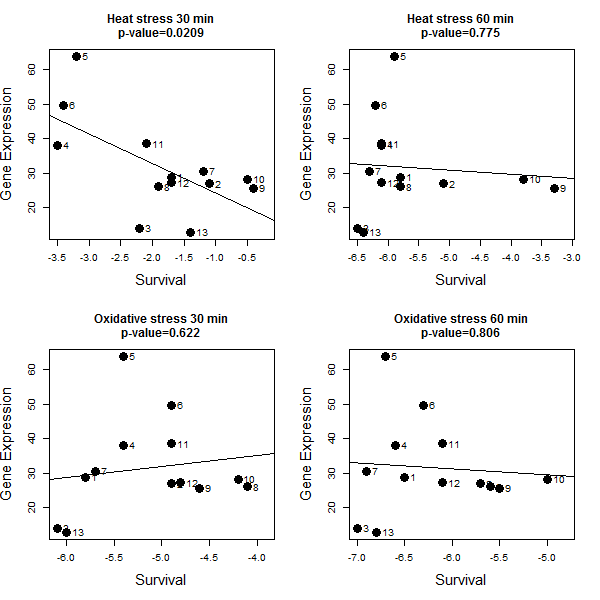

Supplement: Additional file 3: — Plots of gene expression and robustness levels. Expression levels of all genes plotted against survival after 30 and 60 minutes heat and oxidative stress (A: genes llmg_0001 to llmg_1229, B: genes llmg_1230 to llmg_2563). Survival is expressed as the difference of log CFU/ml after stress and before stress. Numbers indicate fermentations as presented in Table 1. P-values above the plots indicate significance of correlation (assessed by a linear model). [file 12934_2014_148_MOESM3_ESM.zip › Additional File 3A/llmg_0140_real_dat.png]

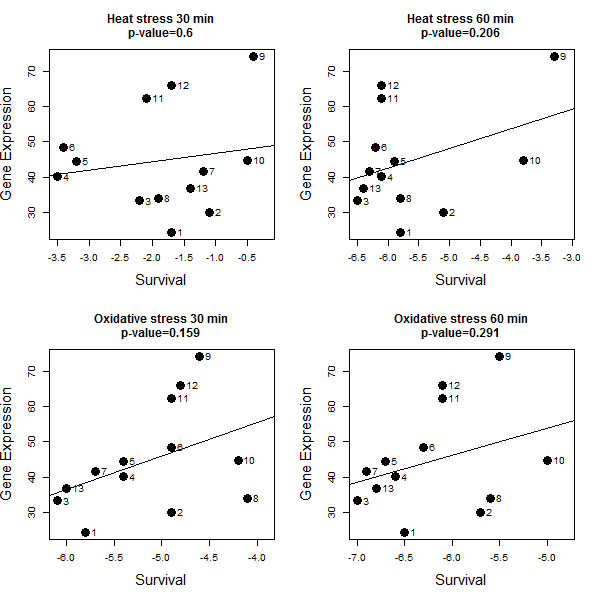

Supplement: Additional file 3: — Plots of gene expression and robustness levels. Expression levels of all genes plotted against survival after 30 and 60 minutes heat and oxidative stress (A: genes llmg_0001 to llmg_1229, B: genes llmg_1230 to llmg_2563). Survival is expressed as the difference of log CFU/ml after stress and before stress. Numbers indicate fermentations as presented in Table 1. P-values above the plots indicate significance of correlation (assessed by a linear model). [file 12934_2014_148_MOESM3_ESM.zip › Additional File 3A/llmg_0141_real_dat.png]

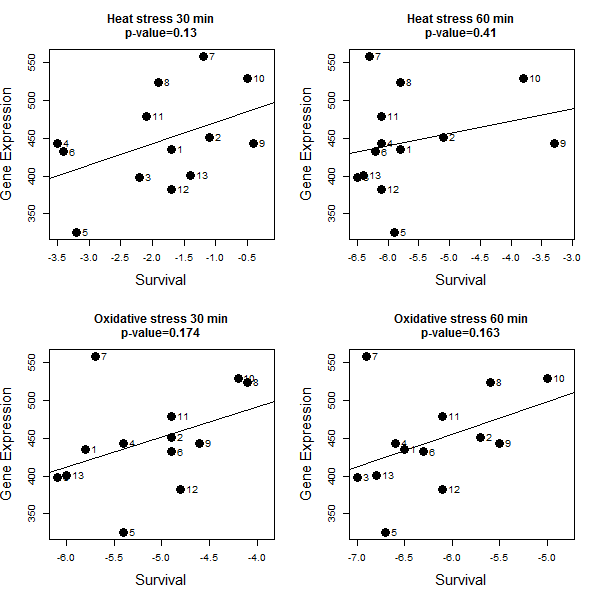

Supplement: Additional file 3: — Plots of gene expression and robustness levels. Expression levels of all genes plotted against survival after 30 and 60 minutes heat and oxidative stress (A: genes llmg_0001 to llmg_1229, B: genes llmg_1230 to llmg_2563). Survival is expressed as the difference of log CFU/ml after stress and before stress. Numbers indicate fermentations as presented in Table 1. P-values above the plots indicate significance of correlation (assessed by a linear model). [file 12934_2014_148_MOESM3_ESM.zip › Additional File 3A/llmg_0142_real_dat.png]

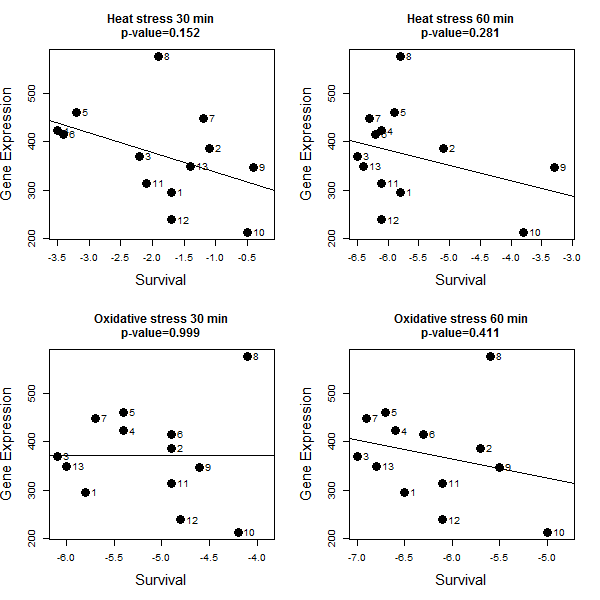

Supplement: Additional file 3: — Plots of gene expression and robustness levels. Expression levels of all genes plotted against survival after 30 and 60 minutes heat and oxidative stress (A: genes llmg_0001 to llmg_1229, B: genes llmg_1230 to llmg_2563). Survival is expressed as the difference of log CFU/ml after stress and before stress. Numbers indicate fermentations as presented in Table 1. P-values above the plots indicate significance of correlation (assessed by a linear model). [file 12934_2014_148_MOESM3_ESM.zip › Additional File 3A/llmg_0143_real_dat.png]

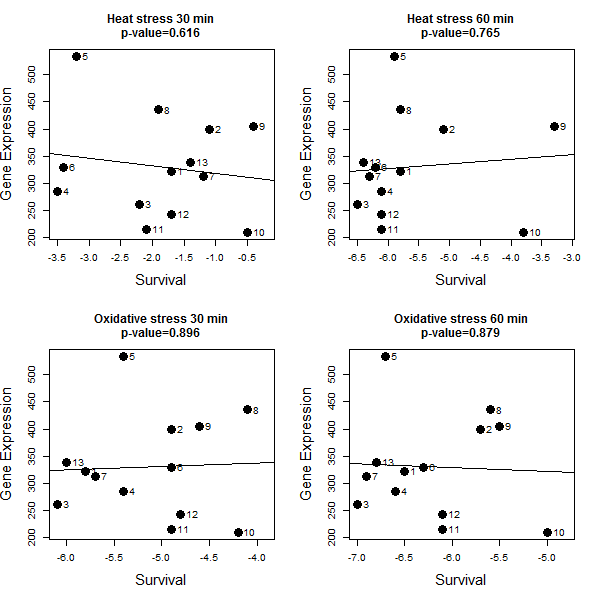

Supplement: Additional file 3: — Plots of gene expression and robustness levels. Expression levels of all genes plotted against survival after 30 and 60 minutes heat and oxidative stress (A: genes llmg_0001 to llmg_1229, B: genes llmg_1230 to llmg_2563). Survival is expressed as the difference of log CFU/ml after stress and before stress. Numbers indicate fermentations as presented in Table 1. P-values above the plots indicate significance of correlation (assessed by a linear model). [file 12934_2014_148_MOESM3_ESM.zip › Additional File 3A/llmg_0144_real_dat.png]

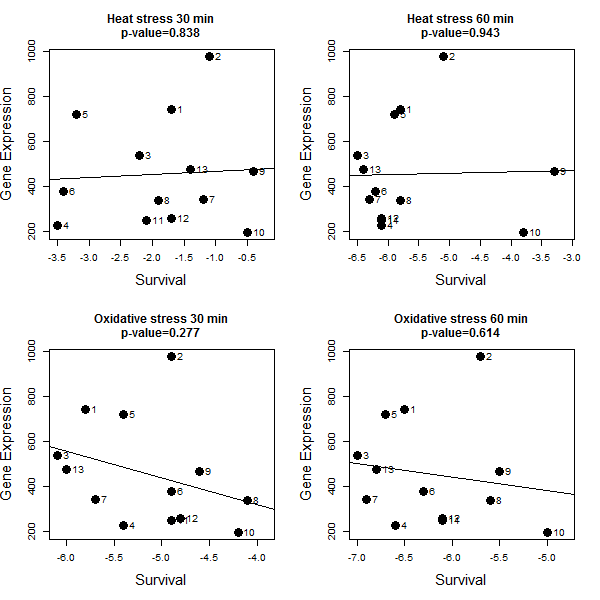

Supplement: Additional file 3: — Plots of gene expression and robustness levels. Expression levels of all genes plotted against survival after 30 and 60 minutes heat and oxidative stress (A: genes llmg_0001 to llmg_1229, B: genes llmg_1230 to llmg_2563). Survival is expressed as the difference of log CFU/ml after stress and before stress. Numbers indicate fermentations as presented in Table 1. P-values above the plots indicate significance of correlation (assessed by a linear model). [file 12934_2014_148_MOESM3_ESM.zip › Additional File 3A/llmg_0145_real_dat.png]

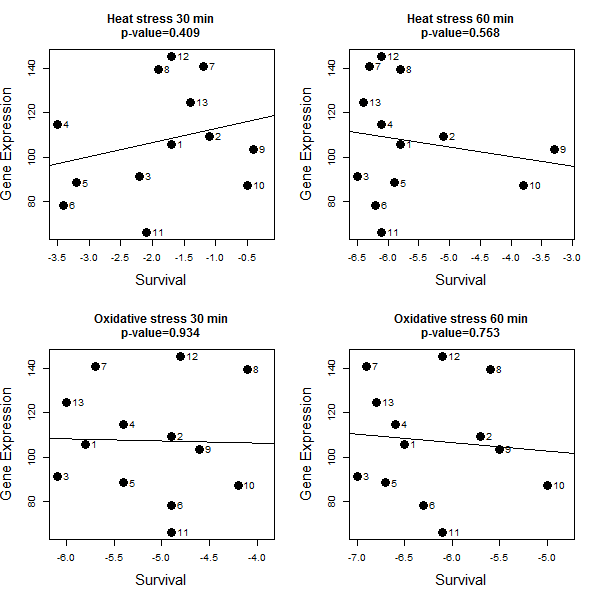

Supplement: Additional file 3: — Plots of gene expression and robustness levels. Expression levels of all genes plotted against survival after 30 and 60 minutes heat and oxidative stress (A: genes llmg_0001 to llmg_1229, B: genes llmg_1230 to llmg_2563). Survival is expressed as the difference of log CFU/ml after stress and before stress. Numbers indicate fermentations as presented in Table 1. P-values above the plots indicate significance of correlation (assessed by a linear model). [file 12934_2014_148_MOESM3_ESM.zip › Additional File 3A/llmg_0146_real_dat.png]

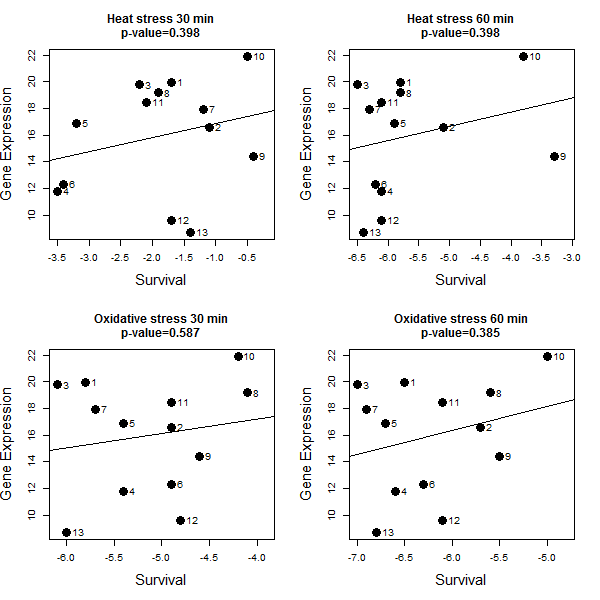

Supplement: Additional file 3: — Plots of gene expression and robustness levels. Expression levels of all genes plotted against survival after 30 and 60 minutes heat and oxidative stress (A: genes llmg_0001 to llmg_1229, B: genes llmg_1230 to llmg_2563). Survival is expressed as the difference of log CFU/ml after stress and before stress. Numbers indicate fermentations as presented in Table 1. P-values above the plots indicate significance of correlation (assessed by a linear model). [file 12934_2014_148_MOESM3_ESM.zip › Additional File 3A/llmg_0147_real_dat.png]

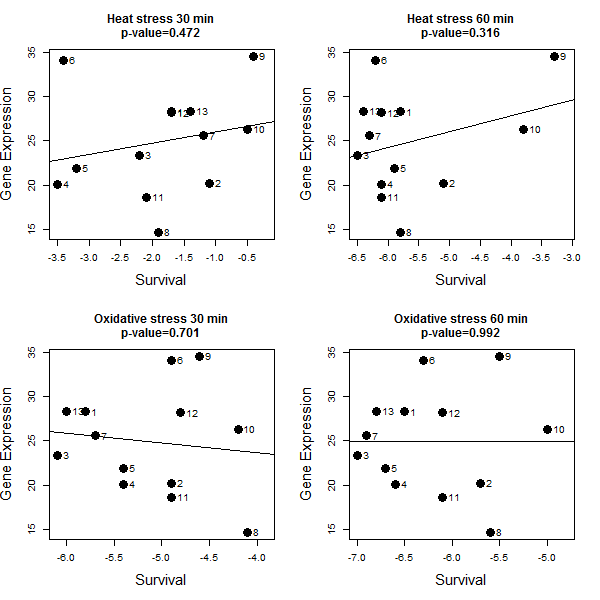

Supplement: Additional file 3: — Plots of gene expression and robustness levels. Expression levels of all genes plotted against survival after 30 and 60 minutes heat and oxidative stress (A: genes llmg_0001 to llmg_1229, B: genes llmg_1230 to llmg_2563). Survival is expressed as the difference of log CFU/ml after stress and before stress. Numbers indicate fermentations as presented in Table 1. P-values above the plots indicate significance of correlation (assessed by a linear model). [file 12934_2014_148_MOESM3_ESM.zip › Additional File 3A/llmg_0148_real_dat.png]

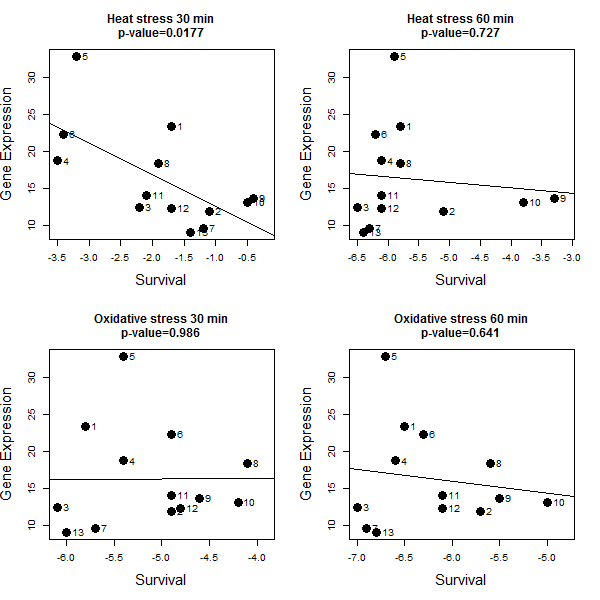

Supplement: Additional file 3: — Plots of gene expression and robustness levels. Expression levels of all genes plotted against survival after 30 and 60 minutes heat and oxidative stress (A: genes llmg_0001 to llmg_1229, B: genes llmg_1230 to llmg_2563). Survival is expressed as the difference of log CFU/ml after stress and before stress. Numbers indicate fermentations as presented in Table 1. P-values above the plots indicate significance of correlation (assessed by a linear model). [file 12934_2014_148_MOESM3_ESM.zip › Additional File 3A/llmg_0149_real_dat.png]

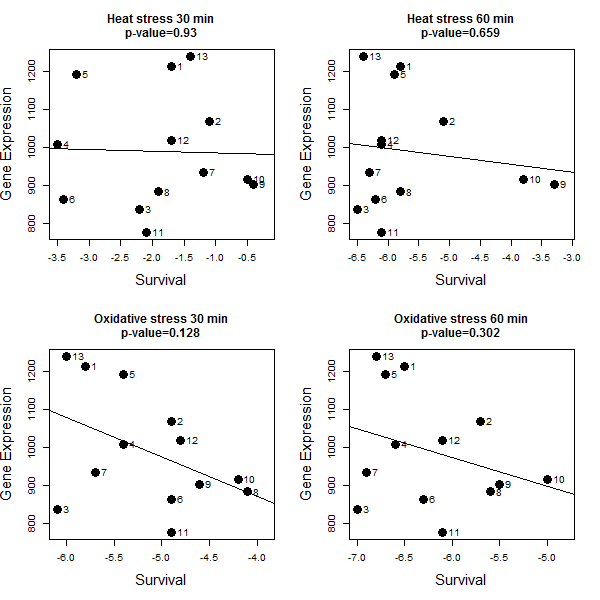

Supplement: Additional file 3: — Plots of gene expression and robustness levels. Expression levels of all genes plotted against survival after 30 and 60 minutes heat and oxidative stress (A: genes llmg_0001 to llmg_1229, B: genes llmg_1230 to llmg_2563). Survival is expressed as the difference of log CFU/ml after stress and before stress. Numbers indicate fermentations as presented in Table 1. P-values above the plots indicate significance of correlation (assessed by a linear model). [file 12934_2014_148_MOESM3_ESM.zip › Additional File 3A/llmg_0150_real_dat.png]

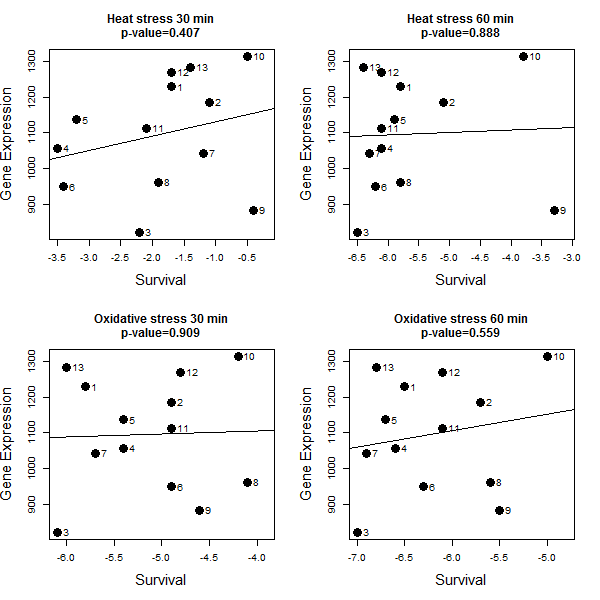

Supplement: Additional file 3: — Plots of gene expression and robustness levels. Expression levels of all genes plotted against survival after 30 and 60 minutes heat and oxidative stress (A: genes llmg_0001 to llmg_1229, B: genes llmg_1230 to llmg_2563). Survival is expressed as the difference of log CFU/ml after stress and before stress. Numbers indicate fermentations as presented in Table 1. P-values above the plots indicate significance of correlation (assessed by a linear model). [file 12934_2014_148_MOESM3_ESM.zip › Additional File 3A/llmg_0151_real_dat.png]

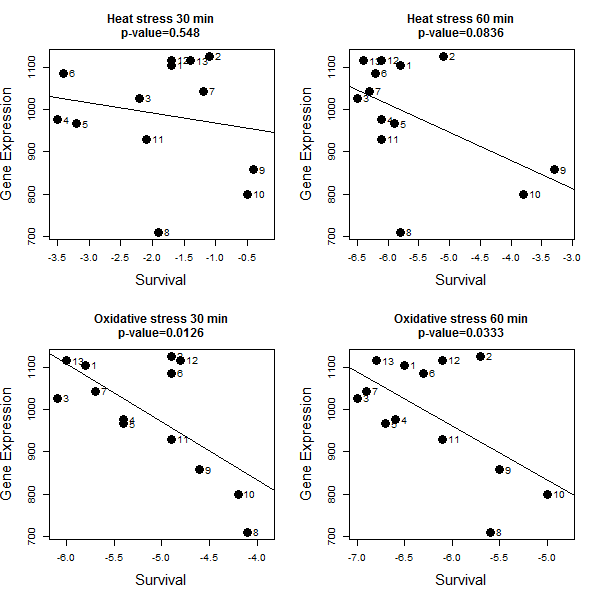

Supplement: Additional file 3: — Plots of gene expression and robustness levels. Expression levels of all genes plotted against survival after 30 and 60 minutes heat and oxidative stress (A: genes llmg_0001 to llmg_1229, B: genes llmg_1230 to llmg_2563). Survival is expressed as the difference of log CFU/ml after stress and before stress. Numbers indicate fermentations as presented in Table 1. P-values above the plots indicate significance of correlation (assessed by a linear model). [file 12934_2014_148_MOESM3_ESM.zip › Additional File 3A/llmg_0152_real_dat.png]

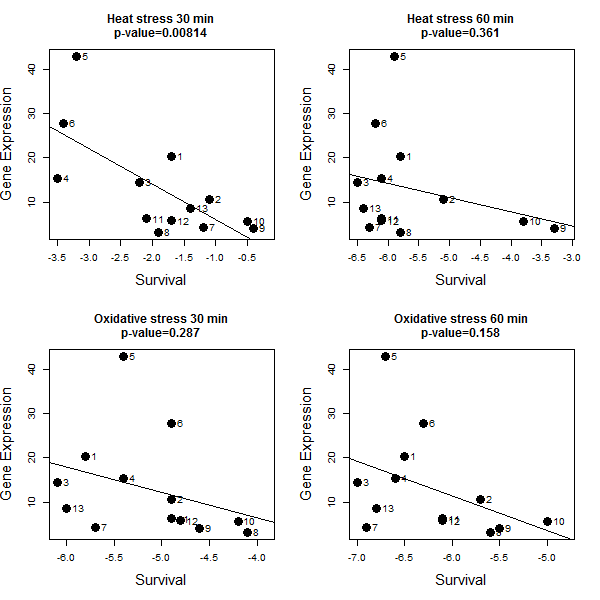

Supplement: Additional file 3: — Plots of gene expression and robustness levels. Expression levels of all genes plotted against survival after 30 and 60 minutes heat and oxidative stress (A: genes llmg_0001 to llmg_1229, B: genes llmg_1230 to llmg_2563). Survival is expressed as the difference of log CFU/ml after stress and before stress. Numbers indicate fermentations as presented in Table 1. P-values above the plots indicate significance of correlation (assessed by a linear model). [file 12934_2014_148_MOESM3_ESM.zip › Additional File 3A/llmg_0153_real_dat.png]

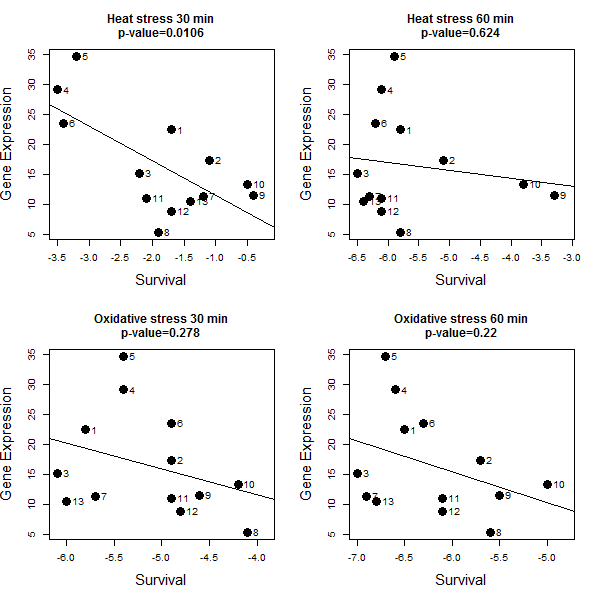

Supplement: Additional file 3: — Plots of gene expression and robustness levels. Expression levels of all genes plotted against survival after 30 and 60 minutes heat and oxidative stress (A: genes llmg_0001 to llmg_1229, B: genes llmg_1230 to llmg_2563). Survival is expressed as the difference of log CFU/ml after stress and before stress. Numbers indicate fermentations as presented in Table 1. P-values above the plots indicate significance of correlation (assessed by a linear model). [file 12934_2014_148_MOESM3_ESM.zip › Additional File 3A/llmg_0154_real_dat.png]

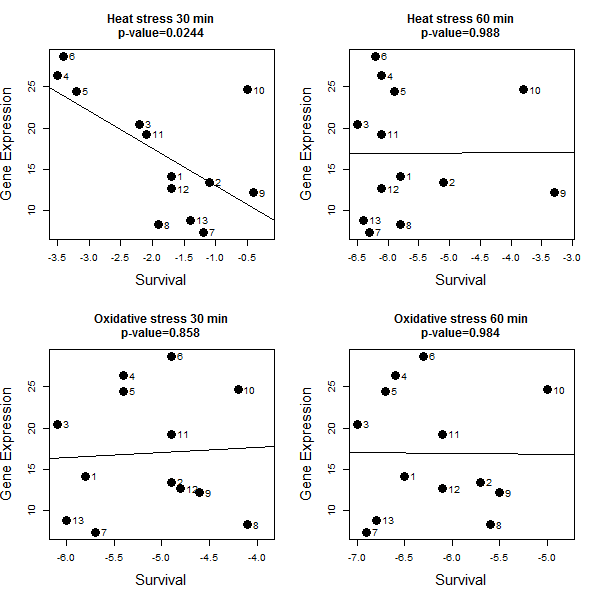

Supplement: Additional file 3: — Plots of gene expression and robustness levels. Expression levels of all genes plotted against survival after 30 and 60 minutes heat and oxidative stress (A: genes llmg_0001 to llmg_1229, B: genes llmg_1230 to llmg_2563). Survival is expressed as the difference of log CFU/ml after stress and before stress. Numbers indicate fermentations as presented in Table 1. P-values above the plots indicate significance of correlation (assessed by a linear model). [file 12934_2014_148_MOESM3_ESM.zip › Additional File 3A/llmg_0155_real_dat.png]

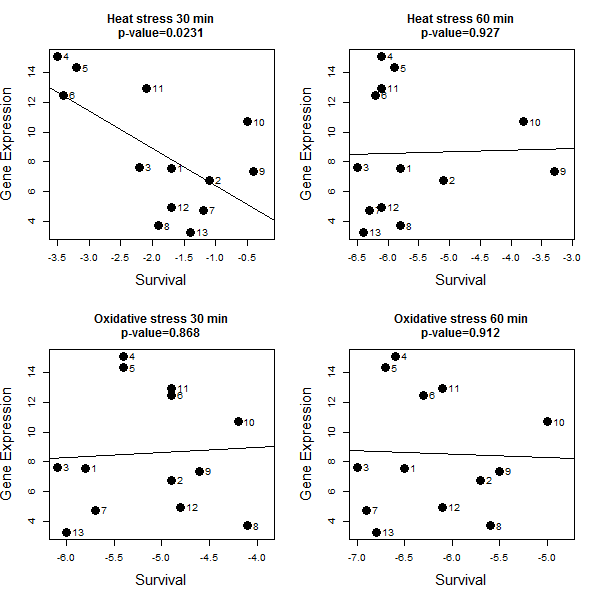

Supplement: Additional file 3: — Plots of gene expression and robustness levels. Expression levels of all genes plotted against survival after 30 and 60 minutes heat and oxidative stress (A: genes llmg_0001 to llmg_1229, B: genes llmg_1230 to llmg_2563). Survival is expressed as the difference of log CFU/ml after stress and before stress. Numbers indicate fermentations as presented in Table 1. P-values above the plots indicate significance of correlation (assessed by a linear model). [file 12934_2014_148_MOESM3_ESM.zip › Additional File 3A/llmg_0156_real_dat.png]

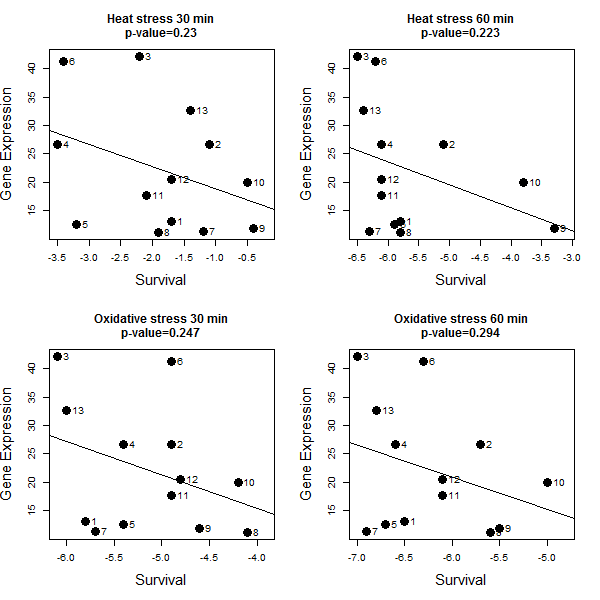

Supplement: Additional file 3: — Plots of gene expression and robustness levels. Expression levels of all genes plotted against survival after 30 and 60 minutes heat and oxidative stress (A: genes llmg_0001 to llmg_1229, B: genes llmg_1230 to llmg_2563). Survival is expressed as the difference of log CFU/ml after stress and before stress. Numbers indicate fermentations as presented in Table 1. P-values above the plots indicate significance of correlation (assessed by a linear model). [file 12934_2014_148_MOESM3_ESM.zip › Additional File 3A/llmg_0157_real_dat.png]

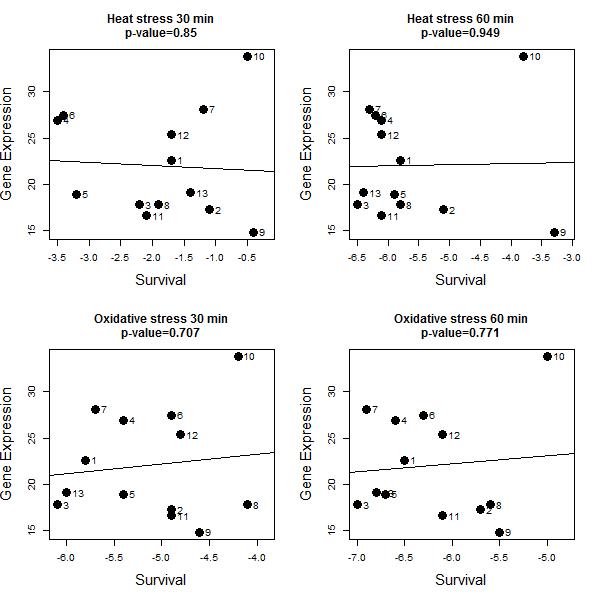

Supplement: Additional file 3: — Plots of gene expression and robustness levels. Expression levels of all genes plotted against survival after 30 and 60 minutes heat and oxidative stress (A: genes llmg_0001 to llmg_1229, B: genes llmg_1230 to llmg_2563). Survival is expressed as the difference of log CFU/ml after stress and before stress. Numbers indicate fermentations as presented in Table 1. P-values above the plots indicate significance of correlation (assessed by a linear model). [file 12934_2014_148_MOESM3_ESM.zip › Additional File 3A/llmg_0158_real_dat.png]

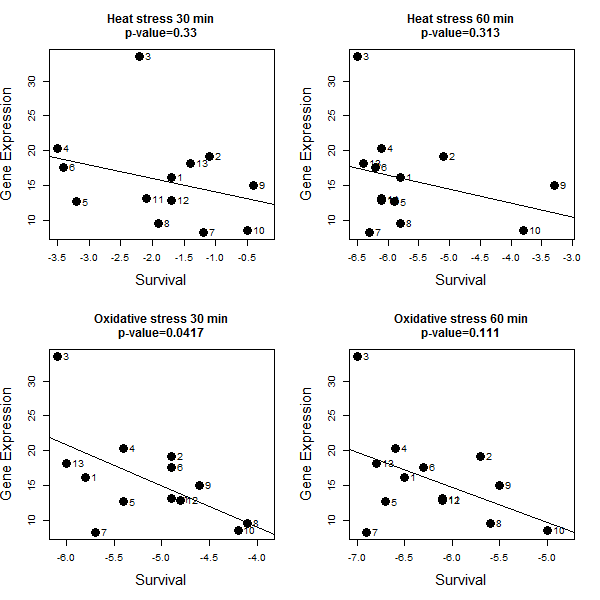

Supplement: Additional file 3: — Plots of gene expression and robustness levels. Expression levels of all genes plotted against survival after 30 and 60 minutes heat and oxidative stress (A: genes llmg_0001 to llmg_1229, B: genes llmg_1230 to llmg_2563). Survival is expressed as the difference of log CFU/ml after stress and before stress. Numbers indicate fermentations as presented in Table 1. P-values above the plots indicate significance of correlation (assessed by a linear model). [file 12934_2014_148_MOESM3_ESM.zip › Additional File 3A/llmg_0159_real_dat.png]

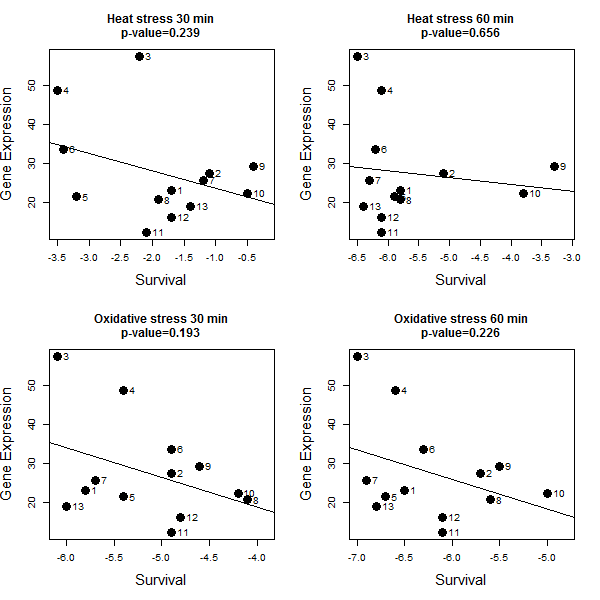

Supplement: Additional file 3: — Plots of gene expression and robustness levels. Expression levels of all genes plotted against survival after 30 and 60 minutes heat and oxidative stress (A: genes llmg_0001 to llmg_1229, B: genes llmg_1230 to llmg_2563). Survival is expressed as the difference of log CFU/ml after stress and before stress. Numbers indicate fermentations as presented in Table 1. P-values above the plots indicate significance of correlation (assessed by a linear model). [file 12934_2014_148_MOESM3_ESM.zip › Additional File 3A/llmg_0160_real_dat.png]

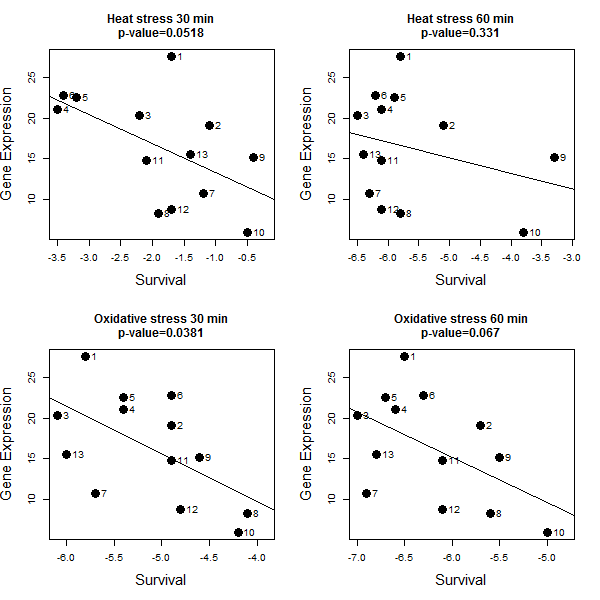

Supplement: Additional file 3: — Plots of gene expression and robustness levels. Expression levels of all genes plotted against survival after 30 and 60 minutes heat and oxidative stress (A: genes llmg_0001 to llmg_1229, B: genes llmg_1230 to llmg_2563). Survival is expressed as the difference of log CFU/ml after stress and before stress. Numbers indicate fermentations as presented in Table 1. P-values above the plots indicate significance of correlation (assessed by a linear model). [file 12934_2014_148_MOESM3_ESM.zip › Additional File 3A/llmg_0161_real_dat.png]

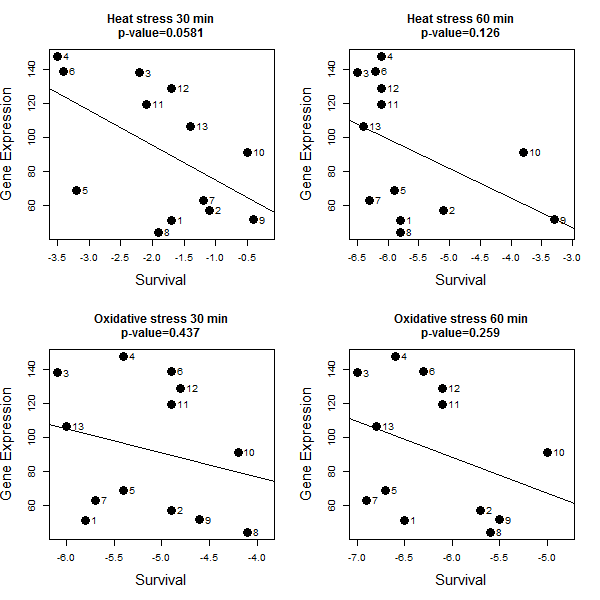

Supplement: Additional file 3: — Plots of gene expression and robustness levels. Expression levels of all genes plotted against survival after 30 and 60 minutes heat and oxidative stress (A: genes llmg_0001 to llmg_1229, B: genes llmg_1230 to llmg_2563). Survival is expressed as the difference of log CFU/ml after stress and before stress. Numbers indicate fermentations as presented in Table 1. P-values above the plots indicate significance of correlation (assessed by a linear model). [file 12934_2014_148_MOESM3_ESM.zip › Additional File 3A/llmg_0162_real_dat.png]

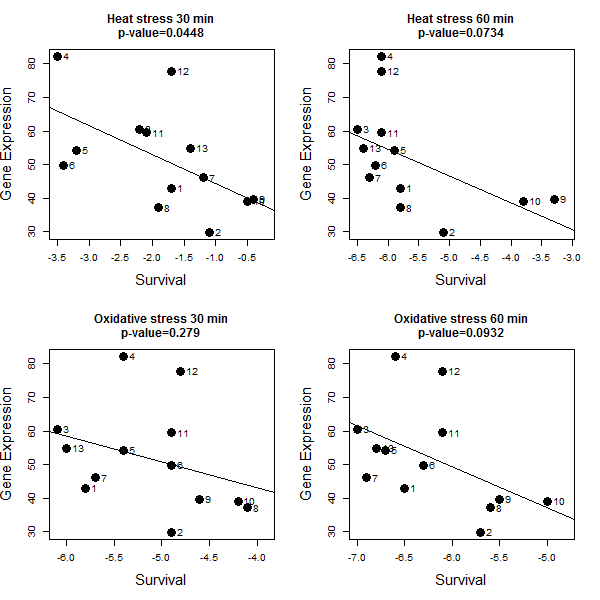

Supplement: Additional file 3: — Plots of gene expression and robustness levels. Expression levels of all genes plotted against survival after 30 and 60 minutes heat and oxidative stress (A: genes llmg_0001 to llmg_1229, B: genes llmg_1230 to llmg_2563). Survival is expressed as the difference of log CFU/ml after stress and before stress. Numbers indicate fermentations as presented in Table 1. P-values above the plots indicate significance of correlation (assessed by a linear model). [file 12934_2014_148_MOESM3_ESM.zip › Additional File 3A/llmg_0163_real_dat.png]

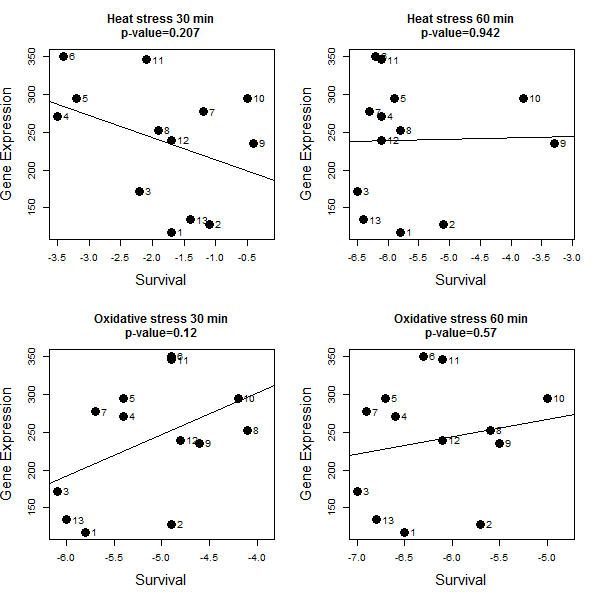

Supplement: Additional file 3: — Plots of gene expression and robustness levels. Expression levels of all genes plotted against survival after 30 and 60 minutes heat and oxidative stress (A: genes llmg_0001 to llmg_1229, B: genes llmg_1230 to llmg_2563). Survival is expressed as the difference of log CFU/ml after stress and before stress. Numbers indicate fermentations as presented in Table 1. P-values above the plots indicate significance of correlation (assessed by a linear model). [file 12934_2014_148_MOESM3_ESM.zip › Additional File 3A/llmg_0164_real_dat.png]

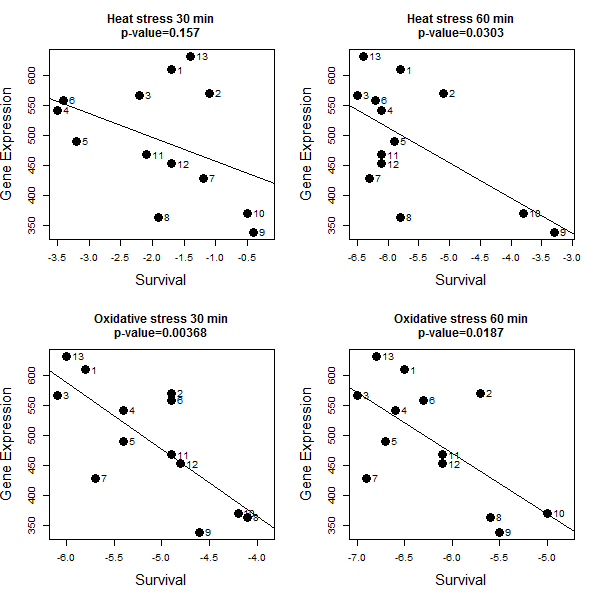

Supplement: Additional file 3: — Plots of gene expression and robustness levels. Expression levels of all genes plotted against survival after 30 and 60 minutes heat and oxidative stress (A: genes llmg_0001 to llmg_1229, B: genes llmg_1230 to llmg_2563). Survival is expressed as the difference of log CFU/ml after stress and before stress. Numbers indicate fermentations as presented in Table 1. P-values above the plots indicate significance of correlation (assessed by a linear model). [file 12934_2014_148_MOESM3_ESM.zip › Additional File 3A/llmg_0165_real_dat.png]

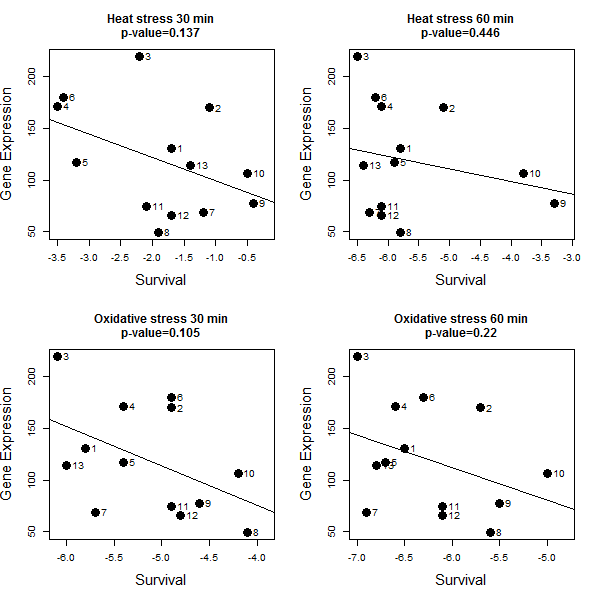

Supplement: Additional file 3: — Plots of gene expression and robustness levels. Expression levels of all genes plotted against survival after 30 and 60 minutes heat and oxidative stress (A: genes llmg_0001 to llmg_1229, B: genes llmg_1230 to llmg_2563). Survival is expressed as the difference of log CFU/ml after stress and before stress. Numbers indicate fermentations as presented in Table 1. P-values above the plots indicate significance of correlation (assessed by a linear model). [file 12934_2014_148_MOESM3_ESM.zip › Additional File 3A/llmg_0167_real_dat.png]

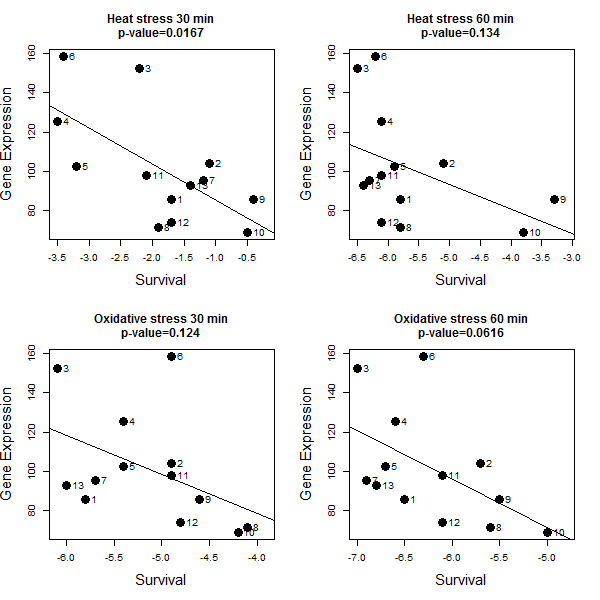

Supplement: Additional file 3: — Plots of gene expression and robustness levels. Expression levels of all genes plotted against survival after 30 and 60 minutes heat and oxidative stress (A: genes llmg_0001 to llmg_1229, B: genes llmg_1230 to llmg_2563). Survival is expressed as the difference of log CFU/ml after stress and before stress. Numbers indicate fermentations as presented in Table 1. P-values above the plots indicate significance of correlation (assessed by a linear model). [file 12934_2014_148_MOESM3_ESM.zip › Additional File 3A/llmg_0168_real_dat.png]

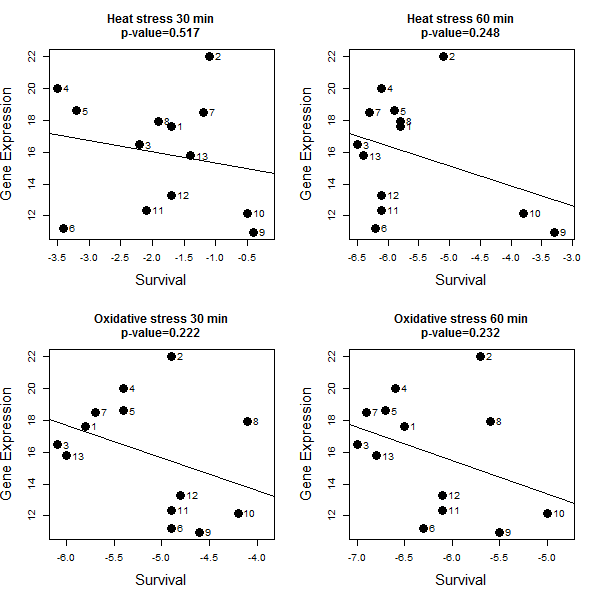

Supplement: Additional file 3: — Plots of gene expression and robustness levels. Expression levels of all genes plotted against survival after 30 and 60 minutes heat and oxidative stress (A: genes llmg_0001 to llmg_1229, B: genes llmg_1230 to llmg_2563). Survival is expressed as the difference of log CFU/ml after stress and before stress. Numbers indicate fermentations as presented in Table 1. P-values above the plots indicate significance of correlation (assessed by a linear model). [file 12934_2014_148_MOESM3_ESM.zip › Additional File 3A/llmg_0169_real_dat.png]

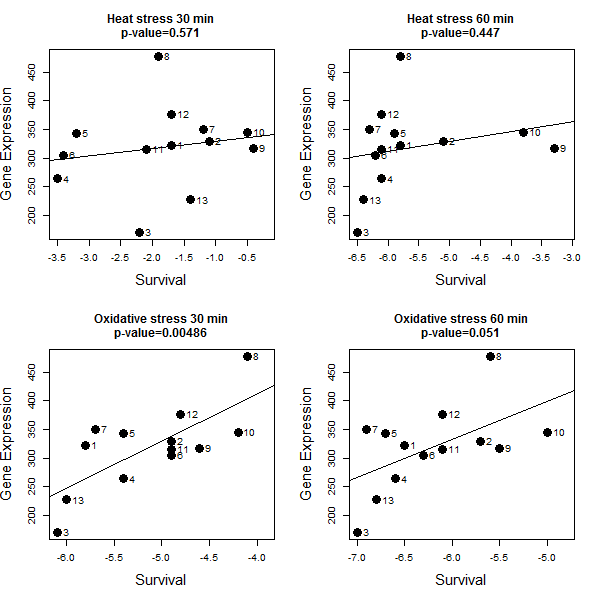

Supplement: Additional file 3: — Plots of gene expression and robustness levels. Expression levels of all genes plotted against survival after 30 and 60 minutes heat and oxidative stress (A: genes llmg_0001 to llmg_1229, B: genes llmg_1230 to llmg_2563). Survival is expressed as the difference of log CFU/ml after stress and before stress. Numbers indicate fermentations as presented in Table 1. P-values above the plots indicate significance of correlation (assessed by a linear model). [file 12934_2014_148_MOESM3_ESM.zip › Additional File 3A/llmg_0170_real_dat.png]

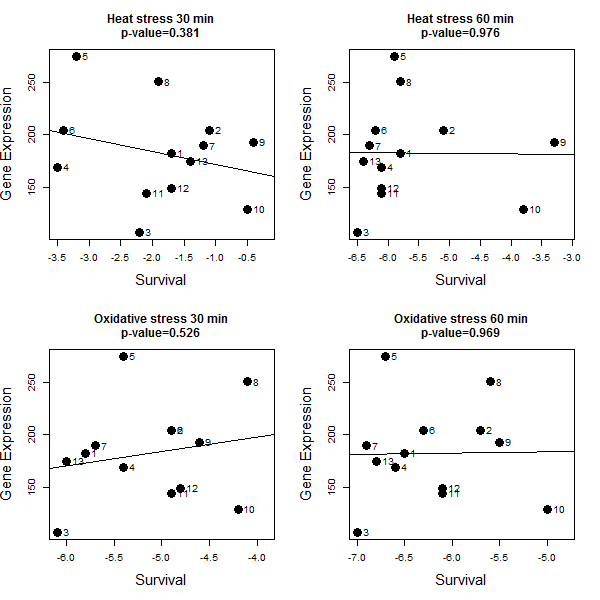

Supplement: Additional file 3: — Plots of gene expression and robustness levels. Expression levels of all genes plotted against survival after 30 and 60 minutes heat and oxidative stress (A: genes llmg_0001 to llmg_1229, B: genes llmg_1230 to llmg_2563). Survival is expressed as the difference of log CFU/ml after stress and before stress. Numbers indicate fermentations as presented in Table 1. P-values above the plots indicate significance of correlation (assessed by a linear model). [file 12934_2014_148_MOESM3_ESM.zip › Additional File 3A/llmg_0171_real_dat.png]

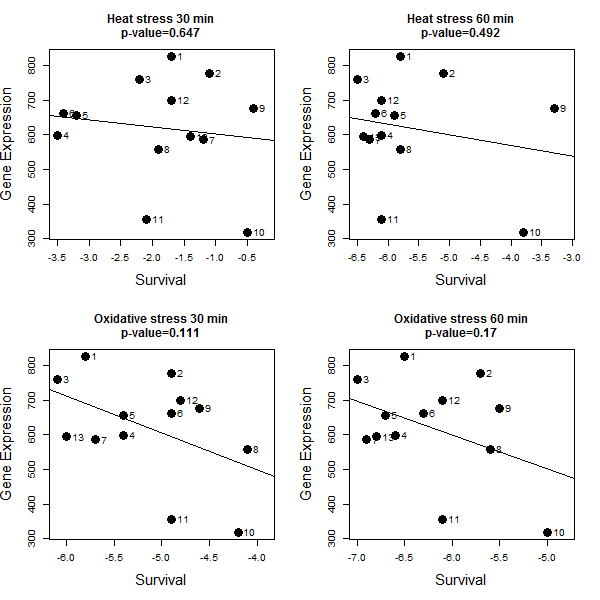

Supplement: Additional file 3: — Plots of gene expression and robustness levels. Expression levels of all genes plotted against survival after 30 and 60 minutes heat and oxidative stress (A: genes llmg_0001 to llmg_1229, B: genes llmg_1230 to llmg_2563). Survival is expressed as the difference of log CFU/ml after stress and before stress. Numbers indicate fermentations as presented in Table 1. P-values above the plots indicate significance of correlation (assessed by a linear model). [file 12934_2014_148_MOESM3_ESM.zip › Additional File 3A/llmg_0172_real_dat.png]

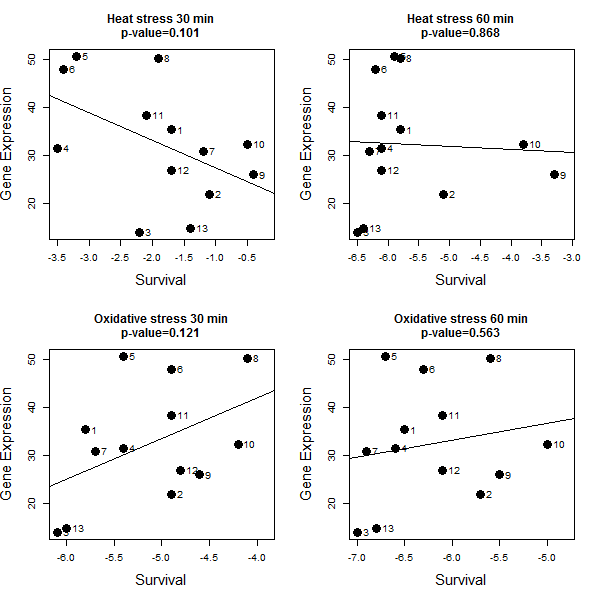

Supplement: Additional file 3: — Plots of gene expression and robustness levels. Expression levels of all genes plotted against survival after 30 and 60 minutes heat and oxidative stress (A: genes llmg_0001 to llmg_1229, B: genes llmg_1230 to llmg_2563). Survival is expressed as the difference of log CFU/ml after stress and before stress. Numbers indicate fermentations as presented in Table 1. P-values above the plots indicate significance of correlation (assessed by a linear model). [file 12934_2014_148_MOESM3_ESM.zip › Additional File 3A/llmg_0173_real_dat.png]

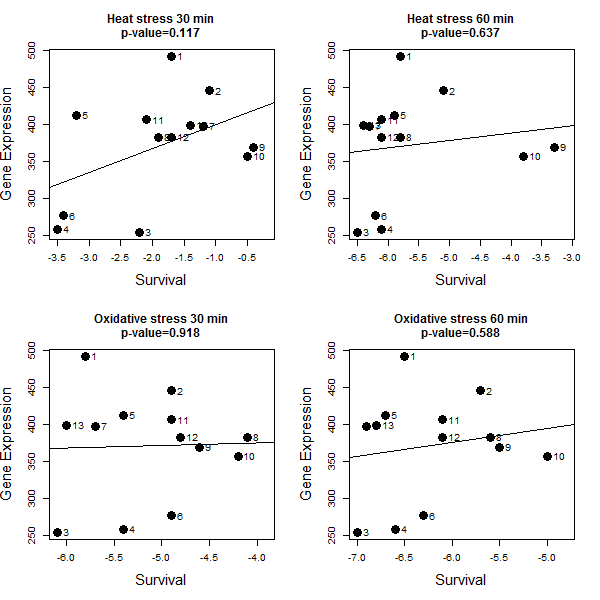

Supplement: Additional file 3: — Plots of gene expression and robustness levels. Expression levels of all genes plotted against survival after 30 and 60 minutes heat and oxidative stress (A: genes llmg_0001 to llmg_1229, B: genes llmg_1230 to llmg_2563). Survival is expressed as the difference of log CFU/ml after stress and before stress. Numbers indicate fermentations as presented in Table 1. P-values above the plots indicate significance of correlation (assessed by a linear model). [file 12934_2014_148_MOESM3_ESM.zip › Additional File 3A/llmg_0174_real_dat.png]

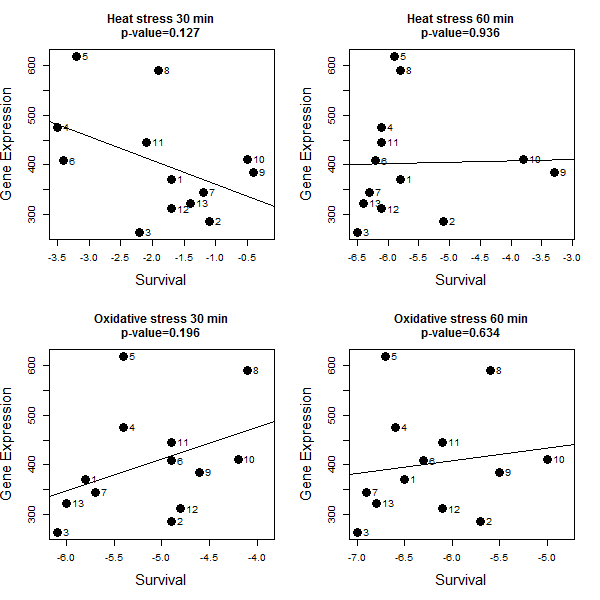

Supplement: Additional file 3: — Plots of gene expression and robustness levels. Expression levels of all genes plotted against survival after 30 and 60 minutes heat and oxidative stress (A: genes llmg_0001 to llmg_1229, B: genes llmg_1230 to llmg_2563). Survival is expressed as the difference of log CFU/ml after stress and before stress. Numbers indicate fermentations as presented in Table 1. P-values above the plots indicate significance of correlation (assessed by a linear model). [file 12934_2014_148_MOESM3_ESM.zip › Additional File 3A/llmg_0175_real_dat.png]

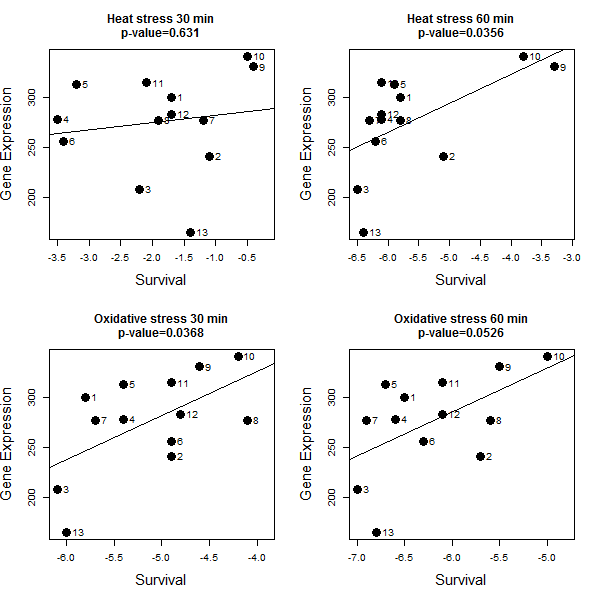

Supplement: Additional file 3: — Plots of gene expression and robustness levels. Expression levels of all genes plotted against survival after 30 and 60 minutes heat and oxidative stress (A: genes llmg_0001 to llmg_1229, B: genes llmg_1230 to llmg_2563). Survival is expressed as the difference of log CFU/ml after stress and before stress. Numbers indicate fermentations as presented in Table 1. P-values above the plots indicate significance of correlation (assessed by a linear model). [file 12934_2014_148_MOESM3_ESM.zip › Additional File 3A/llmg_0176_real_dat.png]

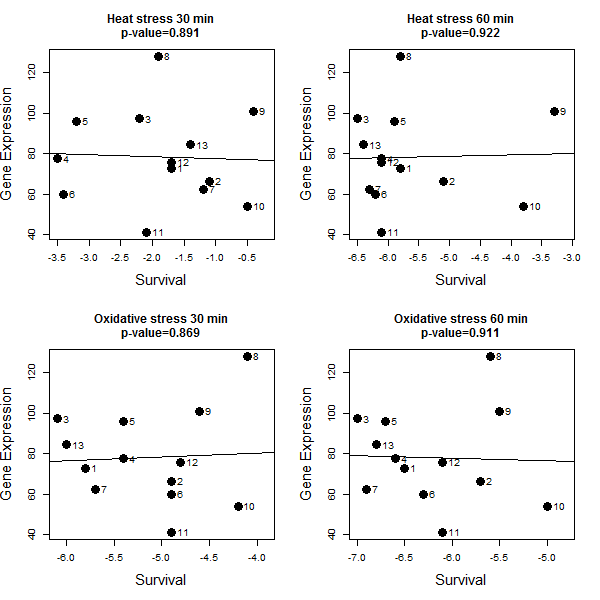

Supplement: Additional file 3: — Plots of gene expression and robustness levels. Expression levels of all genes plotted against survival after 30 and 60 minutes heat and oxidative stress (A: genes llmg_0001 to llmg_1229, B: genes llmg_1230 to llmg_2563). Survival is expressed as the difference of log CFU/ml after stress and before stress. Numbers indicate fermentations as presented in Table 1. P-values above the plots indicate significance of correlation (assessed by a linear model). [file 12934_2014_148_MOESM3_ESM.zip › Additional File 3A/llmg_0177_real_dat.png]

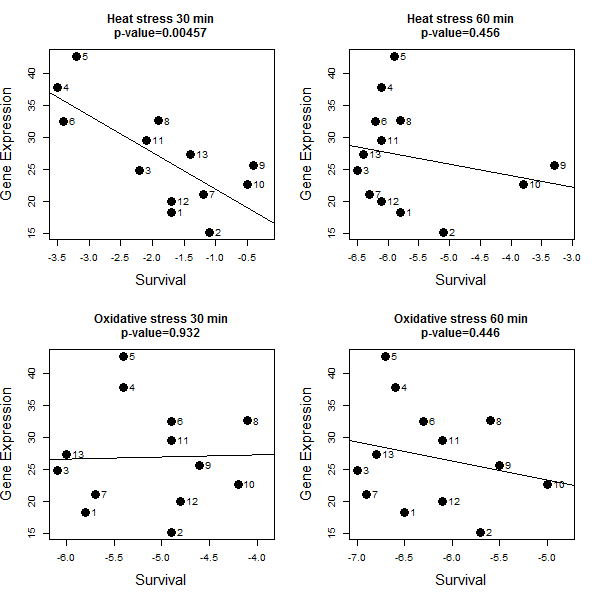

Supplement: Additional file 3: — Plots of gene expression and robustness levels. Expression levels of all genes plotted against survival after 30 and 60 minutes heat and oxidative stress (A: genes llmg_0001 to llmg_1229, B: genes llmg_1230 to llmg_2563). Survival is expressed as the difference of log CFU/ml after stress and before stress. Numbers indicate fermentations as presented in Table 1. P-values above the plots indicate significance of correlation (assessed by a linear model). [file 12934_2014_148_MOESM3_ESM.zip › Additional File 3A/llmg_0178_real_dat.png]

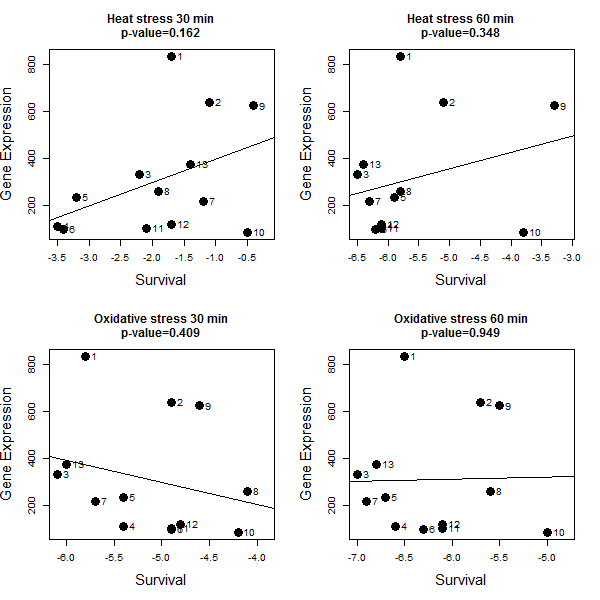

Supplement: Additional file 3: — Plots of gene expression and robustness levels. Expression levels of all genes plotted against survival after 30 and 60 minutes heat and oxidative stress (A: genes llmg_0001 to llmg_1229, B: genes llmg_1230 to llmg_2563). Survival is expressed as the difference of log CFU/ml after stress and before stress. Numbers indicate fermentations as presented in Table 1. P-values above the plots indicate significance of correlation (assessed by a linear model). [file 12934_2014_148_MOESM3_ESM.zip › Additional File 3A/llmg_0180_real_dat.png]

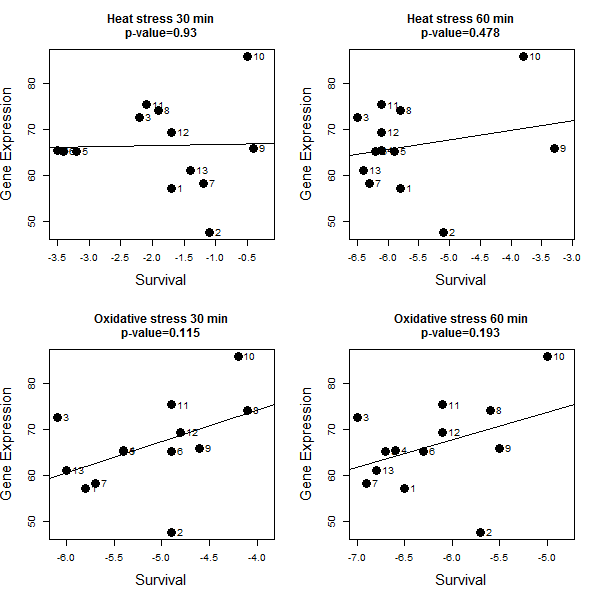

Supplement: Additional file 3: — Plots of gene expression and robustness levels. Expression levels of all genes plotted against survival after 30 and 60 minutes heat and oxidative stress (A: genes llmg_0001 to llmg_1229, B: genes llmg_1230 to llmg_2563). Survival is expressed as the difference of log CFU/ml after stress and before stress. Numbers indicate fermentations as presented in Table 1. P-values above the plots indicate significance of correlation (assessed by a linear model). [file 12934_2014_148_MOESM3_ESM.zip › Additional File 3A/llmg_0181_real_dat.png]

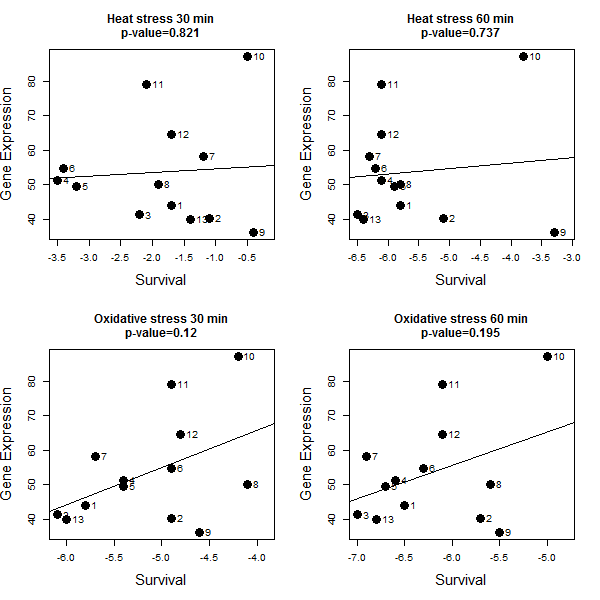

Supplement: Additional file 3: — Plots of gene expression and robustness levels. Expression levels of all genes plotted against survival after 30 and 60 minutes heat and oxidative stress (A: genes llmg_0001 to llmg_1229, B: genes llmg_1230 to llmg_2563). Survival is expressed as the difference of log CFU/ml after stress and before stress. Numbers indicate fermentations as presented in Table 1. P-values above the plots indicate significance of correlation (assessed by a linear model). [file 12934_2014_148_MOESM3_ESM.zip › Additional File 3A/llmg_0182_real_dat.png]

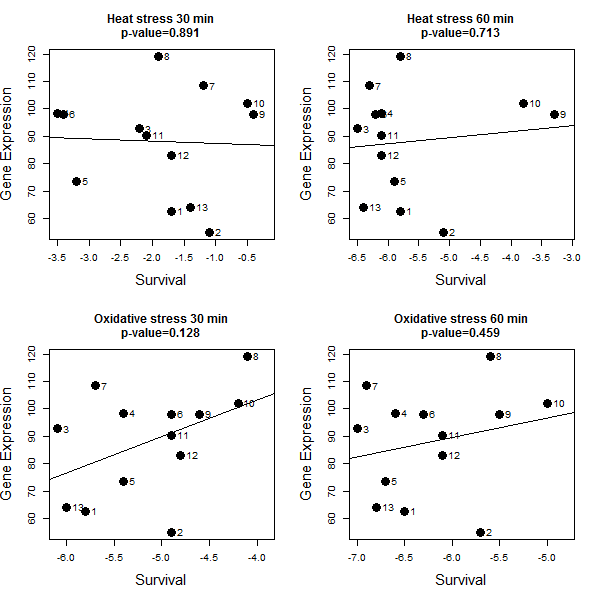

Supplement: Additional file 3: — Plots of gene expression and robustness levels. Expression levels of all genes plotted against survival after 30 and 60 minutes heat and oxidative stress (A: genes llmg_0001 to llmg_1229, B: genes llmg_1230 to llmg_2563). Survival is expressed as the difference of log CFU/ml after stress and before stress. Numbers indicate fermentations as presented in Table 1. P-values above the plots indicate significance of correlation (assessed by a linear model). [file 12934_2014_148_MOESM3_ESM.zip › Additional File 3A/llmg_0183_real_dat.png]

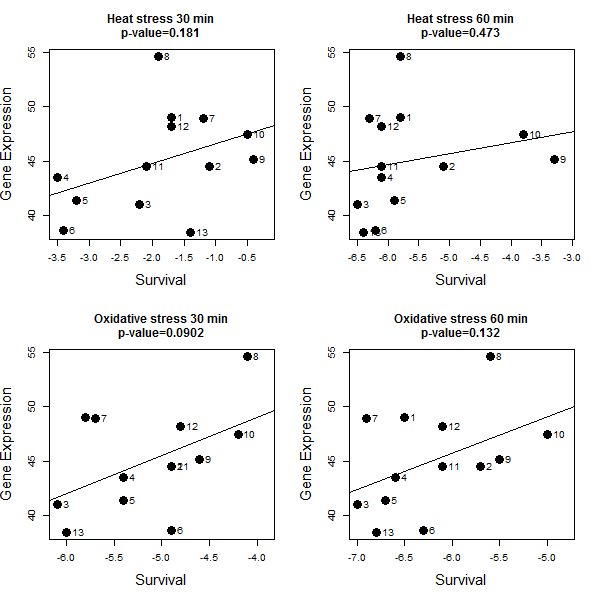

Supplement: Additional file 3: — Plots of gene expression and robustness levels. Expression levels of all genes plotted against survival after 30 and 60 minutes heat and oxidative stress (A: genes llmg_0001 to llmg_1229, B: genes llmg_1230 to llmg_2563). Survival is expressed as the difference of log CFU/ml after stress and before stress. Numbers indicate fermentations as presented in Table 1. P-values above the plots indicate significance of correlation (assessed by a linear model). [file 12934_2014_148_MOESM3_ESM.zip › Additional File 3A/llmg_0184_real_dat.png]

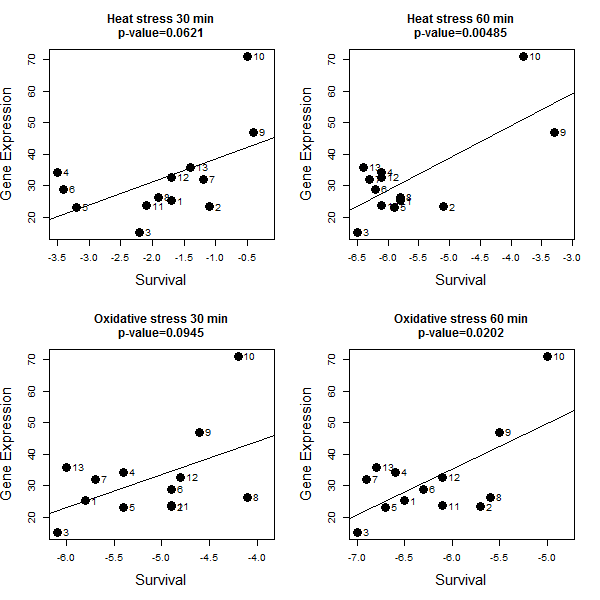

Supplement: Additional file 3: — Plots of gene expression and robustness levels. Expression levels of all genes plotted against survival after 30 and 60 minutes heat and oxidative stress (A: genes llmg_0001 to llmg_1229, B: genes llmg_1230 to llmg_2563). Survival is expressed as the difference of log CFU/ml after stress and before stress. Numbers indicate fermentations as presented in Table 1. P-values above the plots indicate significance of correlation (assessed by a linear model). [file 12934_2014_148_MOESM3_ESM.zip › Additional File 3A/llmg_0185_real_dat.png]

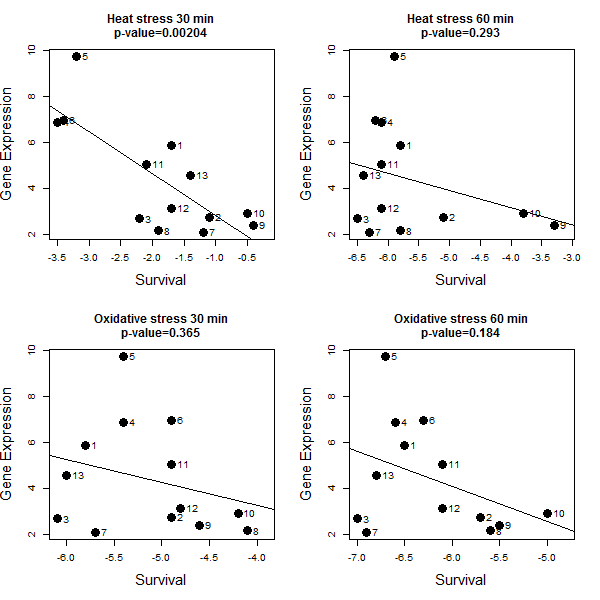

Supplement: Additional file 3: — Plots of gene expression and robustness levels. Expression levels of all genes plotted against survival after 30 and 60 minutes heat and oxidative stress (A: genes llmg_0001 to llmg_1229, B: genes llmg_1230 to llmg_2563). Survival is expressed as the difference of log CFU/ml after stress and before stress. Numbers indicate fermentations as presented in Table 1. P-values above the plots indicate significance of correlation (assessed by a linear model). [file 12934_2014_148_MOESM3_ESM.zip › Additional File 3A/llmg_0186_real_dat.png]

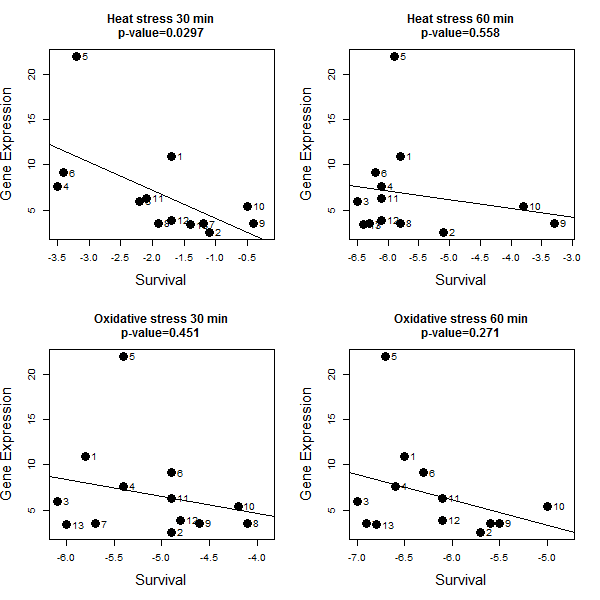

Supplement: Additional file 3: — Plots of gene expression and robustness levels. Expression levels of all genes plotted against survival after 30 and 60 minutes heat and oxidative stress (A: genes llmg_0001 to llmg_1229, B: genes llmg_1230 to llmg_2563). Survival is expressed as the difference of log CFU/ml after stress and before stress. Numbers indicate fermentations as presented in Table 1. P-values above the plots indicate significance of correlation (assessed by a linear model). [file 12934_2014_148_MOESM3_ESM.zip › Additional File 3A/llmg_0187_real_dat.png]

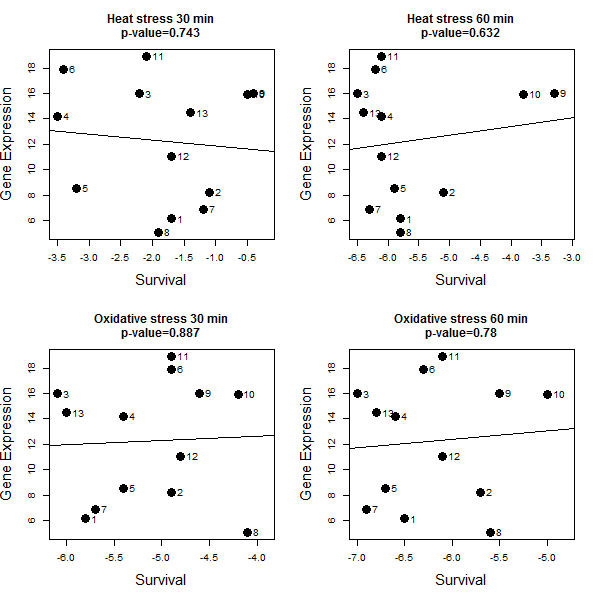

Supplement: Additional file 3: — Plots of gene expression and robustness levels. Expression levels of all genes plotted against survival after 30 and 60 minutes heat and oxidative stress (A: genes llmg_0001 to llmg_1229, B: genes llmg_1230 to llmg_2563). Survival is expressed as the difference of log CFU/ml after stress and before stress. Numbers indicate fermentations as presented in Table 1. P-values above the plots indicate significance of correlation (assessed by a linear model). [file 12934_2014_148_MOESM3_ESM.zip › Additional File 3A/llmg_0188_real_dat.png]

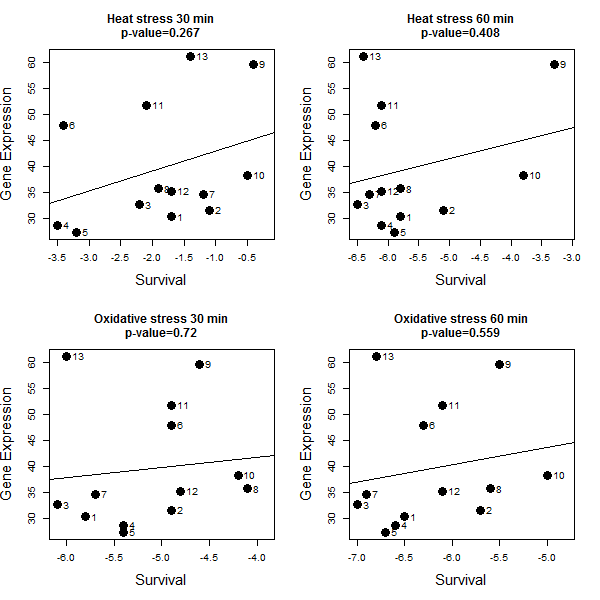

Supplement: Additional file 3: — Plots of gene expression and robustness levels. Expression levels of all genes plotted against survival after 30 and 60 minutes heat and oxidative stress (A: genes llmg_0001 to llmg_1229, B: genes llmg_1230 to llmg_2563). Survival is expressed as the difference of log CFU/ml after stress and before stress. Numbers indicate fermentations as presented in Table 1. P-values above the plots indicate significance of correlation (assessed by a linear model). [file 12934_2014_148_MOESM3_ESM.zip › Additional File 3A/llmg_0189_real_dat.png]

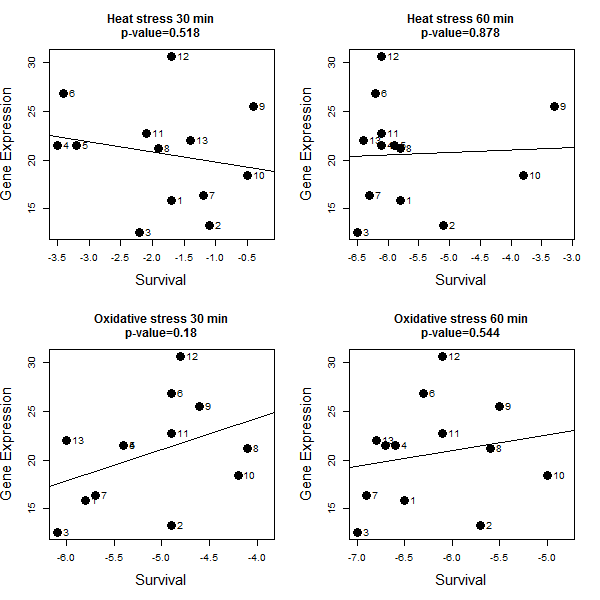

Supplement: Additional file 3: — Plots of gene expression and robustness levels. Expression levels of all genes plotted against survival after 30 and 60 minutes heat and oxidative stress (A: genes llmg_0001 to llmg_1229, B: genes llmg_1230 to llmg_2563). Survival is expressed as the difference of log CFU/ml after stress and before stress. Numbers indicate fermentations as presented in Table 1. P-values above the plots indicate significance of correlation (assessed by a linear model). [file 12934_2014_148_MOESM3_ESM.zip › Additional File 3A/llmg_0190_real_dat.png]

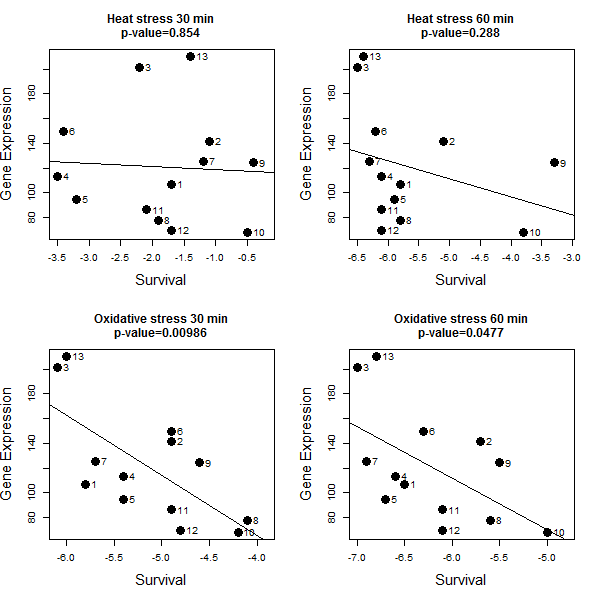

Supplement: Additional file 3: — Plots of gene expression and robustness levels. Expression levels of all genes plotted against survival after 30 and 60 minutes heat and oxidative stress (A: genes llmg_0001 to llmg_1229, B: genes llmg_1230 to llmg_2563). Survival is expressed as the difference of log CFU/ml after stress and before stress. Numbers indicate fermentations as presented in Table 1. P-values above the plots indicate significance of correlation (assessed by a linear model). [file 12934_2014_148_MOESM3_ESM.zip › Additional File 3A/llmg_0191_real_dat.png]

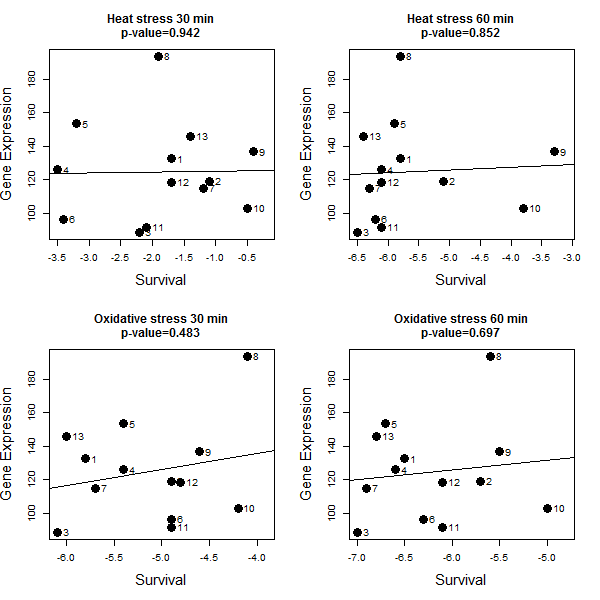

Supplement: Additional file 3: — Plots of gene expression and robustness levels. Expression levels of all genes plotted against survival after 30 and 60 minutes heat and oxidative stress (A: genes llmg_0001 to llmg_1229, B: genes llmg_1230 to llmg_2563). Survival is expressed as the difference of log CFU/ml after stress and before stress. Numbers indicate fermentations as presented in Table 1. P-values above the plots indicate significance of correlation (assessed by a linear model). [file 12934_2014_148_MOESM3_ESM.zip › Additional File 3A/llmg_0192_real_dat.png]

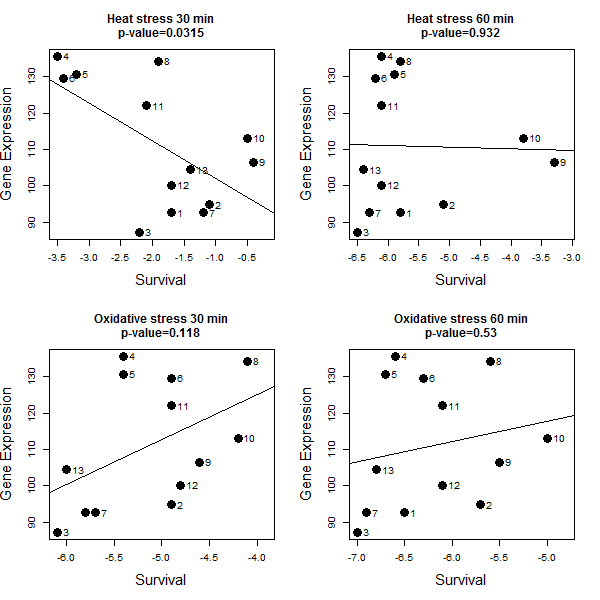

Supplement: Additional file 3: — Plots of gene expression and robustness levels. Expression levels of all genes plotted against survival after 30 and 60 minutes heat and oxidative stress (A: genes llmg_0001 to llmg_1229, B: genes llmg_1230 to llmg_2563). Survival is expressed as the difference of log CFU/ml after stress and before stress. Numbers indicate fermentations as presented in Table 1. P-values above the plots indicate significance of correlation (assessed by a linear model). [file 12934_2014_148_MOESM3_ESM.zip › Additional File 3A/llmg_0193_real_dat.png]

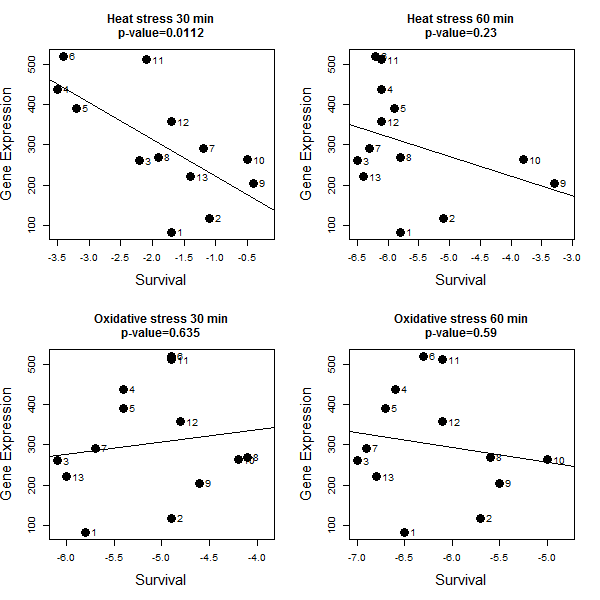

Supplement: Additional file 3: — Plots of gene expression and robustness levels. Expression levels of all genes plotted against survival after 30 and 60 minutes heat and oxidative stress (A: genes llmg_0001 to llmg_1229, B: genes llmg_1230 to llmg_2563). Survival is expressed as the difference of log CFU/ml after stress and before stress. Numbers indicate fermentations as presented in Table 1. P-values above the plots indicate significance of correlation (assessed by a linear model). [file 12934_2014_148_MOESM3_ESM.zip › Additional File 3A/llmg_0194_real_dat.png]

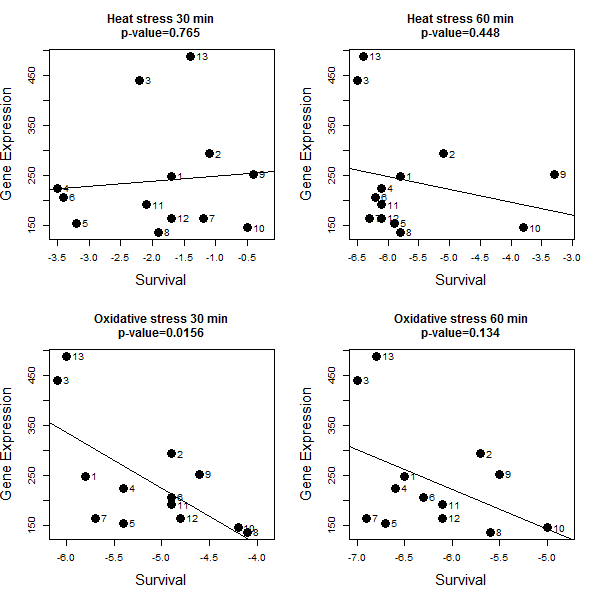

Supplement: Additional file 3: — Plots of gene expression and robustness levels. Expression levels of all genes plotted against survival after 30 and 60 minutes heat and oxidative stress (A: genes llmg_0001 to llmg_1229, B: genes llmg_1230 to llmg_2563). Survival is expressed as the difference of log CFU/ml after stress and before stress. Numbers indicate fermentations as presented in Table 1. P-values above the plots indicate significance of correlation (assessed by a linear model). [file 12934_2014_148_MOESM3_ESM.zip › Additional File 3A/llmg_0195_real_dat.png]

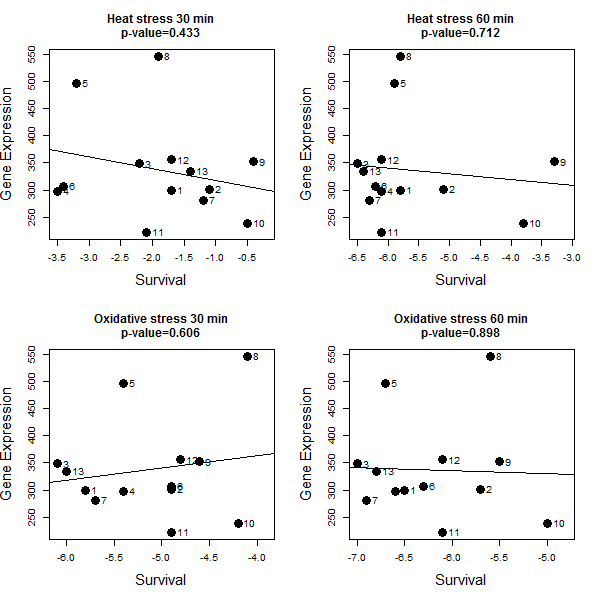

Supplement: Additional file 3: — Plots of gene expression and robustness levels. Expression levels of all genes plotted against survival after 30 and 60 minutes heat and oxidative stress (A: genes llmg_0001 to llmg_1229, B: genes llmg_1230 to llmg_2563). Survival is expressed as the difference of log CFU/ml after stress and before stress. Numbers indicate fermentations as presented in Table 1. P-values above the plots indicate significance of correlation (assessed by a linear model). [file 12934_2014_148_MOESM3_ESM.zip › Additional File 3A/llmg_0196_real_dat.png]

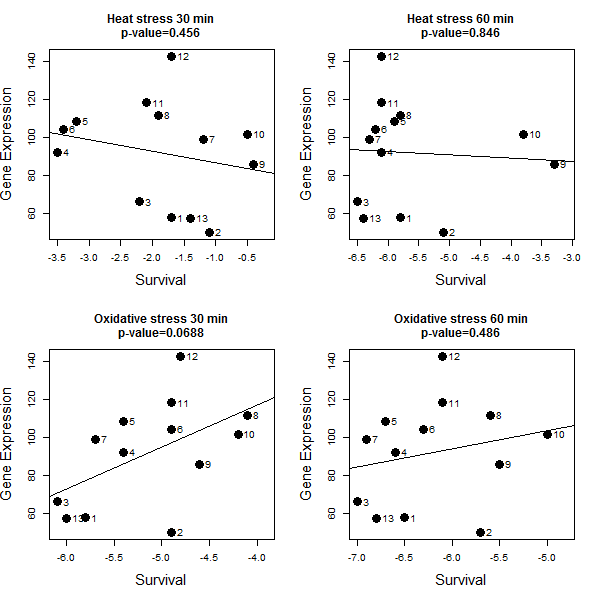

Supplement: Additional file 3: — Plots of gene expression and robustness levels. Expression levels of all genes plotted against survival after 30 and 60 minutes heat and oxidative stress (A: genes llmg_0001 to llmg_1229, B: genes llmg_1230 to llmg_2563). Survival is expressed as the difference of log CFU/ml after stress and before stress. Numbers indicate fermentations as presented in Table 1. P-values above the plots indicate significance of correlation (assessed by a linear model). [file 12934_2014_148_MOESM3_ESM.zip › Additional File 3A/llmg_0197_real_dat.png]

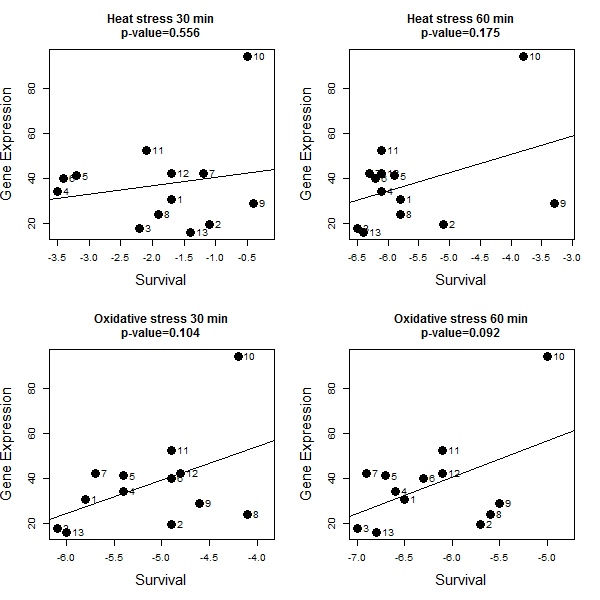

Supplement: Additional file 3: — Plots of gene expression and robustness levels. Expression levels of all genes plotted against survival after 30 and 60 minutes heat and oxidative stress (A: genes llmg_0001 to llmg_1229, B: genes llmg_1230 to llmg_2563). Survival is expressed as the difference of log CFU/ml after stress and before stress. Numbers indicate fermentations as presented in Table 1. P-values above the plots indicate significance of correlation (assessed by a linear model). [file 12934_2014_148_MOESM3_ESM.zip › Additional File 3A/llmg_0198_real_dat.png]

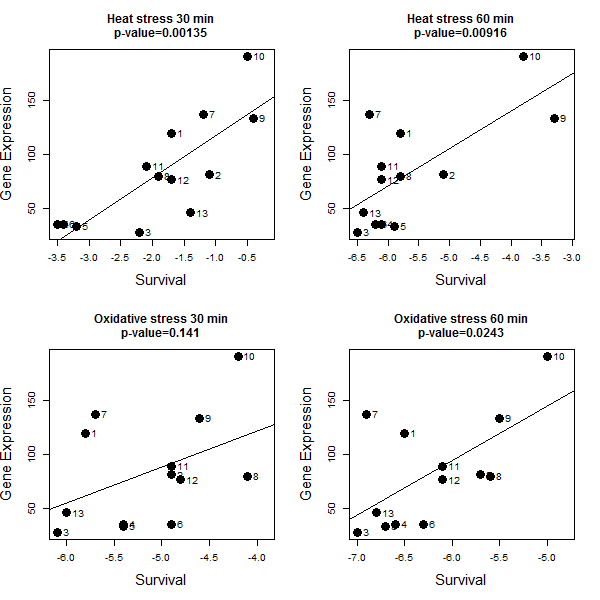

Supplement: Additional file 3: — Plots of gene expression and robustness levels. Expression levels of all genes plotted against survival after 30 and 60 minutes heat and oxidative stress (A: genes llmg_0001 to llmg_1229, B: genes llmg_1230 to llmg_2563). Survival is expressed as the difference of log CFU/ml after stress and before stress. Numbers indicate fermentations as presented in Table 1. P-values above the plots indicate significance of correlation (assessed by a linear model). [file 12934_2014_148_MOESM3_ESM.zip › Additional File 3A/llmg_0199_real_dat.png]

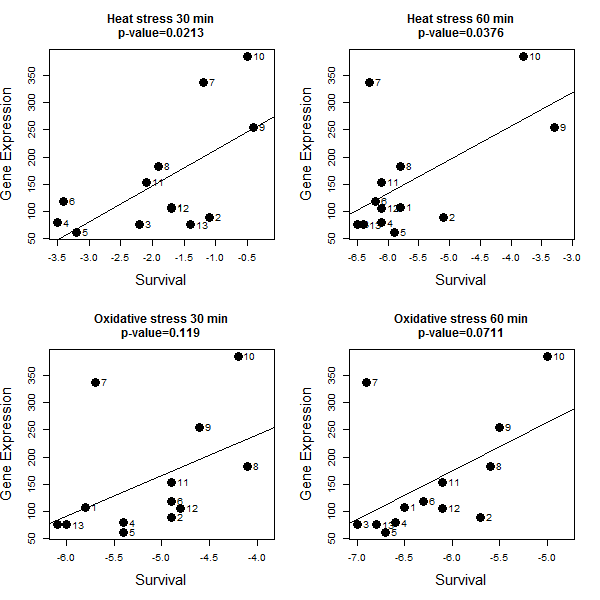

Supplement: Additional file 3: — Plots of gene expression and robustness levels. Expression levels of all genes plotted against survival after 30 and 60 minutes heat and oxidative stress (A: genes llmg_0001 to llmg_1229, B: genes llmg_1230 to llmg_2563). Survival is expressed as the difference of log CFU/ml after stress and before stress. Numbers indicate fermentations as presented in Table 1. P-values above the plots indicate significance of correlation (assessed by a linear model). [file 12934_2014_148_MOESM3_ESM.zip › Additional File 3A/llmg_0200_real_dat.png]

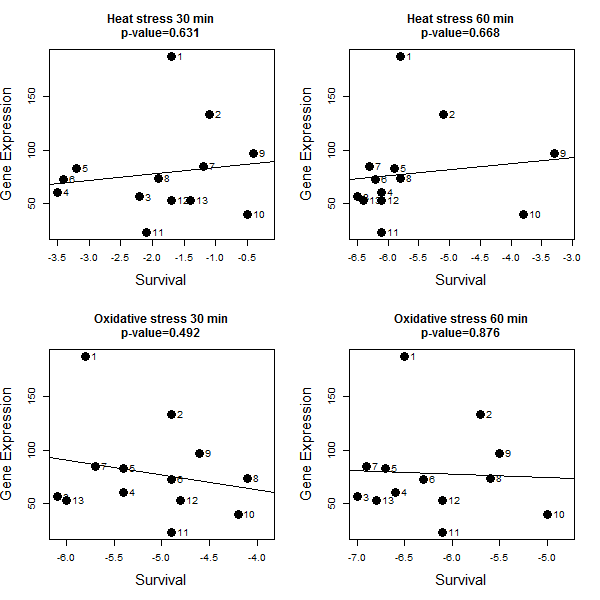

Supplement: Additional file 3: — Plots of gene expression and robustness levels. Expression levels of all genes plotted against survival after 30 and 60 minutes heat and oxidative stress (A: genes llmg_0001 to llmg_1229, B: genes llmg_1230 to llmg_2563). Survival is expressed as the difference of log CFU/ml after stress and before stress. Numbers indicate fermentations as presented in Table 1. P-values above the plots indicate significance of correlation (assessed by a linear model). [file 12934_2014_148_MOESM3_ESM.zip › Additional File 3A/llmg_0201_real_dat.png]

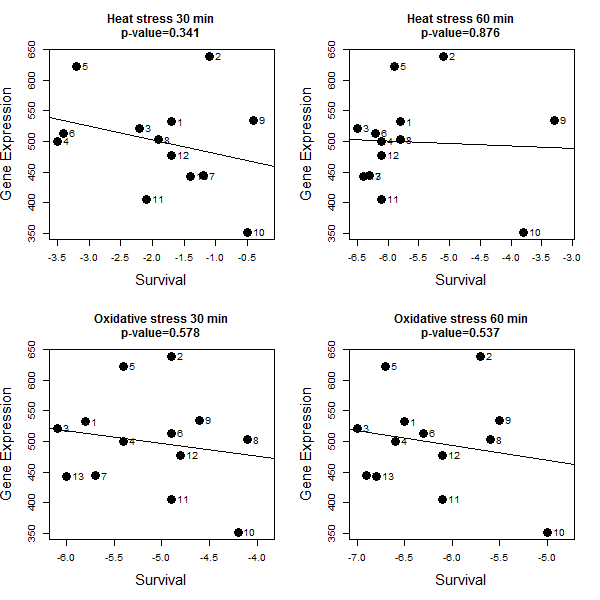

Supplement: Additional file 3: — Plots of gene expression and robustness levels. Expression levels of all genes plotted against survival after 30 and 60 minutes heat and oxidative stress (A: genes llmg_0001 to llmg_1229, B: genes llmg_1230 to llmg_2563). Survival is expressed as the difference of log CFU/ml after stress and before stress. Numbers indicate fermentations as presented in Table 1. P-values above the plots indicate significance of correlation (assessed by a linear model). [file 12934_2014_148_MOESM3_ESM.zip › Additional File 3A/llmg_0202_real_dat.png]

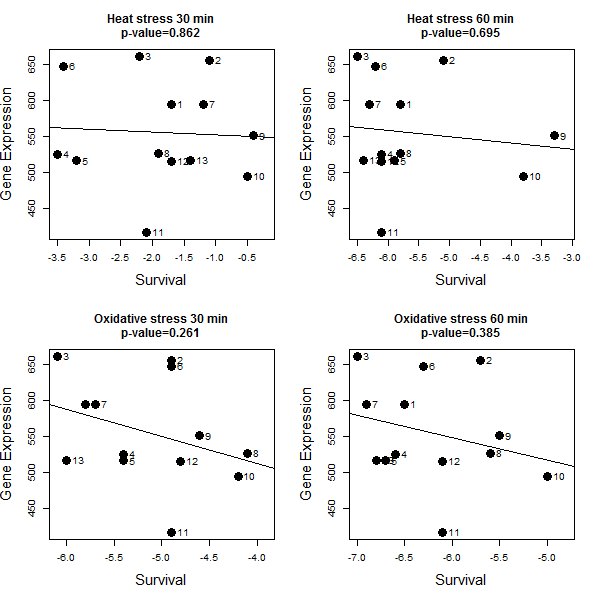

Supplement: Additional file 3: — Plots of gene expression and robustness levels. Expression levels of all genes plotted against survival after 30 and 60 minutes heat and oxidative stress (A: genes llmg_0001 to llmg_1229, B: genes llmg_1230 to llmg_2563). Survival is expressed as the difference of log CFU/ml after stress and before stress. Numbers indicate fermentations as presented in Table 1. P-values above the plots indicate significance of correlation (assessed by a linear model). [file 12934_2014_148_MOESM3_ESM.zip › Additional File 3A/llmg_0203_real_dat.png]

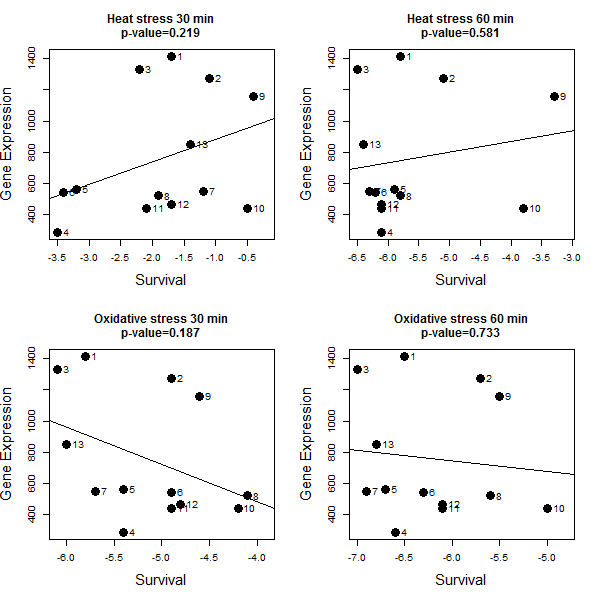

Supplement: Additional file 3: — Plots of gene expression and robustness levels. Expression levels of all genes plotted against survival after 30 and 60 minutes heat and oxidative stress (A: genes llmg_0001 to llmg_1229, B: genes llmg_1230 to llmg_2563). Survival is expressed as the difference of log CFU/ml after stress and before stress. Numbers indicate fermentations as presented in Table 1. P-values above the plots indicate significance of correlation (assessed by a linear model). [file 12934_2014_148_MOESM3_ESM.zip › Additional File 3A/llmg_0204_real_dat.png]

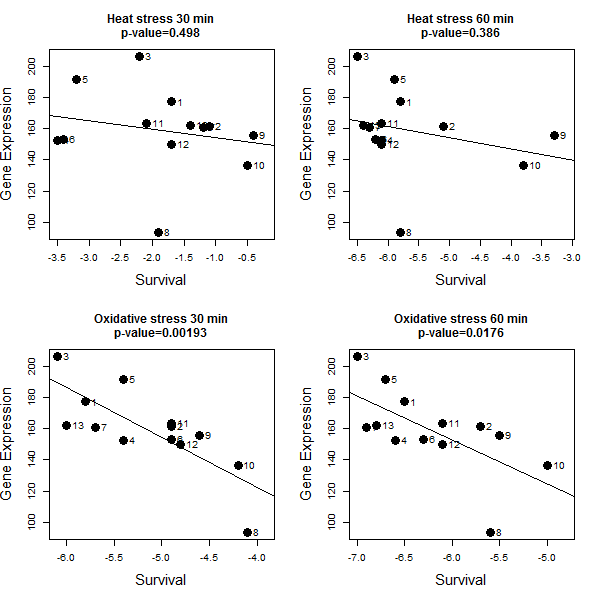

Supplement: Additional file 3: — Plots of gene expression and robustness levels. Expression levels of all genes plotted against survival after 30 and 60 minutes heat and oxidative stress (A: genes llmg_0001 to llmg_1229, B: genes llmg_1230 to llmg_2563). Survival is expressed as the difference of log CFU/ml after stress and before stress. Numbers indicate fermentations as presented in Table 1. P-values above the plots indicate significance of correlation (assessed by a linear model). [file 12934_2014_148_MOESM3_ESM.zip › Additional File 3A/llmg_0205_real_dat.png]

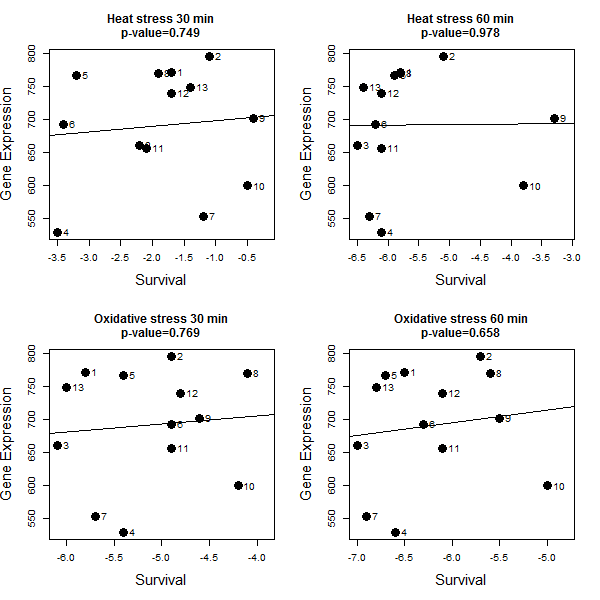

Supplement: Additional file 3: — Plots of gene expression and robustness levels. Expression levels of all genes plotted against survival after 30 and 60 minutes heat and oxidative stress (A: genes llmg_0001 to llmg_1229, B: genes llmg_1230 to llmg_2563). Survival is expressed as the difference of log CFU/ml after stress and before stress. Numbers indicate fermentations as presented in Table 1. P-values above the plots indicate significance of correlation (assessed by a linear model). [file 12934_2014_148_MOESM3_ESM.zip › Additional File 3A/llmg_0206_real_dat.png]

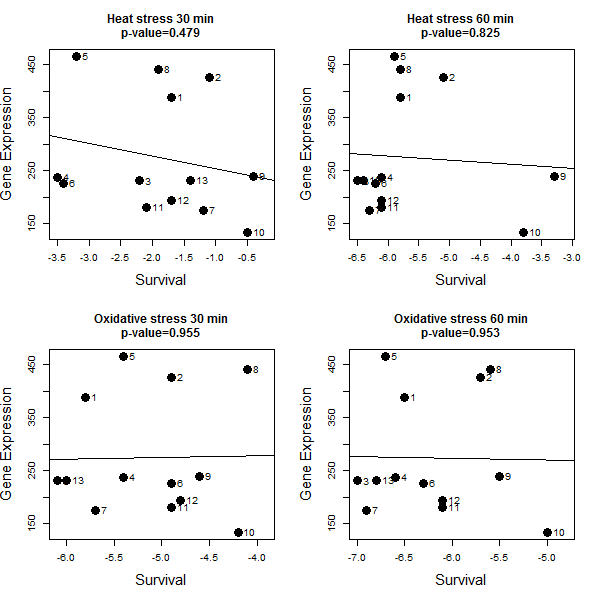

Supplement: Additional file 3: — Plots of gene expression and robustness levels. Expression levels of all genes plotted against survival after 30 and 60 minutes heat and oxidative stress (A: genes llmg_0001 to llmg_1229, B: genes llmg_1230 to llmg_2563). Survival is expressed as the difference of log CFU/ml after stress and before stress. Numbers indicate fermentations as presented in Table 1. P-values above the plots indicate significance of correlation (assessed by a linear model). [file 12934_2014_148_MOESM3_ESM.zip › Additional File 3A/llmg_0207_real_dat.png]

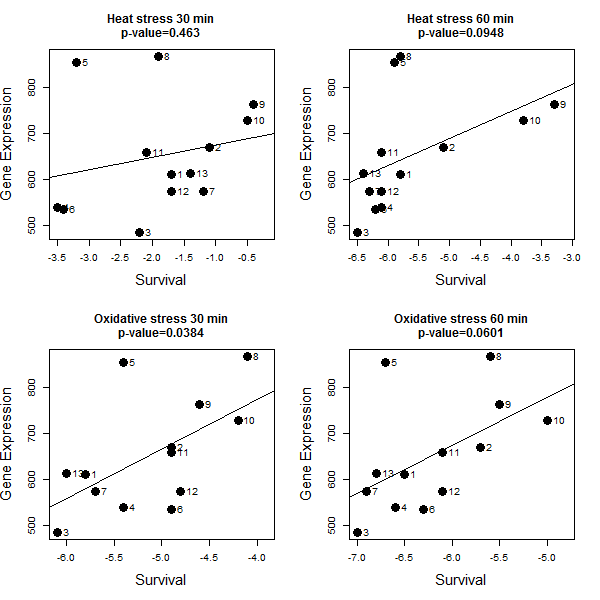

Supplement: Additional file 3: — Plots of gene expression and robustness levels. Expression levels of all genes plotted against survival after 30 and 60 minutes heat and oxidative stress (A: genes llmg_0001 to llmg_1229, B: genes llmg_1230 to llmg_2563). Survival is expressed as the difference of log CFU/ml after stress and before stress. Numbers indicate fermentations as presented in Table 1. P-values above the plots indicate significance of correlation (assessed by a linear model). [file 12934_2014_148_MOESM3_ESM.zip › Additional File 3A/llmg_0208_real_dat.png]

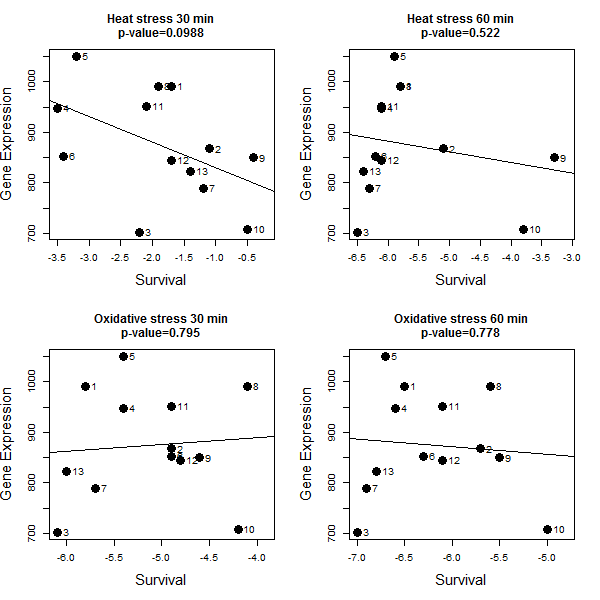

Supplement: Additional file 3: — Plots of gene expression and robustness levels. Expression levels of all genes plotted against survival after 30 and 60 minutes heat and oxidative stress (A: genes llmg_0001 to llmg_1229, B: genes llmg_1230 to llmg_2563). Survival is expressed as the difference of log CFU/ml after stress and before stress. Numbers indicate fermentations as presented in Table 1. P-values above the plots indicate significance of correlation (assessed by a linear model). [file 12934_2014_148_MOESM3_ESM.zip › Additional File 3A/llmg_0209_real_dat.png]

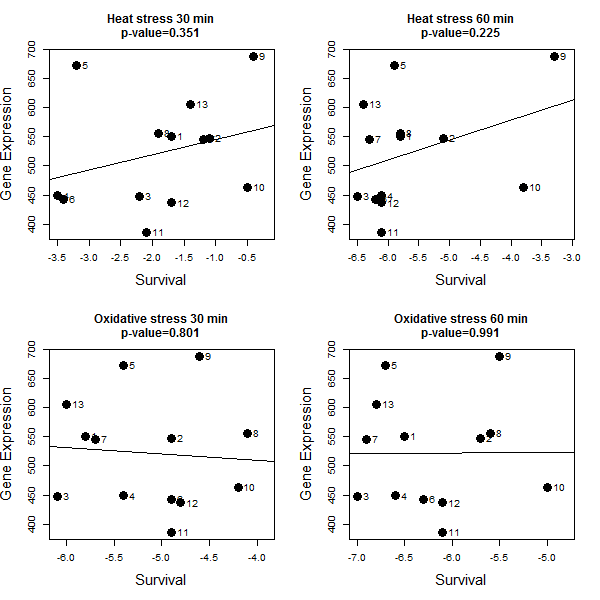

Supplement: Additional file 3: — Plots of gene expression and robustness levels. Expression levels of all genes plotted against survival after 30 and 60 minutes heat and oxidative stress (A: genes llmg_0001 to llmg_1229, B: genes llmg_1230 to llmg_2563). Survival is expressed as the difference of log CFU/ml after stress and before stress. Numbers indicate fermentations as presented in Table 1. P-values above the plots indicate significance of correlation (assessed by a linear model). [file 12934_2014_148_MOESM3_ESM.zip › Additional File 3A/llmg_0210_real_dat.png]
